# A multi-detector analytical approach for characterizing complex botanical extracts: a case study on ashwagandha

**DOI:** 10.1007/s00216-025-06006-8

**Published:** 2025-07-22

**Authors:** Vincent P. Sica, Constance A. Mitchell, Julie Krzykwa, Timothy R. Baker, Suramya Waidyanatha

**Affiliations:** 1 Procter and Gamble, Mason, OH USA; 2 Health and Environmental Sciences Institute, Washington, DC USA; 3 Division of Translational Toxicology, National Institute of Environmental Health Sciences, Research Triangle Park, NC USA

**Keywords:** Complex mixtures, Multi-detector platform, Ashwagandha root extract, Ultra-high-performance liquid chromatography

## Abstract

A comprehensive analytical characterization of botanical extracts can be difficult due to their complexity, dynamic range, and limited availability of constituent standards. By using ashwagandha root extract as a case study, this work showcases how to characterize a botanical extract utilizing an analytical system with multiple detectors to support the development of toxicological tools for evaluating botanicals. The platform incorporated ultra-high-performance liquid chromatography (UHPLC) coupled with photodiode array (PDA) detection, charged aerosol detection (CAD), and high-resolution mass spectrometry (HRMS) detection to generate a detailed chemical profile. This multi-detector platform enabled both semi-quantification and the identification of the majority of constituents, ensuring accurate chemical analysis while compensating for potential detector biases. The approach provided a thorough fingerprint of the ashwagandha extract, enabling authentication of the material. The generation of a comprehensive fingerprint supports in silico modeling and bioassay-based toxicological evaluations of botanicals, particularly in the context of dietary supplement safety. This study demonstrated the utility of detailed chemical analysis supporting the authenticity of ashwagandha root extract and the advancement of tools needed for robust safety assessments of botanical products by providing the semi-quantification and identification of over 60 constituents in ashwagandha extracts.

**Graphical Abstract:**

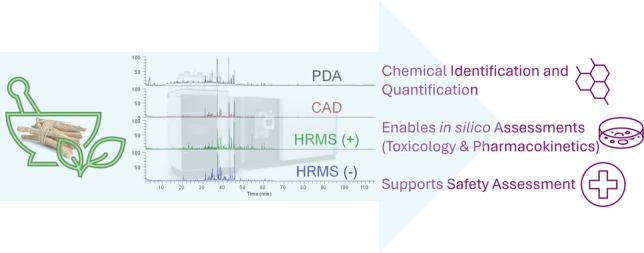

**Supplementary Information:**

The online version contains supplementary material available at 10.1007/s00216-025-06006-8.

## Introduction

Botanical dietary supplements and herbal medicines have become increasingly popular in recent years, both in the USA and globally [1]. In some cultures and subpopulations, botanicals are used routinely for disease prevention and treatment [2]. The USA alone has seen an increase in the use of nonvitamin, nonmineral dietary supplements, with over 18% of adults reporting their use [2]. Botanicals contain hundreds to thousands of individual chemicals. Some of these chemicals serve protective roles and support the survival of the plant (called phytochemicals) [3]. Many of these phytochemicals have been used by humans as pharmaceuticals, pesticides, natural dyes, poisons, or other applications [4]. Products derived from botanicals can be variable in phytochemical composition both qualitatively and quantitatively. This variability can come from growing conditions (e.g., seasonal changes, soil composition) and process (e.g., extraction type, purification) differences [5]. Furthermore, botanical products come in many different forms, such as capsules, gummies, powders, teas, and tinctures, which can change the amount of a constituent and the rate of exposure to the chemicals.

While rare, adverse events have been reported when taking dietary supplements or other natural products [6]. For example, ephedra formulations were associated with cardiotoxicity, especially when taken with caffeine. This led to multiple deaths, and ephedra was subsequently banned in the USA [7, 8]. Additionally, plants that are likely safe in their traditional use may lead to adverse events in concentrated forms. Green tea is one of the most popular drinks in the world and is thought to have health benefits. However, there are case reports that concentrated green tea extracts with high levels of catechins have been associated with hepatotoxicity [9]. Additionally, adulterations can lead to toxicity in humans from botanicals [10]. Adulteration can be intentional (e.g., mixing in a less expensive botanical extract with a “similar” chemical profile) or accidental (e.g., cross-contamination with another botanical during extraction), and can be difficult to detect without the proper tools.

There is a need to ensure the safety of these products. However, based on the variability described above, it is difficult to perform traditional toxicity testing on a single test article or individual constituents to represent the array of products that may be on the market. Given the intentional exposure and possibility of adverse events, it is important to evaluate the toxicity of botanicals used in dietary supplements and herbal medicines to ensure public safety [5]. The Botanical Safety Consortium (BSC) [www.botanicalsafetyconsortium.org] brings together experts from research institutes, industry, and government agencies worldwide to evaluate the safety of botanicals.

To be able to accurately interpret the biological assay results and predict the safety of botanicals, an understanding of the chemical make-up of the botanical is needed [11]. For all selected botanical case studies, the BSC obtained materials to test in multiple assays and chemical analyses. For the chemical analyses, a detailed strategy can be found in Waidyanatha et al. 2024, and the results for individual botanical materials can all be found on CEBs https://cebs-ext.niehs.nih.gov/cebs/paper/15717. Chemical analyses of botanicals using appropriate analytical methods are essential to ensure that the material is authentic and contains botanical-specific constituents at the expected concentrations. State-of-the-art analytical platforms are needed to accomplish this due to the presence of numerous constituents with different properties. The multi-detector system (information given below) allows for comprehensive characterization of these complex mixtures.

To demonstrate the utility of the system, ashwagandha (*Withania somnifera* L. Dunal (Solanaceae)) root extract was selected because experts had existing analytical methodologies and experience with the plant and extract. Ashwagandha is a plant commonly used in traditional Ayurvedic medicine [12]. The plant is a branched shrub with green to yellow flowers, which then turn into small, spherical, orange-red berries: the root is most commonly used in herbal preparations. Ashwagandha root is typically dried, ground, and subjected to alcohol extraction, followed by concentration, filtration, and standardization to ensure consistent withanolide content [13]. It is available in a variety of forms, including capsules, powders, and tinctures. While there have been adverse events related to hepatotoxicity in recent years [14, 15], there is existing data suggesting ashwagandha root extract does not cause overt toxicity [16, 17]. Differences in biological effects, while beyond the scope of this manuscript, could be due to differing chemical constituents, life stage of users, dosage, botanical-drug interactions, and potentially due to adulterations. According to the NIH Office of Dietary Supplements, ashwagandha in clinical trials has been well tolerated by participants up to about 90 days; however, common side effects can include upset stomach, GI issues, and drowsiness [18]. There is ongoing research related to the safety of ashwagandha.

**Fig. 1:**
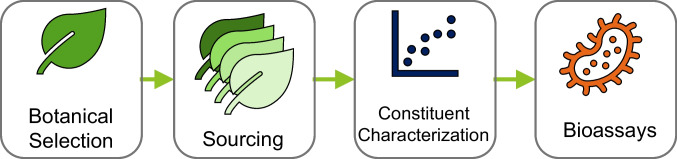
Overall strategy to select, source, and support evidence for the authenticity and chemical characterization of ashwagandha root extract. Figure adapted from Waidyanatha et al. 2024

A multi-detector approach was implemented that consists of three detectors providing four fingerprints for each sample. This platform combines several powerful detection techniques to ensure comprehensive analysis by leveraging industry standard photodiode array (PDA or UV–Vis) detection, charged aerosol detection (CAD) for semi-universal quantification, and both positive and negative high-resolution mass spectrometry (HRMS) for identification of constituents. This setup ensures the ability to analyze a wide range of constituents with high accuracy and resolution. Additionally, an inverse gradient make-up flow is implemented to maintain a consistent mobile phase for the CAD detector to compensate for any potential detector biases. In this manuscript, we describe the chemical characterization and constituent quantification of ashwagandha root extract. Three lots of ashwagandha root extract from different suppliers were screened and compared to the USP extract, of which one was selected for extensive characterization. The general process is described in Fig. 1 for how we see constituent characterization fitting into the larger scheme for botanical safety [11].

This study supports the development and use of high-quality chemical data to improve in silico and in vitro toxicological tools for botanical safety assessment, using ashwagandha root extract as an example.

## Materials and methods

### Ashwagandha root extracts

Four lots of ashwagandha root extract were obtained by different sources: one USP-grade ashwagandha (powdered) extract was purchased from Millipore Sigma (St. Louis, MO, USA) for use as a reference material and three additional extracts from reputable suppliers to represent material sourcing options.

### Sample preparation

The ashwagandha extracts were prepared in 50:50 methanol–water to afford a 20 mg/mL solution. The samples were vortex mixed (60 s), sonicated for 5 min, and vortex mixed (60 s) again. The samples were centrifuged for 10 min prior to transferring to an autosampler vial.

### Chemical standards

USP-grade tropine [DTXSID2049399], pseudotropine [DTXSID501026533], kaempferol-ß-O-robinoside-7-O-glucoside [USP 1354954], withanolide A [DTXSID70461487], withanoside IV [DTXSID40746874], withanone [DTXSID40950237], and withanoside V [DTXSID20443612] standards were purchased from Millipore Sigma (St. Louis, MO, USA). The HPLC grade methanol, acetonitrile, water, and ethanol were purchased from Honeywell (Morris Plains, NJ, USA).

### Standard preparation

Individual stock solutions were prepared at 1.0 mg/mL in 50:50 methanol–water. Two separate combined high standard mixes (100 μg/mL each component) were prepared by adding 0.100 mL of each standard and diluted to 1 mL with 50:50 methanol–water. Standard mixes were split into two sets: (A) tropine, withanolide A, withanoside IV, and kaempferol-ß-O-robinoside-7-O-glucoside, and (B) pseudotropine, withanone, and withanoside V to avoid co-elution and allow for differentiation of isomers. Each mix was diluted to 20, 4, and 0.8 μg/mL with 50:50 methanol–water by serial dilution.

### Instrumentation

The ultra-high-performance liquid chromatography (UHPLC)–PDA–CAD–HRMS system used was a Vanquish dual ternary pump (VF-P32-A-01), with a Vanquish PDA detector (VF-D11-A), split to a Vanquish CAD (VH-D20-A) and Orbitrap ID-X mass spectrometer (Thermo Fisher, San Jose, CA, USA). The mass spectrometer was set to collect *m*/*z* 125–2000 at a resolution of 120,000 at *m*/*z* 200. Positive and negative ionization modes were run as separate injections. Each polarity included data-dependent acquisition alternating collision-induced dissociation (CID) and higher-energy collisional dissociation (HCD) fragmentation set to 30 for all compounds. The voltage for positive and negative ionization modes was set to 3.5 and 2.5 kV, respectively, with a nitrogen sheath gas set to 35, an auxiliary gas at 7, and a sweep gas at 0. The S-Lens RF level was set to 60.0, and the capillary temperature was set to 300 °C. A Hypersil Gold aQ (25,302–152130-V) 2.1 × 150 mm, 1.9 µm column (Thermo Fisher, San Jose, CA, USA) was used with a mobile phase flow rate of 400 µL/min. The mobile phases were water (acidified with 0.1% formic acid) and methanol starting at 0% methanol for 5 min, increased linearly to 100% over 100 min, held for 10 min, then re-equilibrated at 0% methanol. Since CAD response factors are affected by changes in the mobile phase, an inverse gradient was connected prior to splitting into the CAD and mass spectrometer to compensate for changes in the gradient to maintain a composition of 50:50 of each mobile phase [19]. The CAD power function was set to 1.5 with an evaporator temperature of 35 °C. The PDA was set to acquire from 190 to 800 nm with a 4-nm resolution.

## Results and discussion

### Ashwagandha root extract comparison

Finding a trustworthy and authentic source of plant material is crucial for evaluating the biological properties of botanicals. Although plant material or products deriving from them can be obtained from various sources, such as grocery stores, health food stores, herbal companies, and online suppliers, authenticity or potential adulteration (i.e., economically motivated or unintentional) of such material is typically unknown. The botanical material must belong to the correct genus and species and be derived from the appropriate plant part. In this case, lots of ashwagandha root extract were obtained from three reputable suppliers recommended by experts within the BSC. Apart from prioritizing reputable suppliers, we also employed methods to verify the authenticity of the botanicals obtained [11].

The ashwagandha root extracts were subjected to a fingerprinting analysis by comparing the chromatographic traces for each detector on the UHPLC-PDA-CAD-HRMS platform. Qualitatively, all four extracts were considered essentially equivalent, with differences only in relative abundances of some signals (Figure SI 1). Thus, the three reliable suppliers met expectations, and of these, one was selected for comprehensive analysis.

### Constituent characterization

Botanicals are complex mixtures with a variety of constituents, and for most constituents, synthetic standards are not available. Under these circumstances, the integration of chromatographic separation coupled to a multi-detector platform can be employed to conduct a thorough chemical constituent identification (CCID) assessment and quantitation of a botanical extract. Utilizing UHPLC coupled to a multi-detector platform helps ensure that the CCID is comprehensive. First, a PDA detector incorporates a non-destructive technique that can provide some quantitative and qualitative information for any constituents that have a chromophore. However, alone it lacks the ability to detect even the most common chemical classes that are predominant in botanicals, such as sugars and fatty acids. It also has limited qualitative (spectral) information and quantitatively still requires a standard for each class. The addition of HRMS enables an abundance of qualitative and quantitative information for chemical characterization. Leveraging its incredibly sensitive, accurate mass measurements, and fragmentation capabilities, HRMS greatly strengthens the ability to identify each constituent. However, not all constituents can ionize, making it insufficient as a universal quantitative detector for complex mixtures. Since even slight modifications of a constituent can affect its ionization efficiency, quantification via mass spectrometry often requires a standard for each individual constituent to be most effective. Adding charge aerosol or evaporative light scattering detectors can provide unbiased, semi-universal detection of all the constituents in a complex mixture. For instance, CAD does not rely on the chemical structure, unlike PDA or MS which rely on a chromophore or ionization efficiency, respectively [20, 21]. CAD enables semi-universal detection, allowing for the semi-quantification of individual constituents in botanical extracts or other complex mixtures without the need for standards for each constituent or class of constituents. Since CAD relies on aerosolization and desolvation, this technique is only appropriate for non-volatiles and some semi-volatile constituents. Thus, complementary techniques (e.g., GC/MS, GC/FID) may be required if more volatile constituents are known to be present and/or if you obtain a low mass balance.

By integrating the UHPLC-PDA-CAD-HRMS platform, four orthogonal sets of data from the three detectors can be obtained, providing a comprehensive analysis of each sample. This multi-detector approach allows for a more holistic evaluation of the chemical composition, offering a complementary detection system, ensuring a thorough and accurate assessment of the constituents present in the sample.

Leveraging this multi-detector platform, a comprehensive chemical constituent identification (CCID) was performed for ashwagandha by executing the following key steps:

### Experimental design

During the method development of such characterization projects (quantification and identification), the goal is not speed and sensitivity that are typical with an HPLC–MS method. Rather than trace-level quantification and high-throughput, the most effective CCID methods involve the utilization of long gradual gradients (> 30 min) and high sample concentrations (> 10 mg/mL). Achieving semi-quantification on complex mixtures requires careful consideration and several key steps in the methodology design.

Firstly, since the charged aerosol detector (CAD) is generally less sensitive than mass spectrometry (MS), the samples often need to be prepared at higher concentrations to ensure any pertinent thresholds can be met. With a CAD limitation of about 1 µg/mL for a given constituent, a concentration of approximately 10–20 mg/mL of an extract often serves as a suitable starting point to see a majority of the constituents for a CCID. With the CAD demonstrating linearity over several orders of magnitude, standard curves for this analysis typically span ng/mL to upwards of 100 µg/mL. Once again, since the CAD can act as a semi-universal detector, the standards used do not necessarily need to be inherent to the botanical, although that is preferred when possible. The ashwagandha samples were prepared in triplicate at 20 mg/mL. To avoid co-elution and ambiguity (i.e., differentiation of isomers), two sets of standards were prepared at concentrations of constituents ranging from 0.8 to 100 µg/mL: (1) tropine, withanolide A, withanoside IV, and kaempferol-ß-O-robinoside-7-O-glucoside, and (2) pseudotropine, withanone, and withanoside V. The use of authentic standards of analytes known to be in the botanical has the additional useful benefit of illustrating the UV–Vis and mass spectral (MS/MS) characteristics of analyte classes of interest. This facilitates the identification of related analytes, for which there are no standards. However, for less well-studied botanicals, any non-volatile standards could be suitable for a study.

**Fig. 2:**
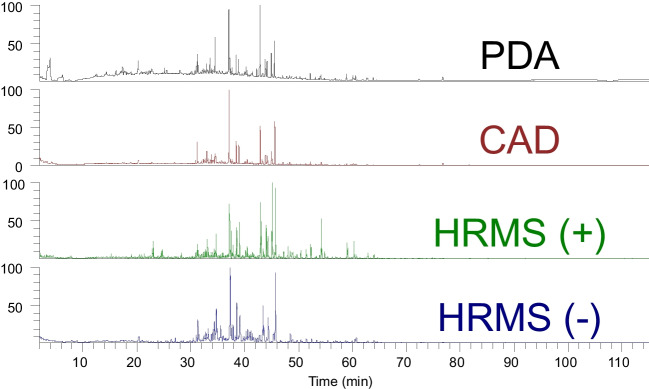
The chromatograms generated from a single injection across the multi-detector platform

Furthermore, contrary to common high-throughput HPLC–MS methods, here the goal is to separate the peaks as much as possible since the quantification relies solely on the CAD, which lacks specificity and qualitative information. This approach ensures that the semi-quantification is as unambiguous as possible. Consequently, longer chromatography gradients (often over 60 min) that span a wide polarity range are often employed to achieve optimal separation and resolution. For this study of ashwagandha, a gradual gradient (1%/min) was employed to ensure maximum separation and several fingerprints across the multiple detectors (Fig. 2).

### Semi-quantification

This methodology uses surrogate standards to estimate the quantity of constituents in complex mixtures via a semi-quantitative fashion, with a goal of achieving an approximation rather than the accuracy and precision typically achieved in targeted quantification. This was suitable (fit-for-use) to support the authentication of this botanical, especially considering the natural variability of samples themselves and the application of this process. Upon completion of the chromatographic sequence, the CAD standard curve(s) were subjected to an evaluation of linearity, and the response factors (RF) were calculated for each standard at various concentrations (RF = $\frac{\mathrm{Area}}{\mathrm{Concentration}}$). Standard curves for each analyte exhibiting an *r*^2^ value greater than 0.99 and a relative standard deviation (RSD) of the response factors below 25% (as were the case in this study) are typically considered acceptable for the semi-quantitative analysis of botanicals (Table SI 1). By averaging all the response factors, a relative response factor (RRF) can be generated, which can be applied to all peaks observed in the sample’s chromatogram(s). Thus, every peak present in the sample’s CAD chromatogram can be integrated, and its concentration can be determined using the RRF (Tables 1 and 2).

**Table 1:** Calculation of the RRF based on the seven analytes used for CAD semi-quantification. Additional details for the individual standards can be found in Table SI 1

Concentration (µg/mL)	Average area	RSD	Average response factor
0.8	0.43	16%	0.54
4	2.3	17%	0.58
20	13	16%	0.66
100	69	11%	0.69
Relative response factor (RRF)			0.62
RSD of average RF			11%

**Table 2:** Example calculation determining the amount of constituent in extract

Peak #	RT	Area	RRF^a^	µg const./mL^b^	mg extract/mL^c^	µg const./mg extract
26a	37.15	84	0.62	136	20	6.8

^a^Calculated in Table 1. ^b^Area/RFF ^c^Concentration of extract prepared

**Fig. 3:**
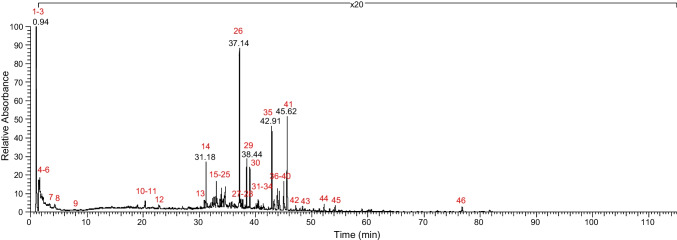
CAD chromatogram labeling the 46 quantified peaks that were above the AET (6.75 µg/mL) for ashwagandha

Once all the sample’s peaks were integrated, the RRF was applied, and all the peaks were quantified. In cases where a reference standard used to calculate the RFF is also a constituent of the botanical, an exercise can be performed to support the semi-quantified value. For instance, a constituent can be semi-quantified using the RFF (all standards averaged and combined), the RF of just that constituent, and the RF of the accurate mass measurements. In this case study, this was performed for withanolide A and withanoside V and determined that all three measurements were within 25% of each other (Table SI2). At this point, CCID of the extract can be performed. If a threshold is desired, for instance to support a risk assessment of the material, a limit can be applied and only those peaks exceeding the limit necessitate identification. In this case study for ashwagandha, the Cramer Class III (food and cosmetics) threshold of toxicological concern (TTC) was leveraged at 90 µg/day [22, 23]. Based on the TTC, an analytical evaluation threshold (AET) was calculated to determine which CAD peaks were above the threshold (Eq. 1). Thus, there were 46 CAD peaks above the threshold that require characterization (Fig. 3). Finally, it was determined that this evaluation of ashwagandha had a mass balance of 99% by summing the calculated amount of each constituent (Table SI3) for the prepared concentration (20 mg/mL).

Conversion of TTC to AET based on the concentration used for analysis and an expected dose.

1 $$\begin{array}{ccccc}TTC&Dose&Extraction\;ratio&Concentration&AET\\\frac{90\;\mu g}{day}\times&\frac{day}{4000\;mg\;of\;ashwagandha\;}\times&\frac{15\;g\;ashwagandha}{1\;g\;extract}\times&\frac{20\;mg\;of\;extract}{mL}=&\frac{6.75\mu g}{mL}\end{array}$$

### Correlation

Once the number of relevant CAD peaks was determined, the mass spectral signals were correlated to each CAD peak. This correlation was achieved by summing the mass spectra at the retention times across the CAD peak to generate a summed mass spectrum [19]. Utilizing this summed mass spectrum as a reference, signals were correlated to identify adducts, in-source fragments, and separate co-eluting signals. This process was used to create a list of potential analytes that contribute to the CAD peak. To ascertain the contribution of an analyte to the CAD signal, narrow mass chromatograms or extracted ion chromatograms (XIC) were generated to determine if an analyte aligned with and contributes to the CAD signal or if it is a low-concentration co-eluting signal. It is important to note that the intensity of analytes may not always indicate their significance due to variable ionization efficiencies. In cases of uncertainty, we erred on the side of caution and characterized the constituent.

**Fig. 4:**
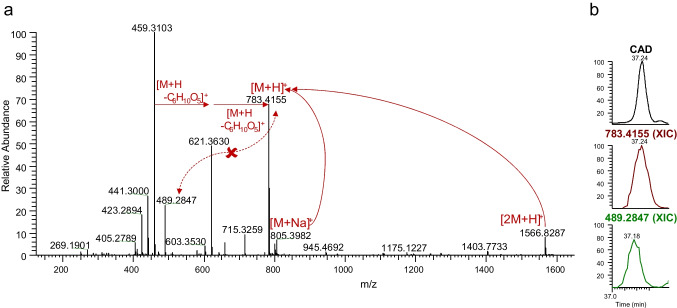
**a** Correlation of *m*/*z* signals to begin determining the number of constituents contributing to the quantified CAD peak 26. **b** Using extracted ion chromatograms to confirm alignment of the MS signals with the CAD peak and to determine if any adducts or in-source fragments were missed during the process

For example, under CAD peak #26 (Table SI 3), the summed mass spectrum (Fig. 4a) displayed several signals, primarily adducts ([M + Na]^+^, [2 M + H]^+^, etc.) and in-source fragments (sugar and water losses) related to the signal at *m*/*z* 783.4155. However, there was no apparent correlation to *m*/*z* 489.2847. To further investigate, an XIC was generated to compare the signals (Fig. 4b). Based on these chromatograms, the mass at *m*/*z* 489.2847 does not appear to be a major contributor to the CAD signal. Its retention time does not align, and there is no discernible influence on the CAD peak shape (e.g., shoulder, tailing). Nevertheless, since part of the signal does align, our conservative approach included both constituents in the final table (Table SI 3) as peaks 26a and 26b. Thus, it is important to note that the number of CAD peaks does not necessarily correspond to the number of constituents that need to be identified. This process led to an increased number of constituents requiring characterization but allowed for a comprehensive look at the extract’s constituency. Thus, the 46 CAD peaks for ashwagandha resulted in identification of over 60 constituents.

### Identification

After compiling the list of CAD peaks and correlating to the corresponding *m*/*z* signals, the characterization process commenced by leveraging accurate mass measurements to determine the molecular formula of each analyte. For example, the two accurate mass measurements for CAD peak 26 (*m*/*z* 783.4155 and 489.2847) correlated to neutral molecular formulae C_40_H_62_O_15_ and C_28_H_40_O_7_, respectively. These formulae served as a basis for searching structures of constituents, particularly those previously reported in relation to the botanical of interest, via structure and literature databases (e.g., ChemSpider, SciFinder). As expected, many of the determined molecular formulae were attributed to withanolides and withanosides, which are well-documented as constituents of ashwagandha in the existing literature [24–26].

**Fig. 5:**
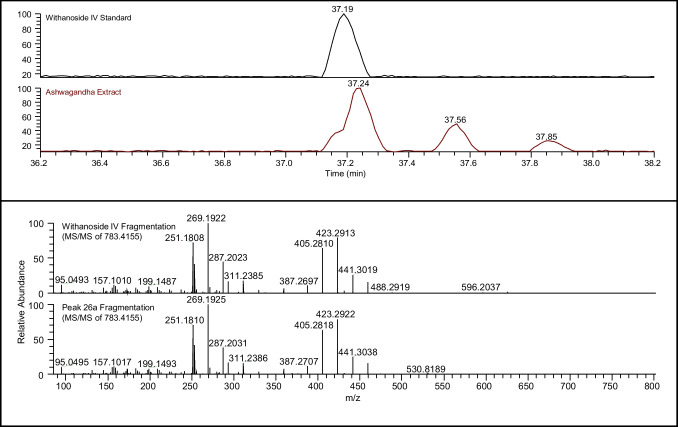
(Top) Comparison of the retention time (37.2 min) between the withanoside IV standard and peak 26. (Bottom) Comparison of the fragmentation of withanoside IV and the ashwagandha constituent 26a

Once a list of potential structures was generated, fragmentation experiments (such as MS/MS, MS^n^) were conducted and compared with data available in the literature or specialized spectral libraries (e.g., NIST, mzCloud, Mass-bank, METLIN). Manual interpretation, ideally supported by reported precedence, was also employed. Furthermore, where standards were available, acquiring and confirming the retention time of the analyte added an additional level of confidence (Fig. 5). Even when the retention time of the standard and peak in the extract does not match (as shown by the additional peaks in Fig. 5), it can provide valuable information from its elution time and fragmentation patterns, which can support other constituents, such as isomers. Similar to other confidence conventions [19, 27–29], by combining all of these data and observations, it becomes possible to assign increasing levels of confidence to each identification: Partial, Tentative, Matched, and Reference.

In this context, a Partial identification implies that a molecular formula has been derived from exact mass measurements. Tentative identifications have a molecular formula derived from exact mass measurements and a proposed structure supported by either an MS/MS spectrum, UV–Vis spectrum, or literature data reporting the structure in plants of the same genus (in this case, *Withania*). A Matched identification requires all the aforementioned elements (i.e., MS/MS, UV–Vis, etc.), as well as literature data reporting the structure in *Withania somnifera*, or a proposed structure supported by MS/MS with a match in a database, and the compound is reasonably likely to be present in the plant material. Finally, a Reference identification fulfills the same criteria as a “Matched” identification but also includes a retention time and MS/MS match to an authentic reference standard [19, 29]. Using this approach, CAD peak 26a was identified as withanoside IV with classification level reference and 26b was identified as viscosalactone B with a classification level matched, and complete data for these two peaks are shown in Table 3.

**Table 3:**
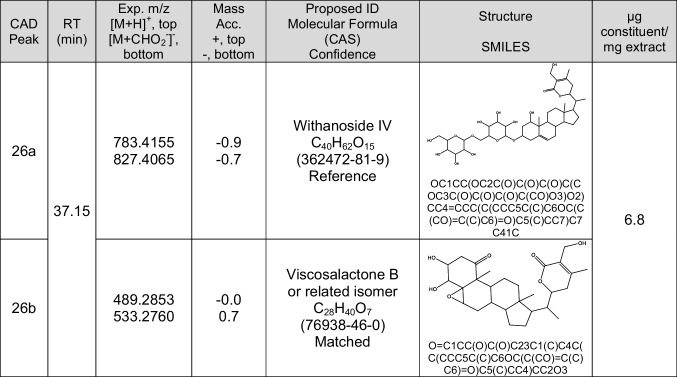
The semi-quantification, identifications, and confidence level for the constituents that co-eluted to contribute to CAD peak 26. Excerpt from Table SI 2

For each constituent, we assigned a confidence level and provided an explanation of how that level was derived (as indicated in the comments column of Table SI 2). Even in cases where only a partial identification is achieved, this confidence level assignment remains valuable. If a constituent has a lower level of confidence (e.g., partial), but its molecular formula matches a constituent of interest, further analyses can be conducted to determine a compound class, refine the characterization of the constituent, or rule out any ambiguity of related compounds of interest. Therefore, the assigned confidence level, even at a Partial identification, can still provide valuable insights for subsequent analyses and application of the data. Ultimately, there were 9 Partial, 26 Tentative, 21 Matched, and 6 Reference classifications for this ashwagandha extract with about 3.8% of the material consisting of withanolides, withanosides, and related analogues [24, 26–28, 30–41].

In addition to the withanolides and withanosides (~ 3.8% of the extract), the majority of the remaining mass balance was attributed to saccharides, which are known to be abundant in ashwagandha root extracts. Minor constituent classes (< 1%) included alkaloids such as tropine and pseudotropine derivatives. A complete breakdown of the identified constituents is available in Supplementary Table SI 2.

### Evaluation

Upon quantifying, characterizing, and assigning a confidence level to all constituents, the data was organized and presented in a tabulated format, such as the final output table (Table SI 2) and available online in a database [42]. What happens next with the data is determined by the purpose behind generating it. This comprehensive table can be leveraged in a number of ways, such as looking for low-level adulteration, looking for marker compounds, or by a toxicologist for an in silico risk assessment [43, 44]. Through follow-up assessments, if any constituents require further identification or characterization, they can be highlighted, and additional analytical experiments can be conducted.

For the purpose of this case study, the ashwagandha data was generated to showcase how to perform a comprehensive chemical constituent characterization process. However, there are several types of analytical re-evaluations that can be undertaken. Firstly, if there are any constituents of interest (i.e., adulterants, contamination, degradation products, etc.) that correspond to a molecular formula or necessitate a higher level of characterization, more targeted or focused experiments can be performed. These may include MS^n^ experiments, employing alternate fragmentation techniques (such as CID, HCD, UVPD), or employing derivatization techniques to advance the identification process [45–47].

Secondly, if a constituent of interest is in close proximity to or surpasses a known threshold, more precise quantification can be carried out. In the cases of co-elution, this can involve gaining a better understanding of each constituent’s contribution to the CAD signal. Another scenario is where the identified constituent’s volatility (boiling point) is nearing the limitation of the CAD (about < 350–400 °C); more accurate quantification can be pursued. These follow-up analyses often require either the availability of a standard to facilitate precise quantification or the modification of chromatography to focus solely on the region of interest.

By performing these supplementary analyses, it becomes possible to refine the characterization of constituents, address any uncertainties, and enhance the overall understanding of the complex mixture under evaluation, but are beyond the scope of this initial assessment.

## Conclusions

In conclusion, this study refined the use of a comprehensive multi-detector platform to identify and quantify the chemical constituents of ashwagandha root extract. By employing a combination of ultra-high-performance liquid chromatography (UHPLC), photodiode array (PDA) detection, charged aerosol detection (CAD), and high-resolution mass spectrometry (HRMS), the methodology allowed for detailed chemical profiling, ensuring a more robust and nuanced understanding of the botanical’s composition. This approach, used in conjunction with other techniques like high-performance thin-layer chromatography (HPTLC) data (available online via NIEHS [42]), enabled the authentication of the ashwagandha root extract, underscoring the importance of orthogonal data in assessing the authenticity and quality of botanical products.

The multi-detector platform employed in this study provides significant benefits for the broader application of botanical safety assessment. By offering a thorough chemical constituent identification process, this platform can be instrumental not only in verifying the authenticity of botanical materials but also in supporting various toxicological assessments, including in silico models and absorption, distribution, metabolism, and excretion (ADME) studies. The constituent identification and quantification data produced through this platform contribute to critical safety evaluations. For example, the BSC has utilized the data generated from this study and other chemical analyses of botanicals in conjunction with computational models to predict physicochemical and pharmacokinetic properties of botanical constituents [44], improving the relevance of in vitro toxicological findings to human health.

While the platform offers clear advantages, it is important to acknowledge certain limitations. This approach requires access to specialized equipment and expertise, which may not be readily available in all laboratories. However, these investments are justified by the speed and comprehensiveness of the data generated. The platform’s ability to potentially reduce reliance on time-intensive in vivo studies by providing high-quality constituent data promotes the use of in silico assessments and informs in vitro studies.

Future applications of this methodology could further explore the impact of chemical variability between different lots of botanical materials. By comparing batches of botanical extracts, researchers could gain insights into how variations in chemical content or concentration may influence biological outcomes. This is particularly relevant for understanding dose-dependent responses or identifying potential adulterants. Additionally, integrating chemical profiling data with biological assays could help refine the predictive models used in risk assessments for botanicals, ultimately enhancing public safety.

Given recent concerns about ashwagandha-induced liver injury [15, 48, 49], this analytical characterization provides critical insight into the main constituent classes, particularly withanolides and withanosides (~ 3.8% of the extract). These constituent classes are most associated with bioactivity and potential toxicity. By delivering a comprehensive chemical fingerprint, this approach supports safety evaluation by identifying known and novel constituents, detecting potential adulterants, and enabling more accurate interpretation of in vitro and in silico toxicity data. Beyond safety, this methodology benefits applications such as discovery of new constituents, identification of active compounds, and untargeted adulteration screening, making it a versatile tool for botanical research and quality assurance. In addition to botanicals, these methods could be used for another classes of substances found at trace amounts like extractable and leachable chemicals [50].

This study’s findings underscore the importance of detailed chemical analysis for the continued development of safety evaluation tools in the botanical field. The multi-detector approach demonstrated here provides a valuable resource for both researchers and regulatory bodies, enabling the rigorous assessment of complex botanical mixtures. Further data on ashwagandha root extract, including HPTLC data supporting material authentication, are available through the Chemical Effects on Biological Systems (CEBS) database, which can serve as an essential repository for future investigations (NIEHS, 2024). Expanding the use of such platforms to other botanicals could pave the way for more consistent and reliable safety evaluations across a wide range of natural products.

## Supplementary Information

Below is the link to the electronic supplementary material.

ESM1 (DOCX 6.25 MB)

## Data Availability

Summary data, including representative chromatograms and compound profiles, are provided in the supplementary files. These materials are also publicly accessible via the National Institute of Environmental Health Sciences (NIEHS) Chemical Effects in Biological Systems (CEBS) database at: https://cebs-ext.niehs.nih.gov/cebs/paper/15717.

## Declarations

### Statement on animal welfare

N/A.

### Source of biological material

N/A.
