# Supplementary material for: A multi-detector analytical approach for characterizing complex botanical extracts: a case study on ashwagandha
Source: Anal Bioanal Chem. 2025 Jul 22;417(21):4895–906. doi: 10.1007/s00216-025-06006-8 (PMC12367915; doi:10.1007/s00216-025-06006-8)
Supplement: Supplementary file 1 — (DOCX 6.25 MB) [file 216_2025_6006_MOESM1_ESM.docx]

**Supplemental Information**

| **** |
| --- |
| **** |
| **** |
| **** |
| **Figure SI 1.** Comparative chromatograms for the four ashwagandha extracts by (in order) PDA, CAD, positive HRMS, and negative HRMS. The colored regions were added to emphasize areas where the chromatogram was magnified |

**Table SI 1.** Response factor (RF) calculations to ensure standards are providing linearity and can support a RRF for semi-quantification of the botanical.

|  | **Withanolide A** | | **Withanoside V** | |
| --- | --- | --- | --- | --- |
|  | **mg of constituent/ g of extract** | **Percentage  Compared to RFF** | **mg of constituent/ g of extract** | **Percentage  Compared to RFF** |
| **RRF (CAD) ^1^** | 1.7 | - | 3.9 | - |
| **RF of Constituent (CAD) ^2^** | 1.6 | 95% | 4.1 | 104% |
| **RF of Constituent (HRMS) ^3^** | 1.9 | 112% | 4.9 | 124% |
| 1. Relative Response Factor (RFF) was 6.2E-1. the average of the seven analytes used for the CAD semi-quantification 2. The RFs by CAD for withanolide A and withanoside V were 6.5E-1 and 6.0E-1, respectively. 3. The RFs by HRMS for withanolide A and withanoside V were 2.8E7 and 1.8E7, respectively. | | | | |

.

**Table SI 2.** Comparison of semi-quantitative estimates for Withanolide A and Withanoside V using different response factors

| Tropine | | | | | |
| --- | --- | --- | --- | --- | --- |
| Conc. (µg/mL) | Area | RF |  | |  |
| 0.8 | 0.36 | 0.45 | Overall Mean | 0.67 |  |
| 4 | 2.6 | 0.65 | SD | 0.16 |  |
| 20 | 16 | 0.79 | RSD (%) | 24 |  |
| 100 | 77 | 0.77 | r^2^ | 0.9999 |  |
| Kaempferol-ß-O-robinoside-7-O-glucoside | | | | | |
| Conc. (µg/mL) | Area | RF |  | |  |
| 0.8 | 0.50 | 0.63 | Overall Mean | 0.55 |  |
| 4.0 | 1.89 | 0.47 | SD | 0.063 |  |
| 20 | 11 | 0.54 | RSD (%) | 12 |  |
| 100 | 57 | 0.57 | r2 | 0.9999 |  |
| Withanolide A | | | | | |
| Conc. (µg/mL) | Area | RF |  | |  |
| 0.8 | 0.49 | 0.61 | Overall Mean | 0.65 |  |
| 4.0 | 2.3 | 0.58 | SD | 0.070 |  |
| 20 | 13 | 0.66 | RSD (%) | 11 |  |
| 100 | 74 | 0.74 | r2 | 0.9996 |  |
| Withanoside IV | | | | | |
| Conc. (µg/mL) | Area | RF |  | |  |
| 0.8 | 0.37 | 0.46 | Overall Mean | 0.59 |  |
| 4.0 | 2.4 | 0.59 | SD | 0.092 |  |
| 20 | 13 | 0.63 | RSD (%) | 16 |  |
| 100 | 67 | 0.67 | r2 | 0.9999 |  |
| ß-Tropine | | | | | |
| Conc. (µg/mL) | Area | RF |  | |  |
| 0.8 | 0.47 | 0.59 | Overall Mean | 0.73 |  |
| 4.0 | 3.1 | 0.77 | SD | 0.010 |  |
| 20 | 16 | 0.81 | RSD (%) | 14 |  |
| 100 | 77 | 0.77 | r2 | 0.9998 |  |
| Withanone | | | | | |
| Conc. (µg/mL) | Area | RF |  | |  |
| 0.8 | 0.35 | 0.44 | Overall Mean | 0.54 |  |
| 4.0 | 2.1 | 0.52 | SD | 0.078 |  |
| 20 | 11 | 0.57 | RSD (%) | 14 |  |
| 100 | 62 | 0.62 | r2 | 0.9998 |  |
| Withanoside V | | | | | |
| Conc. (µg/mL) | Area | RF |  | |  |
| 0.8 | 0.50 | 0.63 | Overall Mean | 0.60 |  |
| 4.0 | 2.0 | 0.51 | SD | 0.066 |  |
| 20 | 12 | 0.60 | RSD (%) | 11 |  |
| 100 | 66 | 0.66 | r2 | 0.9997 |  |
| Average | | | | | |
| Conc. (µg/mL) | Area | RF |  | |  |
| 0.8 | 0.43 | 0.54 | Overall Mean | 0.62 |  |
| 4.0 | 2.3 | 0.58 | SD | 0.066 |  |
| 20 | 13 | 0.66 | RSD (%) | 11 |  |
| 100 | 69 | 0.69 | r2 | 1.000 |  |

**Table SI 3.** Proposed identifications of constituents that were semi-quantified by CAD above an AET of 6.8 µg/mg utilizing the UHPLC-PDA-CAD-HRMS platform for Ashwagandha Root (*Withania sominifera*) extract.

| **CAD**  **Peak**^1^ | **RT**  **(min)**^2^ | **Exp. *m/z***^7^  **+, top**  **-, bottom** | **Mass Acc.**  **+, top**  **-, bottom** | **Proposed ID**  **Molecular Formula**  **(CAS)**  **Confidence**^3^ | **Structure**  **SMILES** | **µg constituent/**  **mg extract** | **Comments** |
| --- | --- | --- | --- | --- | --- | --- | --- |
| 1a | 0.89 | 203.0526 ^5^  365.1054 ^5^  527.1586 ^5^  707.2219 ^5^  215.0328 ^6^  377.0856 ^6^  539.1390 ^6^  719.2019 ^6^ | 0.2  -0.1 | Saccharides  C_6_H_12_O_6_ + (C_6_H_10_O_5_)_x_  Tentative |   OC1C(O)C(OCC2OC(OCC3OC(O)C(O)C(O)C3O)C(O)C(O)C2O)OC(CO)C1O | 110 | HRMS supports molecular formulae.  MS/MS supports structure(s) via mzCloud online database. ^4^  Saccharide-based structures (i.e., sugars) are common to botanicals.  The chirality cannot be determined. |
| 1b |  | 338.1448  - | -0.0  - | Sugar + C_7_H_13_NO_4_  C_13_H_23_NO_9_  Partial |   OC1C(O)C(CO)OC(OC#CC#CC#CC)C1O.O[N+]([O-])=O.[HH].[HH].[HH].[HH] |  | HRMS supports molecular formula.  MS/MS supports structure with loss of sugar, but the aglycone cannot be determined. |
| 2a | 0.96 | 203.0526 ^5^  365.1054 ^5^  527.1586 ^5^  707.2219 ^5^  215.0328 ^6^  377.0856 ^6^  539.1390 ^6^  719.2019 ^6^ | 0.2  -0.1 | Saccharides  C_6_H_12_O_6_ + (C_6_H_10_O_5_)_x_  Tentative |   OC1C(O)C(OCC2OC(OCC3OC(O)C(O)C(O)C3O)C(O)C(O)C2O)OC(CO)C1O | 280 | HRMS supports molecular formulae.  MS/MS supports structure(s) via mzCloud online database. ^4^  Saccharide-based structures (i.e., sugars) are common to botanicals.  The chirality cannot be determined. |
| 3a | 0.99 | 203.0526 ^5^  365.1054 ^5^  527.1586 ^5^  707.2219 ^5^  215.0328 ^6^  377.0856 ^6^  539.1390 ^6^  719.2019 ^6^ | 0.2  -0.1 | Saccharides  C_6_H_12_O_6_ + (C_6_H_10_O_5_)_x_  Tentative |   OC1C(O)C(OCC2OC(OCC3OC(O)C(O)C(O)C3O)C(O)C(O)C2O)OC(CO)C1O | 550 | HRMS supports molecular formulae  MS/MS supports structure(s) via mzCloud online database. ^4^  Saccharide-based structures (i.e., sugars) are common to botanicals.  The chirality cannot be determined. |
| 2/3b | 0.96-  0.99 | 142.1226  - | -0.5  - | Tropine OR β-tropine  C_8_H_15_NO  (120-29-6  135-97-7)  Reference |   CN1C2CCC1CC(O)C2 | 0.97 | HRMS supports molecular formula.  MS/MS supports structure via reference standard.  Retention time matches reference standard.  The combined levels of tropane and β-tropine were similar to what was observed in the USP Ashwagandha reference material (1.5 µg/mg). |
| 4 | 1.59 | 527.1583 ^5^  503.1620 | 0.0  0.5 | Trisaccharide  (e.g., raffinose)  C_18_H_32_O_16_  (e.g., 512-69-6)  Matched |   OCC1OC(OCC2OC(OC3(CO)OC(CO)C(O)C3O)C(O)C(O)C2O)C(O)C(O)C1O | 0.54 ± 0.3 | HRMS supports molecular formula  MS/MS supports structure via mzCloud online database. ^4^  Saccharide-based structures (i.e., sugars) are common to botanicals.  The exact connectivity and chirality cannot be determined. |
| 5 | 2.01 | 130.0498  128.0353 | -0.5  -0.4 | Pyroglutamic acid  C_5_H_7_O_3_N  (98-79-3)  Matched |   O=C1NC(C(O)=O)CC1 | 0.55 ± 0.3 | HRMS supports molecular formula  MS/MS supports structure via mzCloud online database. ^4^  Amino acid-based structures are common to botanicals. |
| 6a | 2.25 | 190.0710  188.0564 | -0.2  -0.9 | Acetylglutamic acid  C_7_H_11_O_5_N  (1188-37-0)  Matched |   OC(CCC(NC(C)=O)C(O)=O)=O | 0.58 ± 0.3 | HRMS supports molecular formula.  MS/MS supports structure via mzCloud online database. ^4^  Amino acid-based structures are common to botanicals.  This is the only major signal that appears in both positive and negative mode. |
| 6b |  | 144.1381  - | -1.5  - | Hygroline  OR  1-α-methyl-Piperidineethanol  C_8_H_17_ON  (496-47-9  OR  934-90-7)  Tentative |   CN1CCCC1CC(C)O    OC(C)CC1CCCCN1 |  | HRMS supports molecular formula.  MS/MS supports structure via manual interpretation.  No literature/database available to match. |
| 7 | 3.42 | 142.1225  - | -0.1  - | Hygrine OR Pelletierine  C_8_H_15_ON  (496-49-1  OR  2858-66-4)  Tentative |   CN1CCCC1CC(C)=O  O=C(C)CC1NCCCC1 | 0.39 ± 0.2 | HRMS supports molecular formula.  MS/MS supports structure via literature {Rubio, 2019 #40} . |
| 8 | 4.28 | 144.1381  - | -1.4  - | Hygroline  OR  Sedridine  C_8_H_17_ON  (496-47-9  OR  501-83-7)  Tentative |   CN1CCCC1CC(C)O  OC(C)CC1NCCCC1 | 0.76 ± 0.4 | HRMS supports molecular formula.  MS/MS supports structure via manual interpretation.  No literature/database available to match. |
| 9 | 7.90 | 184.1331  - | -3.6  - | Acetyltropine  C_10_H_18_O_2_N  (3423-27-6)  Tentative |   CN1C2CCC1CC(OC(C)=O)C2 | 0.56 ± 0.2 | HRMS supports molecular formula.  MS/MS supports structure via manual interpretation.  No literature/database available to match. |
| 10a | 20.29 | 474.2596  472.2454 | -0.6  0.2 | N^1^,N^8^-Bis(dihydrocaffeoyl) spermidine  C_25_H_35_O_6_N_3_  (132194-39-9)  Tentative | OC1=CC(CCC(NCCCNCCCCNC(CCC2=CC(O)=C(O)C=C2)=O)=O)=CC=C1O | 0.34 ± 0.3 | HRMS supports molecular formula.  MS/MS supports structure via literature.  Literature contained fragmentation but was NOT related to *Withania {Dou, 2020 #42}{Du, 2019 #41}*. |
| 10b |  | 636.3127  634.2984 | 0.1  0.4 | Lycibarbarspermidines H or I  C_31_H_45_O_11_N_3_  (1884356-81-3 or  1884356-82-4)  Tentative |   OC1=CC(CCC(NCCCNCCCCNC(CCC2=CC(O)=C(O)C=C2)=O)=O)=CC=C1OC3C(O)C(O)C(O)C(CO)O3 |  | HRMS supports molecular formula.  MS/MS supports structure via literature.  Literature contained fragmentation but was NOT related to *Withania {Du, 2019 #41}{Dou, 2020 #42}*. |
| 11a | 20.41 | 474.2596  472.2454 | -0.6  0.2 | N^1^,N^8^-Bis(dihydrocaffeoyl) spermidine  C_25_H_35_O_6_N_3_  (132194-39-9)  Tentative |   OC1=CC(CCC(NCCCNCCCCNC(CCC2=CC(O)=C(O)C=C2)=O)=O)=CC=C1O | 0.46 ± 0.1 | HRMS supports molecular formula.  MS/MS supports structure via literature.  Literature contained fragmentation but was NOT related to *Withania {Du, 2019 #41}{Dou, 2020 #42}*. |
| 11b |  | 636.3127  634.2984 | 0.1  0.4 | Lycibarbarspermidines H or I  C_31_H_45_O_11_N_3_  (1884356-81-3 or  1884356-82-4)  Tentative |   OC1=CC(CCC(NCCCNCCCCNC(CCC2=CC(O)=C(O)C=C2)=O)=O)=CC=C1OC3C(O)C(O)C(O)C(CO)O3 |  | HRMS supports molecular formula.  MS/MS supports structure via literature.  Literature contained fragmentation but was NOT related to *Withania {Du, 2019 #41}{Dou, 2020 #42}*. |
| 12 | 22.82 | 224.1644  - | -0.4  - | Tropigline  C_13_H_21_O_2_N  (533-08-4)  Tentative |   CN1C2CCC1CC(OC(/C(C)=C/C)=O)C2 | 0.51 ± 0.07 | HRMS supports molecular formula.  MS/MS supports structure via manual interpretation.  No literature/database available to match. |
| 13 | 30.95 | 815.4060  813.3914 | 0.0  -0.1 | Withagenin A Diglucoside  C_40_H_62_O_17_  (1379595-75-1)  Matched |   OC1CC(OC2C(O)C(O)C(O)C(COC3C(O)C(O)C(O)C(CO)O3)O2)CC4(O)C1(C)C5C(C(CCC6C(C)C7OC(C(CO)=C(C)C7)=O)C6(C)CC5)C8C4O8 | 0.37 ± 0.1 | HRMS supports molecular formula.  MS/MS supports structure via literature.  Literature contained fragmentation and was related to *W. somnifera* fruits {Bolleddula, 2012 #43}. |
| 14a | 31.22 | 1107.5214  1105.5068 | -0.4  -0.4 | Withanoside IX  C_52_H_82_O_25_  (500721-40-4)  Matched |   OC1CC(OC2C(O)C(O)C(O)C(COC3C(O)C(O)C(O)C(CO)O3)O2)CC4=CCC(C(CCC5C(C)C6OC(C(COC7C(O)C(O)C(O)C(COC8C(O)C(O)C(O)C(CO)O8)O7)=C(C)C6)=O)C5(C)CC9)C9C41C | 2.0 ± 0.3 | HRMS supports molecular formula.  MS/MS supports structure via literature.  Literature contained fragmentation and was related to *W. somnifera* fruits {Bolleddula, 2012 #43}. |
| 14b |  | 1095.5569  1093.5427 | -1.2  -0.9 | (1α,​3β,​22*R*,​24*Z*)​-​1,​22-​dihydroxyergosta-​5,​24-​diene-​3,​26-​diyl bis[6-​*O*-​β-​D-​glucopyranosyl-β-​D-​Glucopyranoside  C_52_H_86_O_24_  (2411842-88-9)  Tentative | OC(C/C(C)=C(COC1C(O)C(O)C(O)C(COC2C(O)C(O)C(O)C(CO)O2)O1)\C)C(C)C3CCC4C5CC=C6CC(OC7C(O)C(O)C(O)C(COC8C(O)C(O)C(O)C(CO)O8)O7)CC(O)C6(C)C5CCC43C |  | HRMS supports molecular formula.  MS/MS supports structure via literature.  Literature only contained HRMS and NMR (i.e., no fragmentation), but was related to *W. somnifera* seeds {Iguchi, 2021 #44}. |
| 15a | 32.38 | 933.5050  931.4907 | -0.4 | C_46_H_76_O_19_  Tentative |   OC(C/C(C)=C(C)/CO)C(C)C1CCC2C3CC=C4CC(O)CC(O)C4(C)C3CCC21C.OC5C(CO)OC(O)C(O)C5O.OC6C(CO)OC(O)C(O)C6O.OC7C(CO)OC(O)C(O)C7O | 0.35 ± 0.06 | HRMS supports molecular formula.  MS/MS supports structure via manual interpretation.  Literature only contained closely related analogues and was related to *W. somnifera* seeds {Iguchi, 2021 #44}. |
| 15b |  | 945.4682  989.4596 ^7^ | -0.8 | Sominone Triglucoside  (Withanoside VIII)  C_46_H_72_O_20_  (519186-57-3)  Matched |   OC(C1)CC(O)C2(C)C1=CCC3C2CCC4(C)C3CCC4C(C)C5OC(C(CO)=C(C)C5)=O.OC6C(CO)OC(O)C(O)C6O.OC7C(CO)OC(O)C(O)C7O.OC8C(CO)OC(O)C(O)C8O |  | HRMS supports molecular formula.  MS/MS supports structure via literature.  Literature contained fragmentation and was related to *W. somnifera* fruits {Bolleddula, 2012 #43} and roots {Zhao, 2002 #45}. |
| 16a | 32.67 | 653.3533  697.3440 ^7^ | 0.2  -0.1 | Withanoside III  C_34_H_52_O_12_  (362472-80-8)  Matched | OC1CC(OC2C(O)C(O)C(O)C(CO)O2)CC3(O)C1(C)C4C(C(CCC5C(C)C6OC(C(CO)=C(C)C6)=O)C5(C)CC4)C7C3O7 | 0.39 ± 0.06 | HRMS supports molecular formula.  MS/MS supports structure via literature.  Literature contained fragmentation and was related to *W. somnifera* fruits {Bolleddula, 2012 #43}. |
| 16b |  | 1241.6175  1239.6013 | 1.2  -0.8 | C_58_H_96_O_28_  Partial |   C/C(CO)=C(C)\CCC(C)C1CCC2C3CC=C4CC(O)CC(O)C4(C)C3CCC21C.OC5C(CO)OC(O)C(O)C5O.OC6C(CO)OC(O)C(O)C6O.OC7C(CO)OC(O)C(O)C7O.OC8C(CO)OC(O)C(O)C8O.OC9C(CO)OC(O)C(O)C9O |  | HRMS supports molecular formula.  MS/MS largely contains losses of sugar moieties. There are several, albeit structurally similar, compounds in literature that would be supported by the HRMS and MS/MS.  None of these options have been reported to come from Withania, but there is high confidence that it is some sterol with four sugar moieties attached. The backbone of the sterol proposed is consistent with those reported in literature.  Molecular formula and MS/MS matches peak 23c with an additional sugar moieties. |
| 17a | 33.07 | 945.4682  943.4540 | -0.8  -0.4 | Sominone Triglucoside  (Withanoside VIII)  C_46_H_72_O_20_  (500722-19-0)  Matched |   OC(C1)CC(O)C2(C)C1=CCC3C2CCC4(C)C3CCC4C(C)C5OC(C(CO)=C(C)C5)=O.OC6C(CO)OC(O)C(O)C6O.OC7C(CO)OC(O)C(O)C7O.OC8C(CO)OC(O)C(O)C8O | 1.3 ± 0.3 | HRMS supports molecular formula.  MS/MS supports structure via literature.  Literature contained fragmentation and was related to *W. somnifera* fruits {Bolleddula, 2012 #43} and roots {Zhao, 2002 #45}. |
| 17b |  | 1079.5632  1077.5485 | -0.1  0.9 | Sterol with four sugar moieties  C_52_H_86_O_23_  Partial |   OC(C1)CCC2(C)C1=CCC3C2CCC4(C)C3CC5C4C(C)C(OC)(CCC(C)CO)O5.OC6C(CO)OC(O)C(O)C6O.OC7C(CO)OC(O)C(O)C7O.OC8C(CO)OC(O)C(O)C8O.OC9C(CO)OC(O)C(O)C9O |  | HRMS supports molecular formula.  MS/MS largely contains losses of sugar moieties. There are several, albeit structurally similar, compounds in literature that would be supported by the HRMS and MS/MS.  None of these options have been reported to come from Withania, but there is high confidence that it is some sterol with four sugar moieties attached. The backbone of the sterol proposed is consistent with those reported in literature.  Fragmentation is similar to the isomer at Peak 20. |
| 18 | 33.17 | 933.5050  931.4902 | -0.4  -0.6 | Sterol with three sugar moieties  C_46_H_76_O_19_  Partial |   CC1(C(O)CCC2)C2=CCC3C1CCC4(C)C3CC5C4C(C)C(OC)(CCC(C)CO)O5.OC6C(CO)OC(O)C(O)C6O.OC7C(CO)OC(O)C(O)C7O.OC8C(CO)OC(O)C(O)C8O | 0.42 ± 0.1 | HRMS supports molecular formula.  MS/MS largely contains losses of sugar moieties. There are several, albeit structurally similar, compounds in literature that would be supported by the HRMS and MS/MS.  None of these options have been reported to come from Withania, but there is high confidence that it is some sterol with four sugar moieties attached. The backbone of the sterol proposed is consistent with those reported in literature.  Molecular formula and MS/MS matches peak 23c with an additional sugar moiety. |
| 19 | 33.70 | 964.4191  962.4050 | -0.9  -0.4 | Lyciumin C  C_49_H_57_O_12_N_9_  (150394-23-3) Tentative |   O=C1NC(C(N2C(C(NC(C(NC3N4C=C(CC(C(O)=O)NC(C(NC(C(NC(C(C(C)C)NC3=O)=O)CC5=CC=CC=C5)=O)CO)=O)C6=C4C=CC=C6)=O)CC7=CC=C(O)C=C7)=O)CCC2)=O)CC1 | 0.49 ± 0.08 | HRMS supports molecular formula.  MS/MS supports structure via literature.  Literature contained fragmentation but was NOT related to *Withania {Li, 2015 #46}*. |
| 20 | 33.96 | 1079.5629  1077.5483 | -0.4  0.6 | C_52_H_86_O_23_  Tentative |   OC(C1)CCC2(C)C1=CCC3C2CCC4(C)C3CC5C4C(C)C(OC)(CCC(C)CO)O5.OC6C(CO)OC(O)C(O)C6O.OC7C(CO)OC(O)C(O)C7O.OC8C(CO)OC(O)C(O)C8O.OC9C(CO)OC(O)C(O)C9O | 0.78 ± 0.2 | HRMS supports molecular formula.  MS/MS largely contains losses of sugar moieties. There are several, albeit structurally similar, compounds in literature that would be supported by the HRMS and MS/MS.  None of these options have been reported to come from Withania, but there is high confidence that it is some sterol with four sugar moieties attached. The backbone of the sterol proposed is consistent with those reported in literature.  Fragmentation is similar to the isomer at Peak 17b. |
| 21 | 34.14 | 569.2412  567.2269 | -0.5  -0.2 | 2,3-Dihydro-3-β-O-sulfate withaferin A  C_28_H_40_O_10_S  (1159096-16-8)  Matched |   O=C1CC(OS(O)(=O)=O)C(O)C23C1(C)C4C(C(CCC5C(C)C6OC(C(CO)=C(C)C6)=O)C5(C)CC4)CC2O3 | 0.42 ± 0.1 | HRMS supports molecular formula.  MS/MS supports structure via literature.  Literature only contained HRMS and NMR (i.e., no fragmentation), but was related to *W. somnifera* leaves {Misra, 2005 #47} and roots {Trivedi, 2017 #48}.  The molecular formula matches withaferin A with a sulfate. |
| 22 | 34.30 | 783.4158  827.4069 ^7^ | -0.4  - | Withanoside IV isomer  C_40_H_62_O_15_  Matched |   OC(C1)CC(O)C2(C)C1=CCC3C2CCC4(C)C3CCC4C(C)C5OC(C(CO)=C(C)C5)=O.OC6C(CO)OC(O)C(O)C6O.OC7C(CO)OC(O)C(O)C7O | 0.47 ± 0.1 | HRMS supports molecular formula.  MS/MS supports structure via literature (isomer of Withanoside IV).  Literature contained fragmentation of Withanoside IV only (not other isomers) and was related to *W. somnifera* fruits {Bolleddula, 2012 #43. |
| 23a | 34.53 | 569.2412  567.2269 | -0.5  -0.2 | 2,3-Dihydro-3-β-O-sulfate withaferin A  C_28_H_40_O_10_S  (871949-35-8)  Matched |   O=C1CC(OS(O)(=O)=O)CC2(O)C1(C)C3C(C(CCC4(O)C(C)C5OC(C(C)=C(C)C5)=O)C4(C)CC3)C6C2O6 | 0.67 ± 0.4 | HRMS supports molecular formula.  Literature only contained HRMS and NMR (i.e., no fragmentation), but was related to *W. somnifera* leaves {Misra, 2005 #47} and roots {Trivedi, 2017 #48}.  MS/MS is similar, though slightly different, than Peak 25.  The molecular formula matches withaferin A with a sulfate. |
| 23b |  | 553.2466  551.2325 | 0.1  0.7 | 2,3-Dihydro-3-β-O-sulfate withanolide B  C_28_H_40_O_9_S  Partial |   O=C1CC(OS(O)(=O)=O)CC2(O)C1(C)C3C(C(CCC4C(C)C5OC(C(C)=C(C)C5)=O)C4(C)CC3)C6C2O6 |  | HRMS supports molecular formula.  MS/MS is similar to the constituent at peak 25 (less one oxygen) but there is no literature for this structure (nor anything with this formula).  The molecular formula matches withanolide B with a sulfate. |
| 23c |  | 771.4524  769.4382 | -0.2  -0.2 | (1α,3β,22*R*,24*Z*)-1,3,22-trihydroxy ergosta-5,24-dien-26-yl 6-*O*-β-D-gluco pyranosyl-β-D-glucopyranoside  C_40_H_66_O_14_  (2411842-87-8)  Tentative |   OC(C1)CC(O)C2(C)C1=CCC3C2CCC4(C)C3CCC4C(C)C(O)C/C(C)=C(C)\CO.OC5C(CO)OC(O)C(O)C5O.OC6C(CO)OC(O)C(O)C6O |  | HRMS supports molecular formula.  Literature only contained HRMS and NMR (i.e., no fragmentation), but was related to *W. somnifera* seeds {Iguchi, 2021 #44}.  Molecular formula and MS/MS matches several other constituents in this sample with varying number of additional sugar moieties. |
| 24 | 34.65 | 897.3877  895.3741 | -3.0  -1.9 | C_51_H_60_O_12_S  Partial |   O=C1CC(OS(=O)(O)=O)C(O)C23C1(C)C4C(C(CCC5C(C)C6OC(C(CO)=C(C)C6)=O)C5(C)CC4)CC2O3.C#CC#CC#CC#CC#CC#CC#CC#CC#CC#CC#CC.O=O.[HH].[HH].[HH].[HH].[HH].[HH].[HH].[HH] | 1.1 ± 0.2 | HRMS supports molecular formula.  In-source fragments and MS/MS support it’s related to peaks 23 and 25 with an addition of C_23_H_20_O_2_. The structure of this addition is unknown.  MS/MS is similar, though slightly different, than Peak 23a. |
| 25 | 35.46 | 569.2412  567.2269 | -0.5  -0.2 | 2,3-Dihydro-3-β-O-sulfate withaferin A  C_28_H_40_O_10_S  (1159096-16-8)  Tentative |   O=C1CC(OS(=O)(O)=O)C(O)C23C1(C)C4C(C(CCC5C(C)C6OC(C(CO)=C(C)C6)=O)C5(C)CC4)CC2O3 | 0.38 ± 0.1 | HRMS supports molecular formula.  MS/MS supports structure via literature.  Literature only contained HRMS and NMR (i.e., no fragmentation), but was related to *W. somnifera* leaves {Misra, 2005 #47} and roots {Trivedi, 2017 #48}.  MS/MS is similar, though not exact, to Peak 23a.  The molecular formula matches withaferin A with a sulfate. |
| 26a | 37.15 | 783.4155  827.4065 ^7^ | -0.9  -0.7 | Withanoside IV  C_40_H_62_O_15_  (362472-81-9)  Reference |   OC1CC(OC2C(O)C(O)C(O)C(COC3C(O)C(O)C(O)C(CO)O3)O2)CC4=CCC(C(CCC5C(C)C6OC(C(CO)=C(C)C6)=O)C5(C)CC7)C7C41C | 6.8 | HRMS supports molecular formula.  MS/MS supports structure via reference standard.  Retention time matches the reference standard. |
| 26b |  | 489.2853  533.2760 ^7^ | -0.0  0.7 | Viscosalactone B or related isomer  C_28_H_40_O_7_  (76938-46-0)  Matched |   O=C1CC(O)C(O)C23C1(C)C4C(C(CCC5C(C)C6OC(C(CO)=C(C)C6)=O)C5(C)CC4)CC2O3 |  | HRMS supports molecular formula.  MS/MS supports structure via literature.  Literature contained fragmentation of Viscosalactone B only (not other isomers) and was related to *W. somnifera* fruits {Bolleddula, 2012 #43}.  Retention time is shifted and likely not a major contributor to mass. |
| 27 | 37.50 | 489.2845  533.2760 ^7^ | -0.4  0.7 | Viscosalactone B or related isomer  C_28_H_40_O_7_  (76938-46-0)  Matched |   O=C1CC(O)C(O)C23C1(C)C4C(C(CCC5C(C)C6OC(C(CO)=C(C)C6)=O)C5(C)CC4)CC2O3 | 0.43 ± 0.08 | HRMS supports molecular formula.  MS/MS supports structure via literature.  Literature contained fragmentation of Viscosalactone B only (not other isomers) and was related to *W. somnifera* fruits {Bolleddula, 2012 #43}. |
| 28 | 37.76 | 489.2845  533.2760 ^7^ | -0.4  0.7 | Viscosalactone B or related isomer  C_28_H_40_O_7_  (76938-46-0)  Matched |   O=C1CC(O)C(O)C23C1(C)C4C(C(CCC5C(C)C6OC(C(CO)=C(C)C6)=O)C5(C)CC4)CC2O3 | 0.48 ± 0.1 | HRMS supports molecular formula.  MS/MS supports structure via literature.  Literature contained fragmentation of Viscosalactone B only (not other isomers) and was related to *W. somnifera* fruits {Bolleddula, 2012 #43}. |
| 29 | 38.46 | 783.4155  827.4062 ^7^ | -0.9 | Withanoside VI  C_40_H_62_O_15_  (362472-81-9)  Matched |   OC1CC(OC2C(O)C(O)C(O)C(COC3C(O)C(O)C(O)C(CO)O3)O2)CC4=CCC(C(CCC5C(C)(O)C6OC(C(C)=C(C)C6)=O)C5(C)CC7)C7C41C | 2.5 ± 0.5 | HRMS supports molecular formula.  MS/MS supports structure via literature (isomer of Withanoside IV).  Literature contained fragmentation of Withanoside IV only (not other isomers) and was related to *W. somnifera* fruits {Bolleddula, 2012 #43} . |
| 30 | 38.99 | 799.4110  797.3962 | -0.1  -0.4 | Withanoside II  C_40_H_62_O_16_  (362472-79-5)  Matched |   O=C1OC(CC(=C1C)C)C(C)C2CCC3C4C5OC5C6(O)CC(OC7OC(COC8OC(CO)C(O)C(O)C8O)C(O)C(O)C7O)CC(O)C6(C)C4CCC23C | 2.6 ± 0.5 | HRMS supports molecular formula.  MS/MS supports structure via literature.  Literature contained fragmentation and was related to *W. somnifera* fruits {Bolleddula, 2012 #43}. |
| 31a | 40.14 | 473.2897  517.2813 ^7^ | -0.1  0.7 | 23, 24 Dihydrowithaferin A  C_28_H_40_O_6_  (5589-41-3)  Matched |   OC1C=CC(O)C23C1(C)C4C(C(CCC5C(C)C6OC(C(CO)=C(C)C6)=O)C5(C)CC4)CC2O3 | 0.35 ± 0.1 | HRMS supports molecular formula.  MS/MS supports structure via literature.  Literature contained fragmentation and was related to *W. somnifera* fruits {Bolleddula, 2012 #43}. |
| 31b |  | 351.2143 ^5^  327.2175 | 0.0  -0.5 | 9,12,13-Trihydroxy-10,15-octadecadienoic acid  C_18_H_32_O_5_  (51146-90-8)  Tentative |   OC(CCCCCCCC(O)/C=C/C(O)C(O)C/C=C\CC)=O |  | HRMS supports molecular formula.  MS/MS supports structure via literature.  Literature does not contain references to *W. somnifera {Cong, 2021 #49}.* |
| 31c |  | 823.4975 ^5^  799.5003 | 0.0  0.2 | Unidentified  (Potential Artifact)  C_46_H_72_O_11_  Partial |  |  | HRMS supports molecular formula.  Interestingly, MS/MS fragments includes 31a (M+Na]^+^) and 31b ([M+H]^+^). Further investigation to determine if this is an in-source product or if the constituent is those two constituents connected. |
| 32 | 40.33 | 976.4554  974.4415 | -0.7  -0.9 | C_51_H_61_O_11_N_9_  Partial |  | 0.26 ± 0.08 | HRMS supports molecular formula.  Isotope ratios support molecular formula. |
| 33 | 40.49 | 621.3634  665.3540 ^7^ | 0.1  -0.4 | Coagulin Q  C_34_H_52_O_10_  (261637-26-7)  Matched |   OC1CC(OC2C(O)C(O)C(O)C(CO)O2)C=C3C1(C)C4C(C(CCC5C(C)(O)C6OC(C(C)=C(C)C6)=O)C5(C)CC4)CC3 | 0.55 ± 0.2 | HRMS supports molecular formula  MS/MS supports structure via literature.  Literature contained fragmentation and was related to *W. somnifera* fruits {Bolleddula, 2012 #43}. |
| 34 | 41.38 | 637.3585  681.3489 ^7^ | 0.4  -0.4 | Withanoside I  C_34_H_52_O_11_  (362472-78-4)  Matched |   OC1CC(OC2C(O)C(O)C(O)C(CO)O2)CC3(O)C1(C)C4C(C(CCC5C(C)(O)C6OC(C(C)=C(C)C6)=O)C5(C)CC4)C7C3O7 | 0.32 ± 0.04 | HRMS supports molecular formula  MS/MS supports structure via literature.*  Literature contained fragmentation and was related to *W. somnifera* fruits {Bolleddula, 2012 #43}.  *Fragmentation was on [M+H-H_2_O]^+^ due to low abundance of [M+H]^+^. |
| 35 | 42.95 | 471.2740  515.2653 ^7^ | -0.2  0.5 | Withaferin A  C_28_H_38_O_6_  (5119-48-2)  Reference |   O=C1C=CC(O)C23C1(C)C4C(C(CCC5C(C)C6OC(C(CO)=C(C)C6)=O)C5(C)CC4)CC2O3 | 5.2 ± 0.8 | HRMS supports molecular formula  MS/MS supports structure via reference standard.  Retention time matches reference standard. |
| 36 | 43.31 | 353.2298 ^5^  329.2332 | -0.2  -0.4 | 9,10,13-trihydroxy octadecenoic acid  C_18_H_34_O_5_  (29907-56-0, 61911-67-9, etc.)  Tentative |   OC(CCCCCCCC(O)Ccc(O)c(O)cccCC)=O | 0.63 ± 0.1 | HRMS supports molecular formula  MS/MS supports structure via mzCloud^4^ and literature, but relative intensities differ. Placement of double bond cannot be confirmed but matches to literature/database were highlighted {Liang, 2018 #50}. |
| 37 | 43.44 | 505.2616  503.2476 | -0.5  0.6 | Sulfonated  Withaferin A  C_28_H_40_O_6_S  (2354327-03-8  OR  2354326-93-3)  Tentative |   O=C1CC(S)C(O)C23C1(C)C4C(C(CCC5C(C)C6OC(C(CO)=C(C)C6)=O)C5(C)CC4)CC2O3  O=C1CC2C(O)C3(O)C1(C)C4C(C(CCC5C(C)C6OC(C(CO)=C(C)C6)=O)C5(C)CC4)CC3S2 | 0.31 ± 0.04 | HRMS supports molecular formula.  MS/MS supports structure via manual interpretation.  No literature/database fragmentation available to match. Constituent only reported as synthetic product of withaferin A {Casero, 2017 #51}. |
| 38 | 43.92 | 471.2740  515.2653 ^7^ | -0.2  0.5 | Withanone isomer  (Withanolide D)  C_28_H_38_O_6_  (30655-48-2)  Tentative |   O=C1C=CCC2(O)C1(C)C3C(C(CCC4(O)C(C)C5OC(C(C)=C(C)C5)=O)C4(C)CC3)C6C2O6 | 1.4 ± 0.2 | HRMS supports molecular formula  MS/MS is similar to other isomers.  Retention time does not match reference standard for withanone but matches with an impurity in the withanone standard. Can’t determine which isomer without standard.  Withanolide D is a possibility based on it’s reported abundance in literature {Ganzera, 2003 #52}. |
| 39 | 44.26 | 507.2506  505.2365 | -0.3  0.5 | 6α-Chloro-5β-hydroxywithaferin A  C_28_H_39_O_6_Cl  52329-20-1  Tentative |   O=C1C=CC(O)C2(O)C1(C)C3C(C(CCC4C(C)C5OC(C(CO)=C(C)C5)=O)C4(C)CC3)CC2Cl | 1.1 ± 0.2 | HRMS supports molecular formula {Tong, 2011 #53}.  MS/MS supports structure via manual interpretation.  No literature/database fragmentation available to match. |
| 40 | 45.07 | 471.2740  515.2653 ^7^ | -0.2  0.5 | Withanolide A  C_28_H_38_O_6_  (32911-62-9)  Reference |   O=C1C=CCC2(O)C1(C)C3C(C(CCC4C(O)(C)C5OC(C(C)=C(C)C5)=O)C4(C)CC3)C6C2O6 | 2.1 ± 0.4 | HRMS supports molecular formula  MS/MS supports structure via reference standard.  Retention time matches reference standard. |
| 41 | 45.62 | 767.4203  765.4062 | -1.2  -0.7 | Withanoside V  C_40_H_62_O_14_  (256520-90-8)  Reference |   OC1CC(OC2C(O)C(O)C(O)C(COC3C(O)C(O)C(O)C(CO)O3)O2)CC4=CCC(C(CCC5C(C)C6OC(C(C)=C(C)C6)=O)C5(C)CC7)C7C41C | 3.9 | HRMS supports molecular formula  MS/MS supports structure via reference standard.  Retention time matches reference standard. |
| 42 | 47.18 | 517.3157  561.3069 ^7^ | -0.5  0.0 | (20S,22R)-3-acetate δ-lactone 6α,7α-epoxy-1α,3β,5,22-tetrahydroxy-5α-Ergost-24-en-26-oic acid  C_30_H_44_O_7_  (33903-25-2)  Tentative |   OC1CC(OC(C)=O)CC2(O)C1(C)C3C(C(CCC4C(C)C5OC(C(C)=C(C)C5)=O)C4(C)CC3)C6C2O6 | 0.33 ± 0.05 | HRMS supports molecular formula  MS/MS supports structure via manual interpretation.  Literature only makes mention that this is a known constituent of *W. somnifera {Kirson, 1971 #54}*. |
| 43a | 48.39 | 605.3684  649.3591 ^7^ | 0.0  -0.4 | (1α,​3β,​22R)​-δ-​lactone 3-​(β-​D-​glucopyranosyloxy)​-​1,​22-​dihydroxy Ergosta-​5,​24-​dien-​26-​oic acid  C_34_H_52_O_9_  (1567812-88-7)  Tentative |   OC1CC(OC2C(O)C(O)C(O)C(CO)O2)CC3=CCC(C(CCC4C(C)C5OC(C(C)=C(C)C5)=O)C4(C)CC6)C6C31C | 0.33 ± 0.04 | HRMS supports molecular formula  MS/MS supports structure via manual interpretation.  Literature only makes mention that this is a known constituent of *W. somnifera* seeds {Iguchi, 2021 #44}. |
| 43b |  | 537.2341  535.2199 | 0.4  1.0 | (3α,​4β,​5β,​6α,​22*R*)​- δ-​lactone 3,​6-​epidithio-​4,​5,​22,​27-​tetrahydroxy-​1-​oxo- Ergost-​24-​en-​26-​oic acid  C_28_H_40_O_6_S_2_  (2354326-97-7)  Partial |   O=C1CC2C(O)C3(O)C1(C)C4C(C(CCC5C(C)C6OC(C(CO)=C(C)C6)=O)C5(C)CC4)CC3SS2 |  | HRMS supports molecular formula.  MS/MS supports structure via manual interpretation.  No literature/database fragmentation available to match. Constituent only reported as synthetic product of withaferin A {Casero, 2017 #51}. |
| 44 | 52.21 | 975.5280  973.5132 | -0.7  -0.9 | Ashwagandhanolide  C_56_H_78_O_12_S  (919478-81-2)  Matched |   O=C1C=CC(O)C2(O)C1(C)C3C(C(CCC4C(C)C5OC(C(CO)=C(C)C5)=O)C4(C)CC3)CC2SC6C(C7(C)C8C(C(CCC9C(C)C%10OC(C(CO)=C(C)C%10)=O)C9(C)CC8)C6)(O)C(O)C=CC7=O | 0.43 | HRMS supports molecular formula  MS/MS supports structure via manual interpretation.  Literature only contained HRMS and NMR (i.e., no fragmentation), but was related to *W. somnifera* roots {Subbaraju, 2006 #55}. |
| 45 | 54.18 | 455.2791  - | -0.2  - | Withanolide B  C_28_H_38_O_5_  (56973-41-2)  Reference |   O=C1C=CCC2(O)C1(C)C3C(C(CCC4C(C)C5OC(C(C)=C(C)C5)=O)C4(C)CC3)C6C2O6 | 0.61 | HRMS supports molecular formula  MS/MS supports structure via reference standard.  Retention time matches reference standard. |
| 46a | 76.77 | 256.2638  - | 0.2  - | Hexadecanamide  C_16_H_33_NO  (629-54-9)  Matched |   NC(CCCCCCCCCCCCCCC)=O | 0.40 | HRMS supports molecular formula  MS/MS supports structure via mzCloud online database. ^4^  Alkylamides are common to botanicals. |
| 46b |  | 319.1946 ^8^  279.2330 | 0.4  0.0 | Linoleic acid  C_18_H_32_O_2_  (60-33-3)  Tentative |   OC(CCCCCCC/C=C\C/C=C\CCCCC)=O |  | HRMS supports molecular formula  MS/MS supports structure via mzCloud online database. ^4^  Fatty acids are common to botanicals. |
| 1. P&G CAD peak numbers reflect the sequential order of peaks detected and integrated in the UHPLC-CAD chromatogram of the extracted sample. If multiple components (determined from MS data) eluted within the integration window of the CAD peak, sequential letter designations were appended to the peak number. 2. Retention times listed are based on the time the constituent(s) was detected by the CAD detector. The retention times for the HRMS were approximately 0.1 min later. 3. Confidence level assignments from lowest confidence to highest confidence based on TAC Standard Laboratory Practice SLP-MVSA-1010:    1. **Partial** molecular formula derived from exact mass measurements    2. **Tentative** molecular formula derived from exact mass measurements and proposed structure supported by either MS/MS spectrum or UV spectrum or literature data reporting the structure in plants of the same genus (*Withania*) as *Withania somnifera*.    3. **Matched** molecular formula derived from exact mass measurements and proposed structure supported by MS/MS (manual interpretation) and literature data reporting the structure in *Withania somnifera* or proposed structure supported by MS/MS (match with database) and compound reasonably likely to be present in plant material.    4. **Reference** same criteria as a “Matched” identification and there is a retention time and MS/MS match to an authentic reference standard. 4. Databases for reference:    1. **mzCloud**: An online (https://www.mzcloud.org) curated mass spectral database containing high resolution/accurate mass fragmentation spectra. 5. Sodiated adduct was measured (i.e., [M+Na]^+^). 6. Chlorinated adduct was measured (i.e., [M+Cl]^-^). 7. Formate adduct was measured (i.e., [M-H+CH_2_O_2_]^-^). 8. Calcium adduct was measured (i.e., [M-H+Ca]^+^). | | | | | | | |

**Table SI 3.** Extracted ion, CAD, and total ion chromatograms of each peak and their corresponding MS and MS/MS fingerprint as described in **Table SI 2**.

| 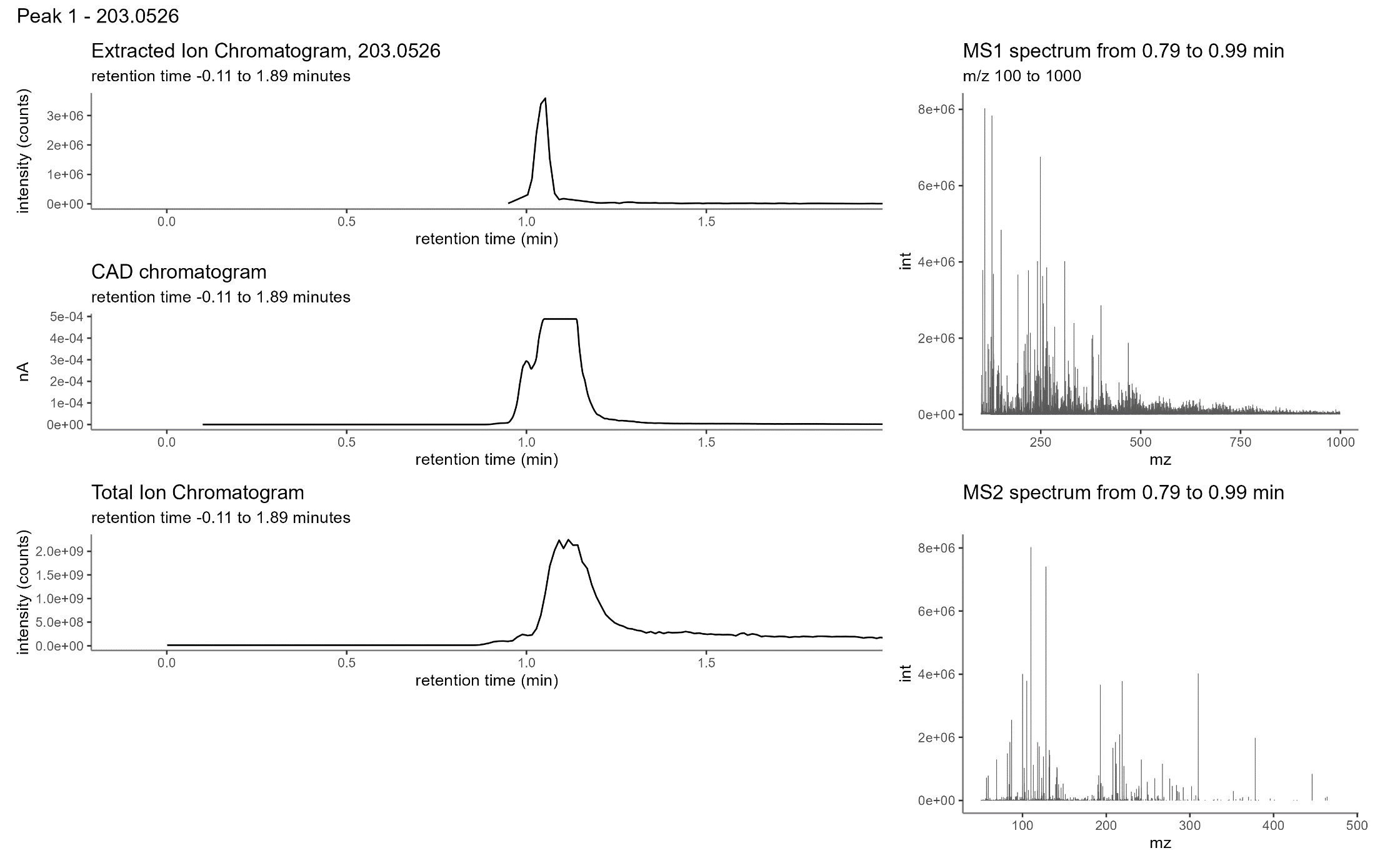 |
| --- |
| 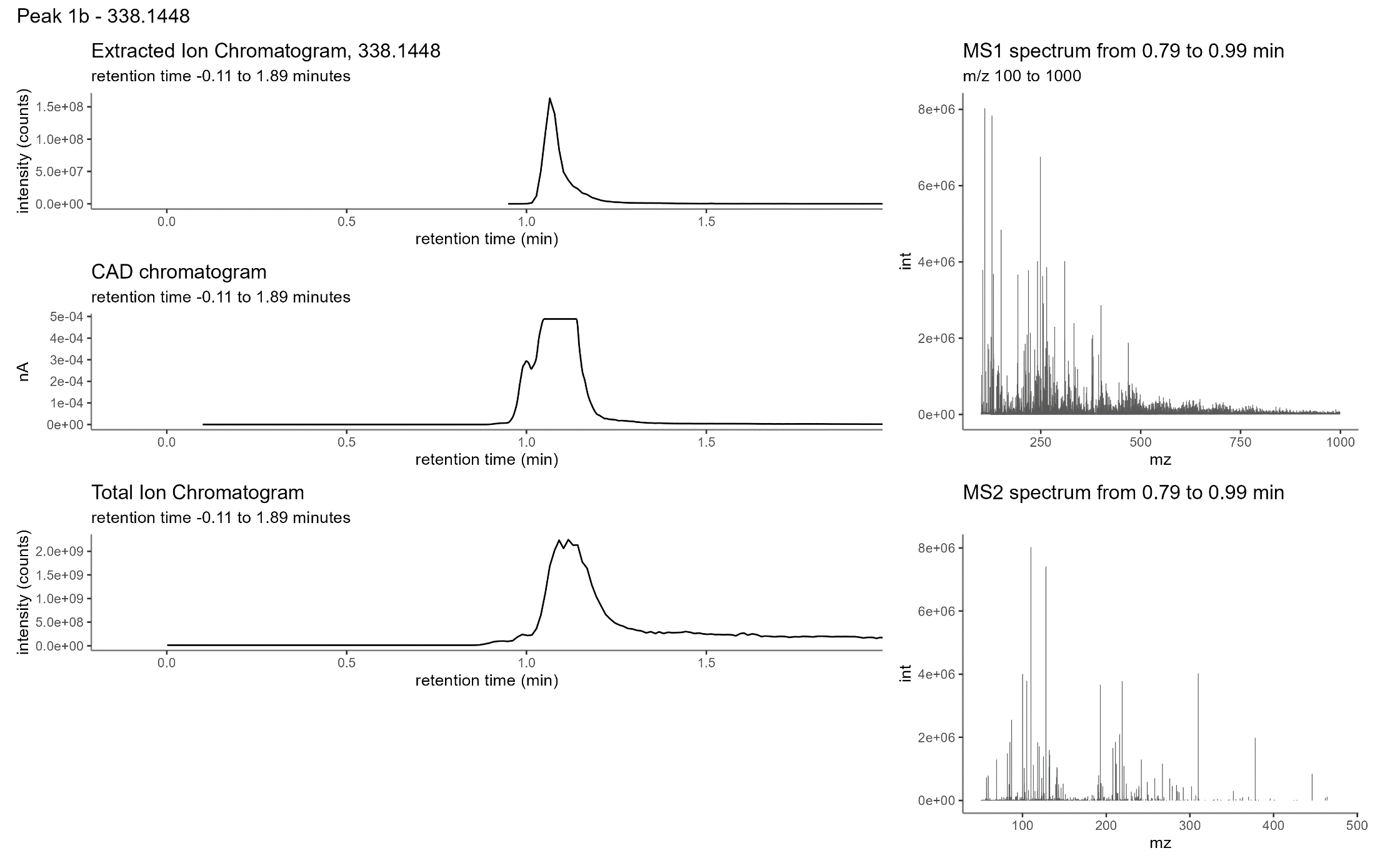 |
| 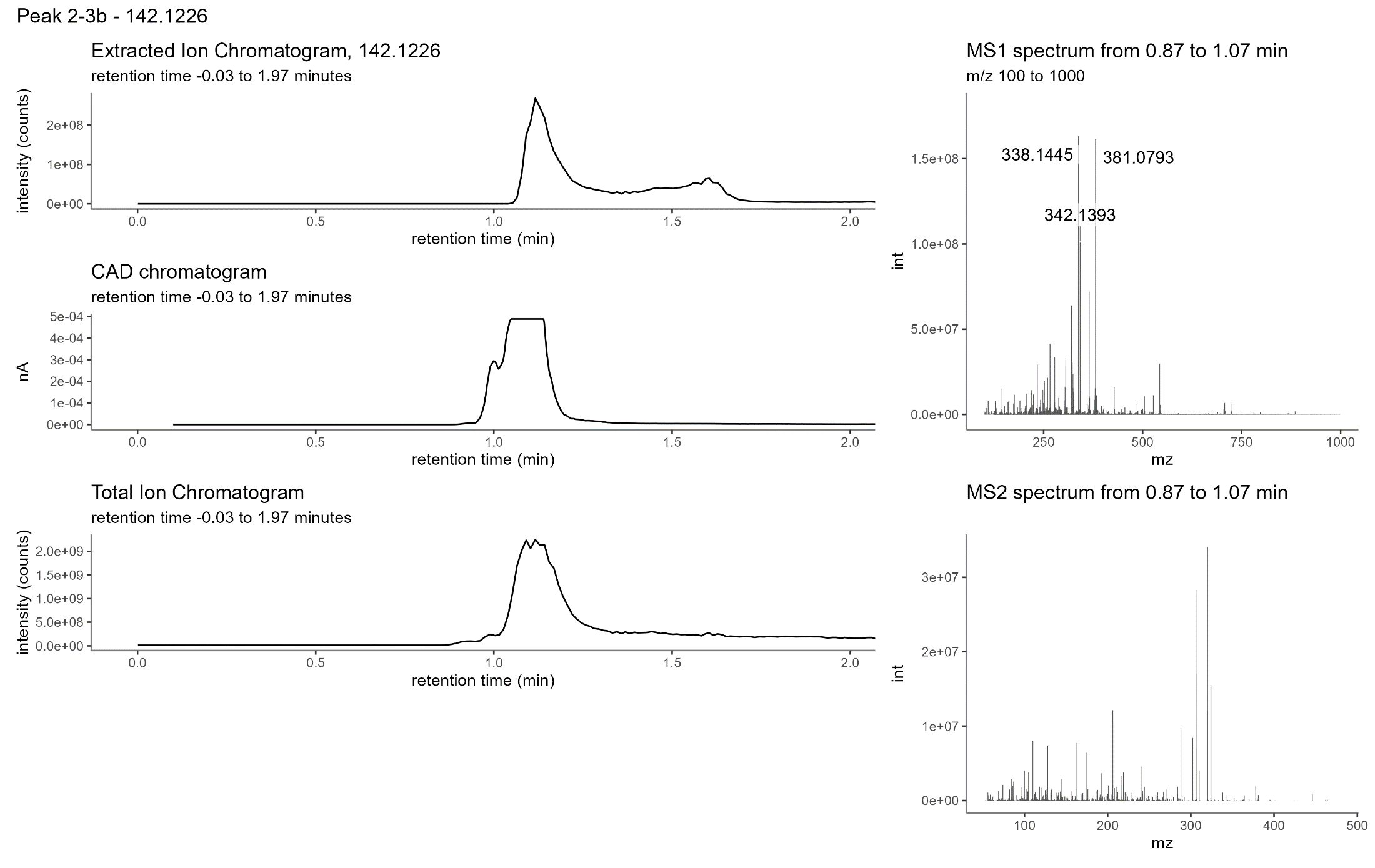 |
| 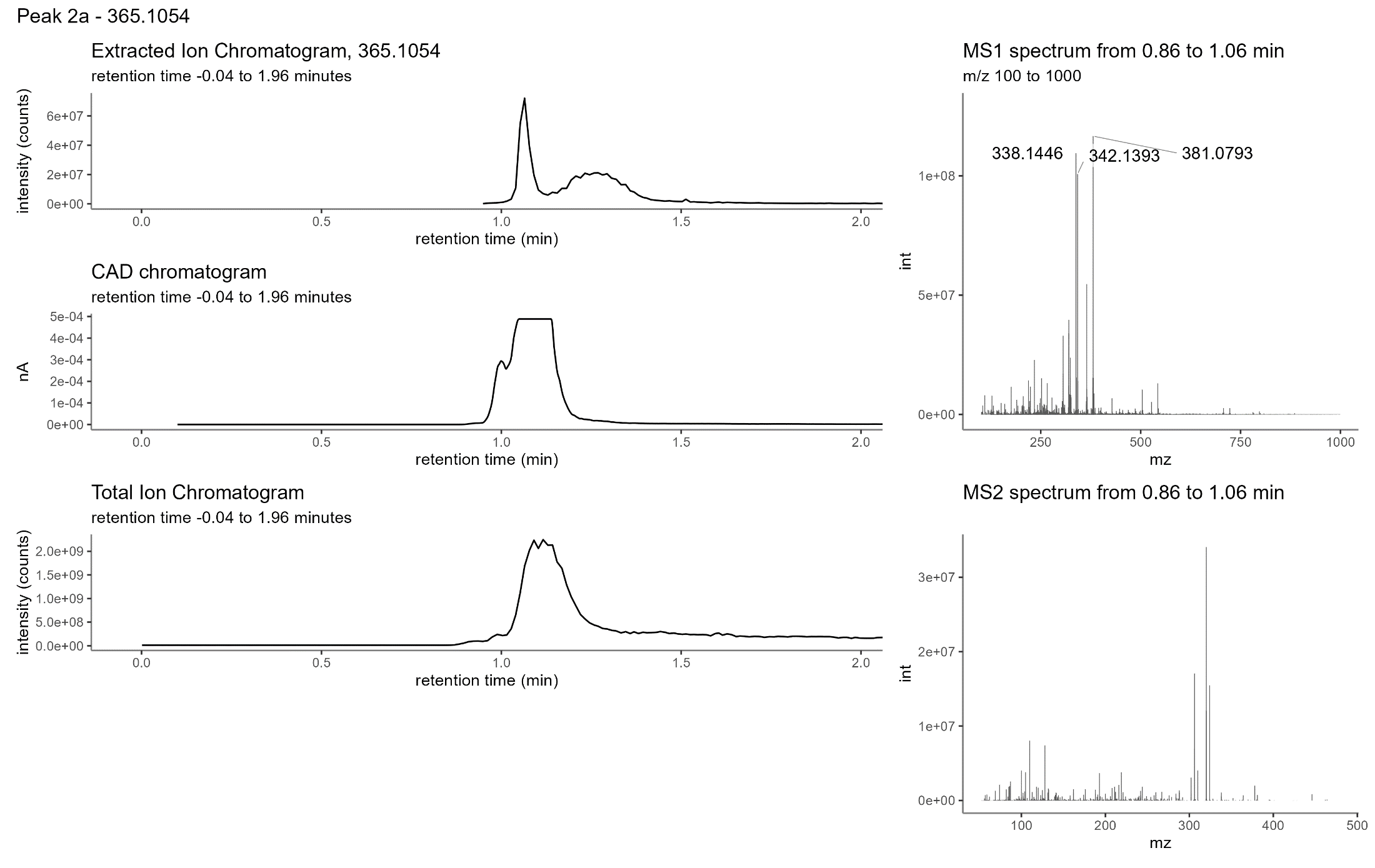 |
| 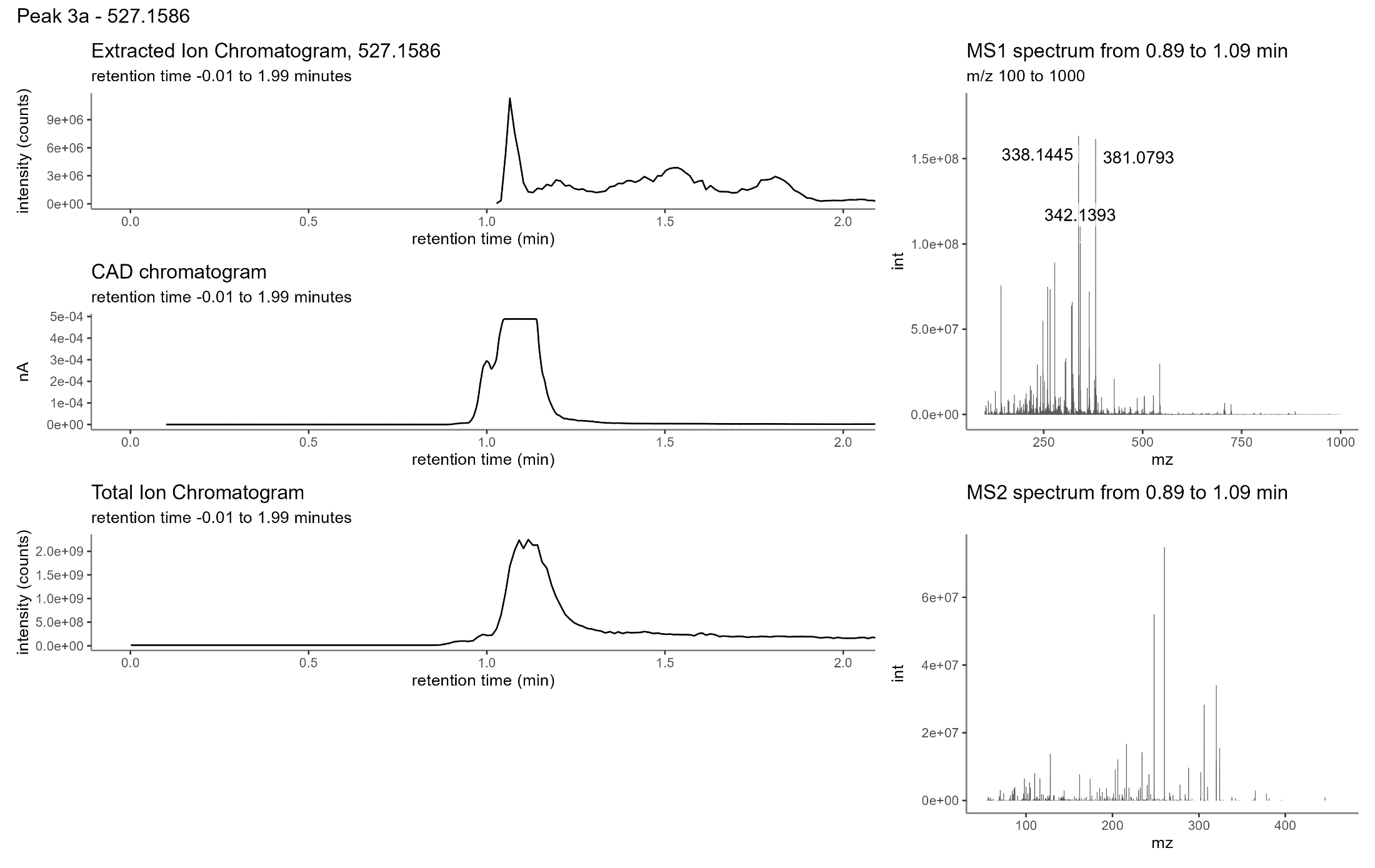 |
| 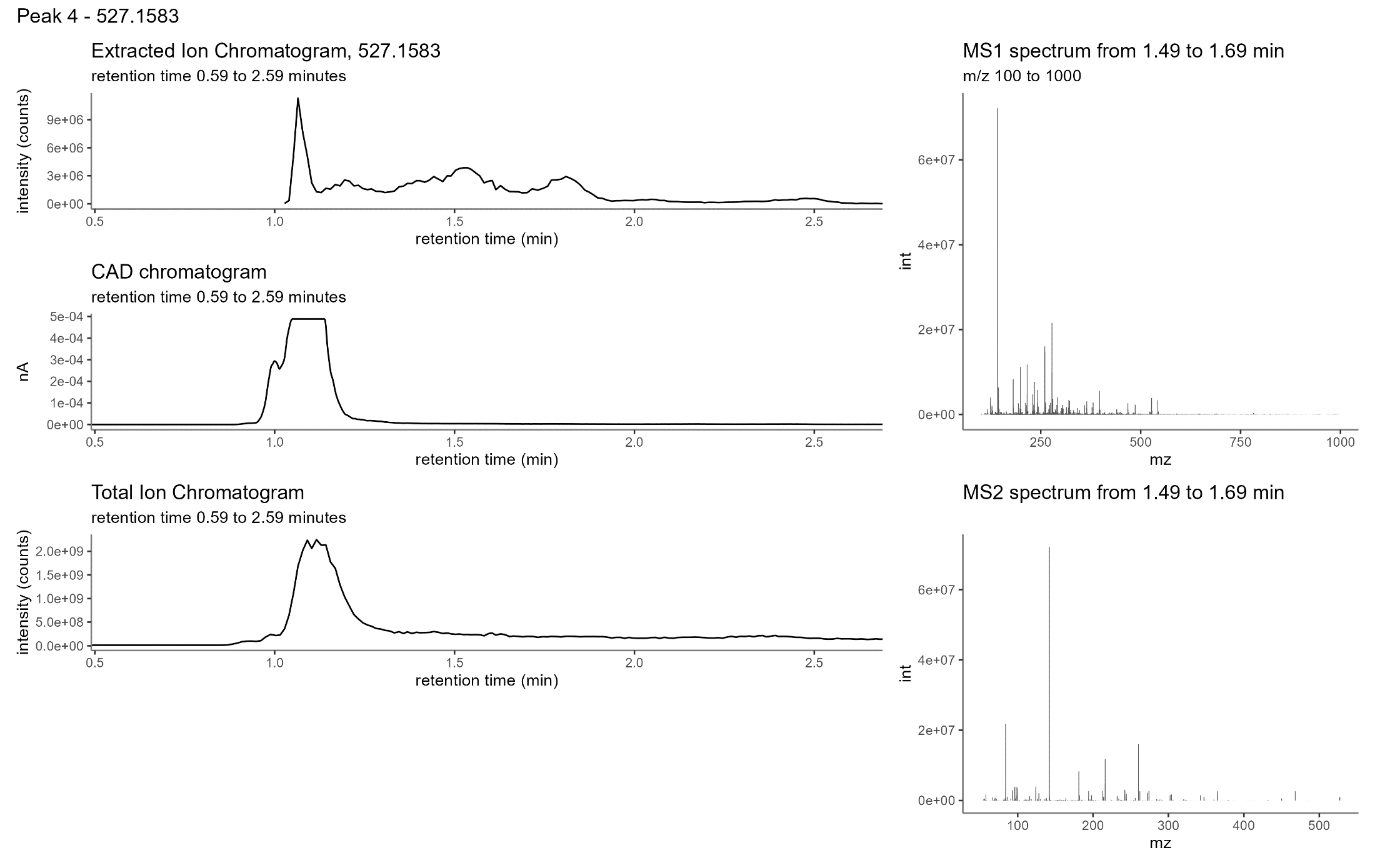 |
| 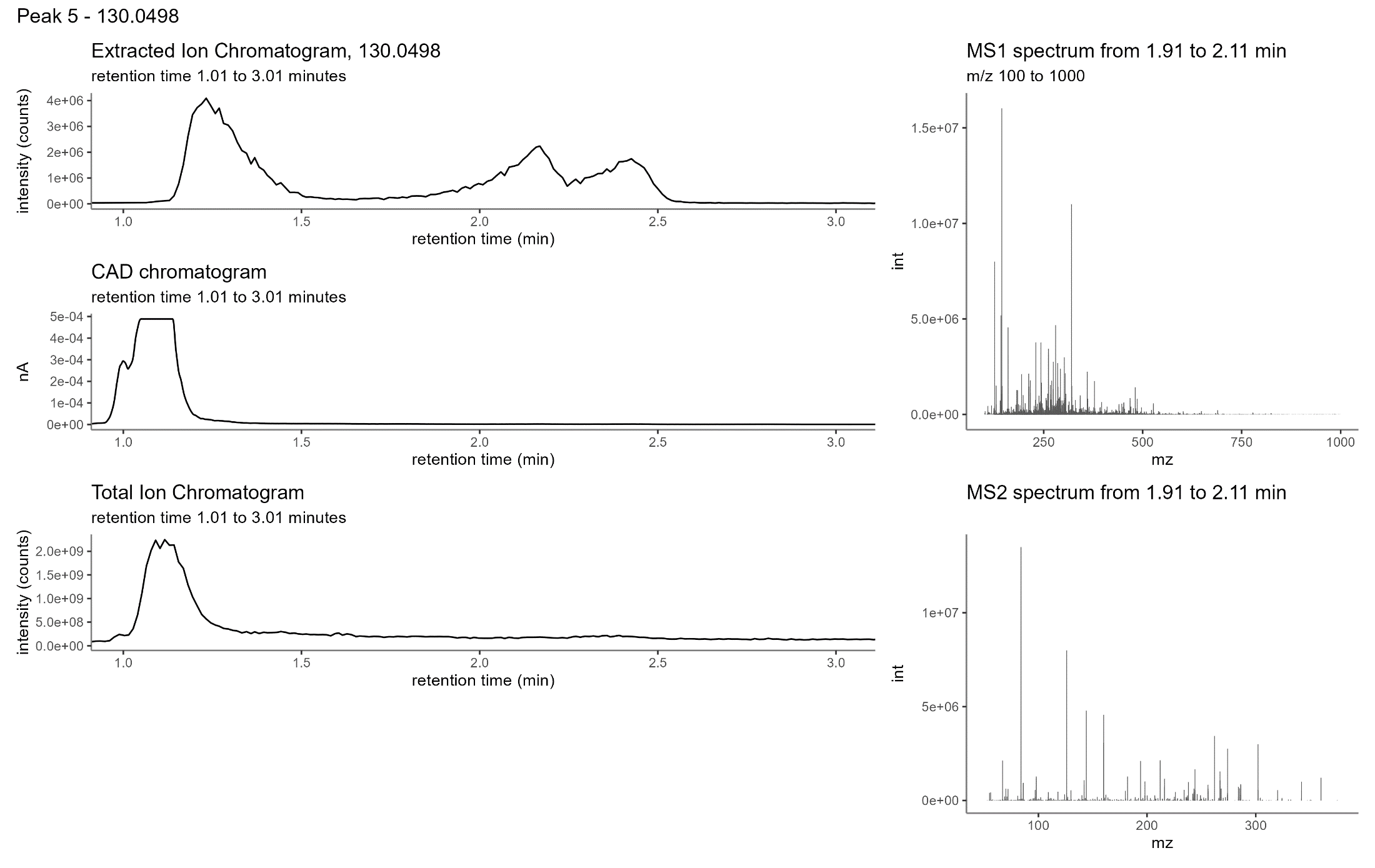 |
| 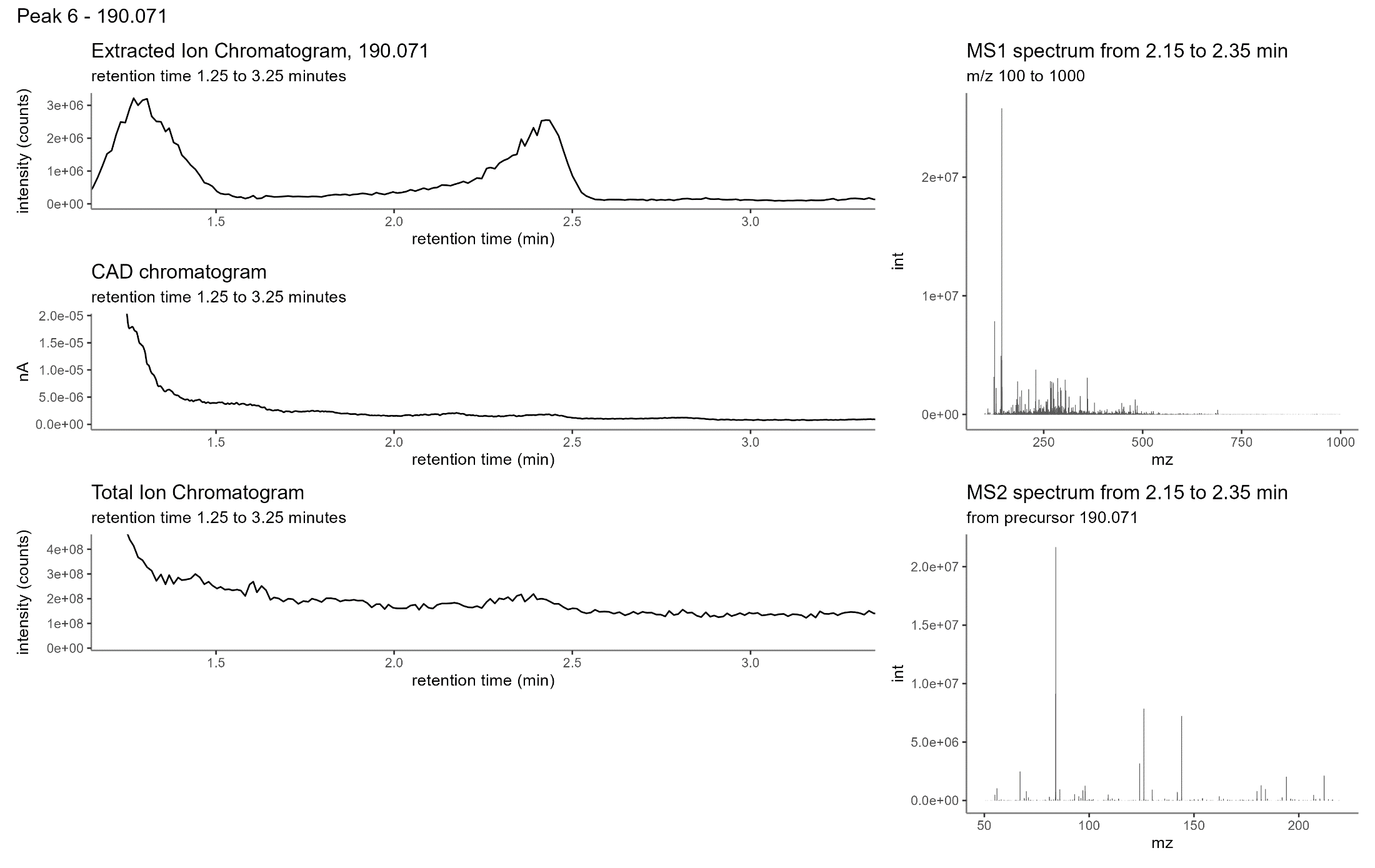 |
| 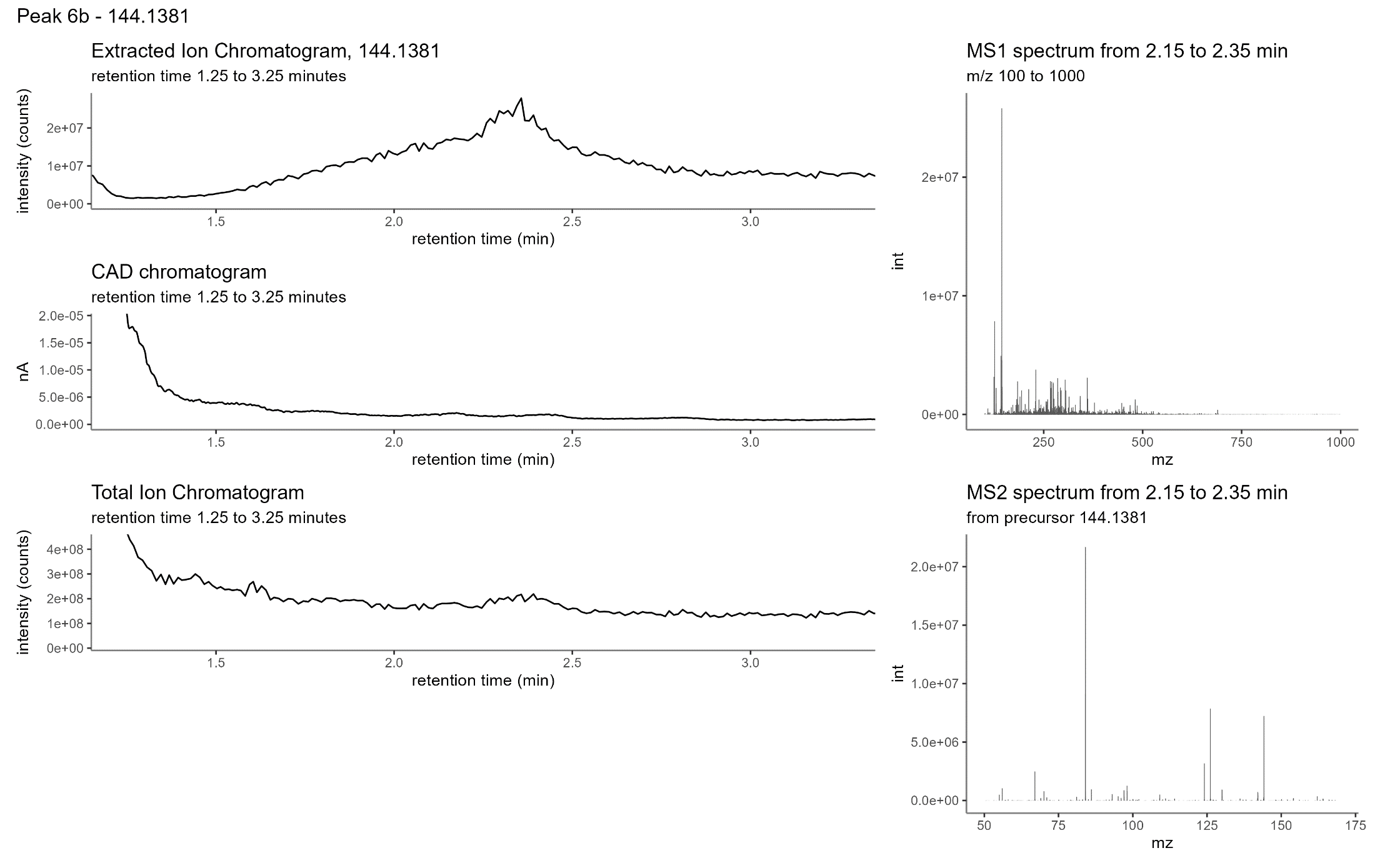 |
| 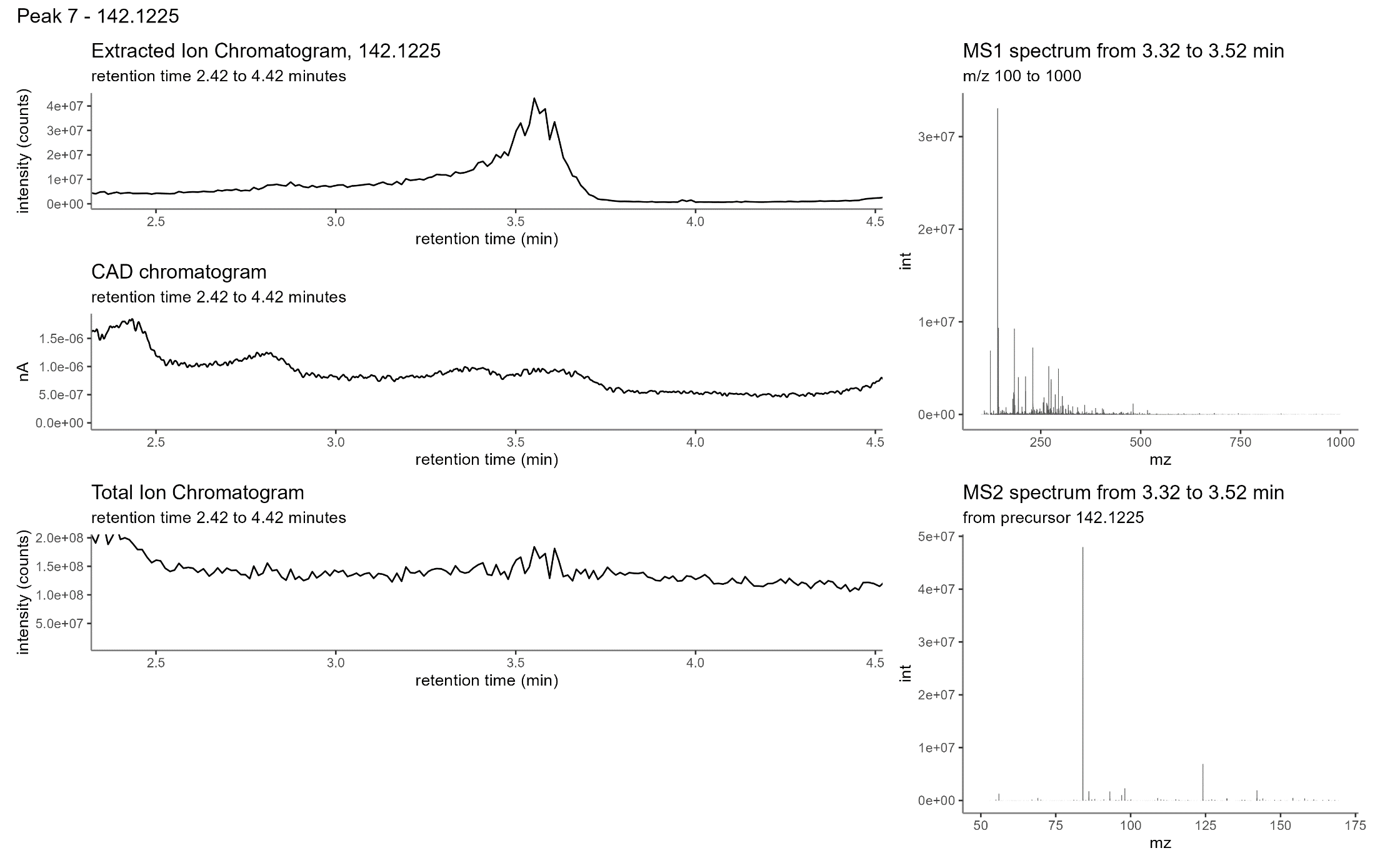 |
| 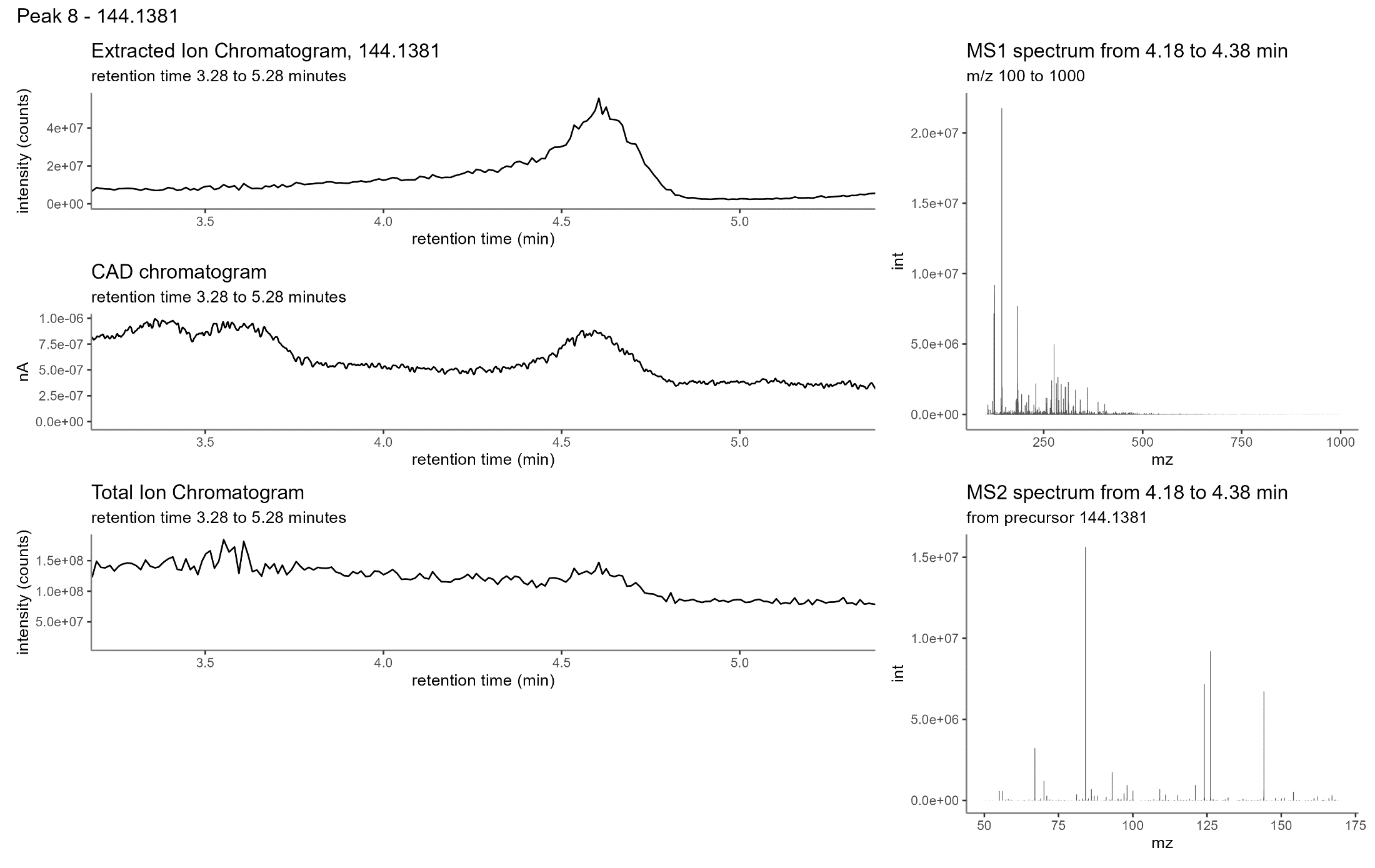 |
| 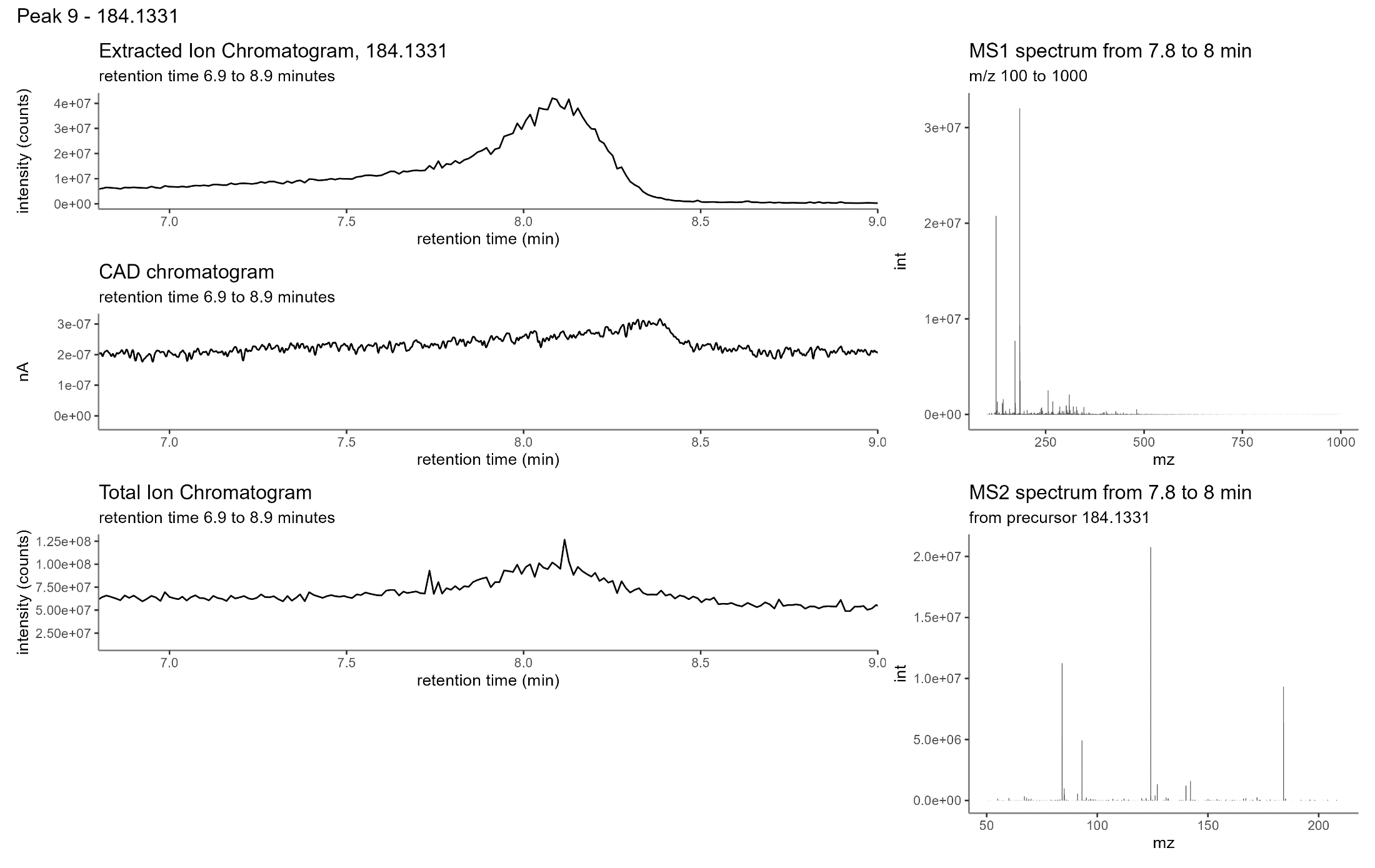 |
| 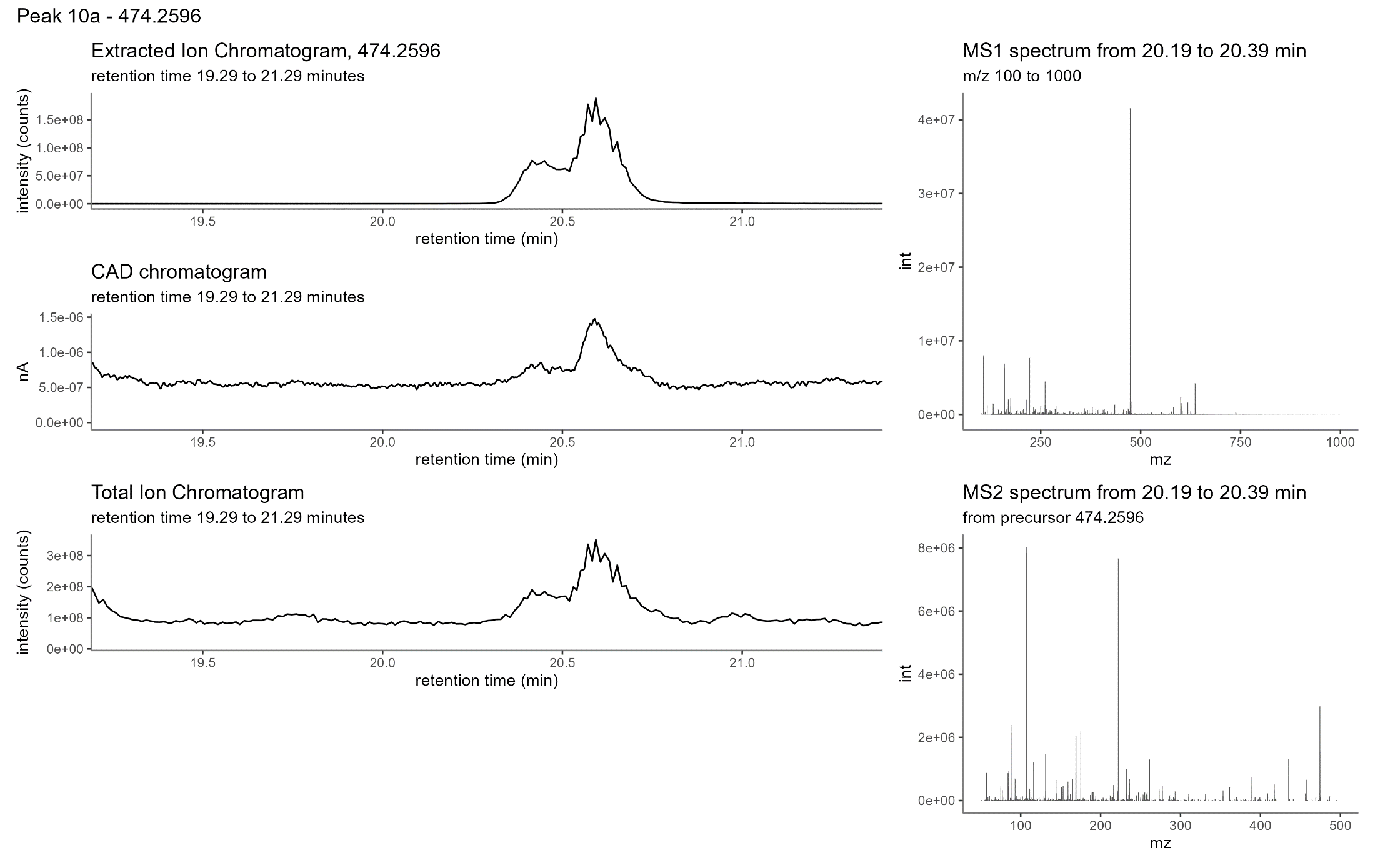 |
| 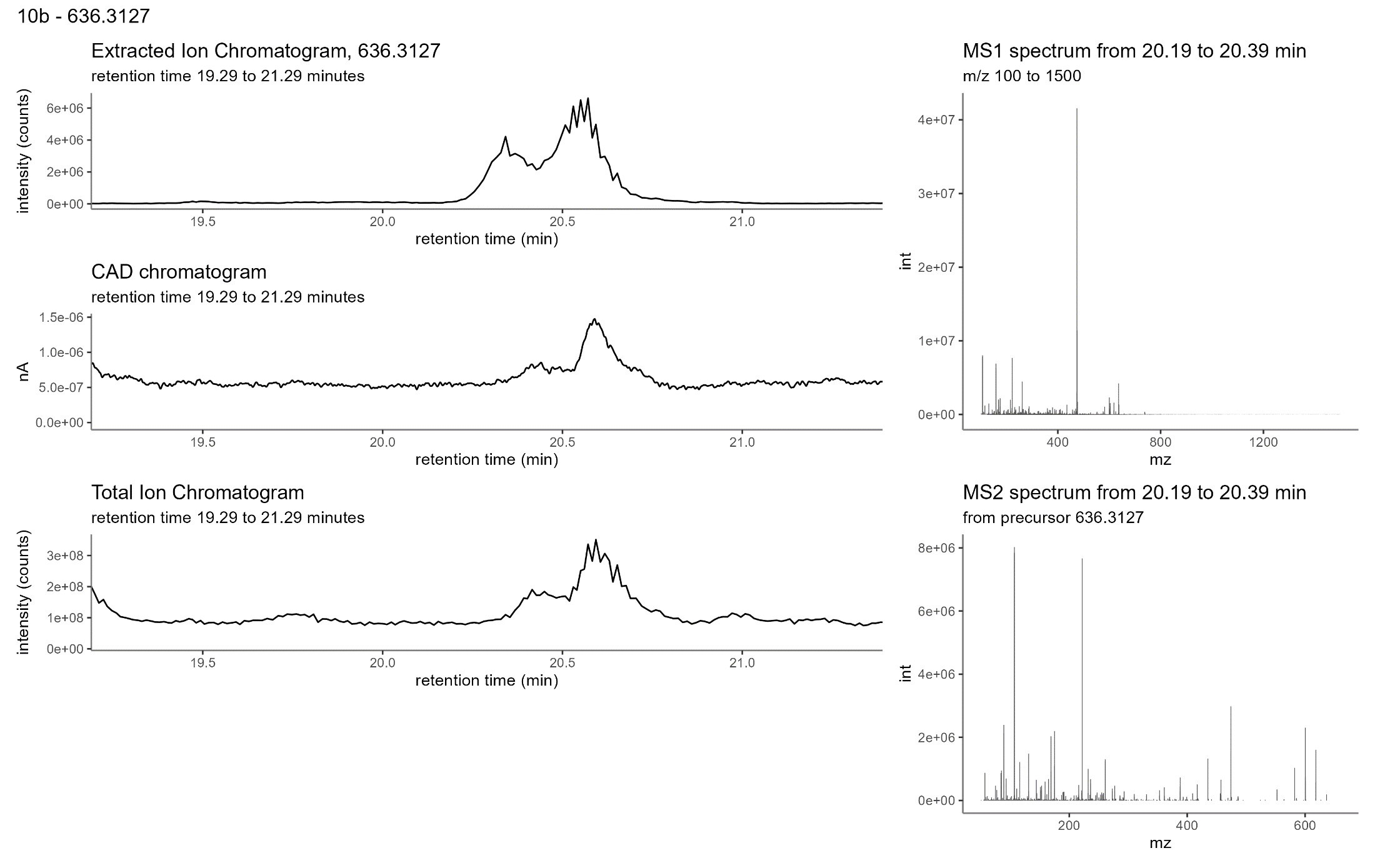 |
| 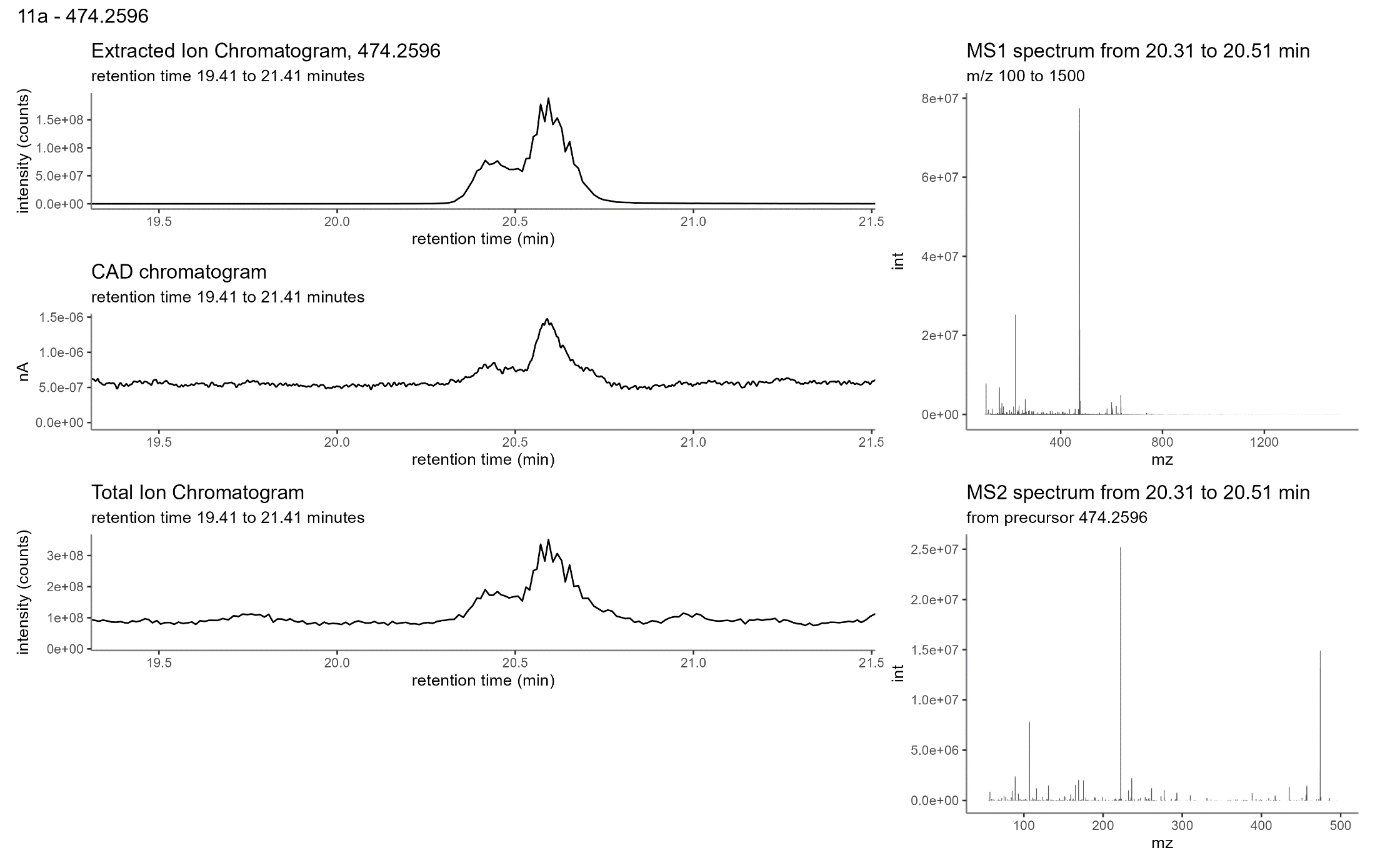 |
| 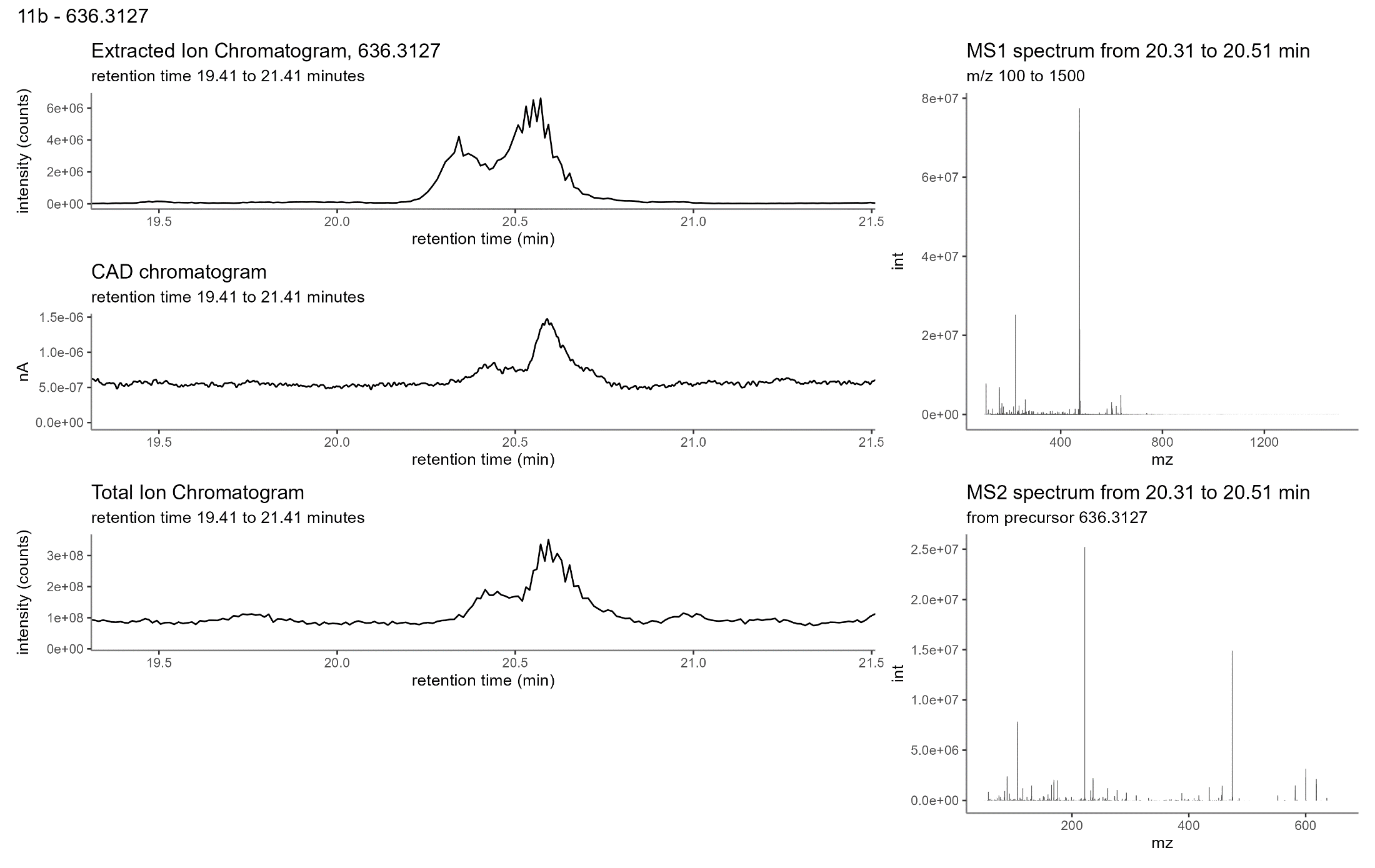 |
| 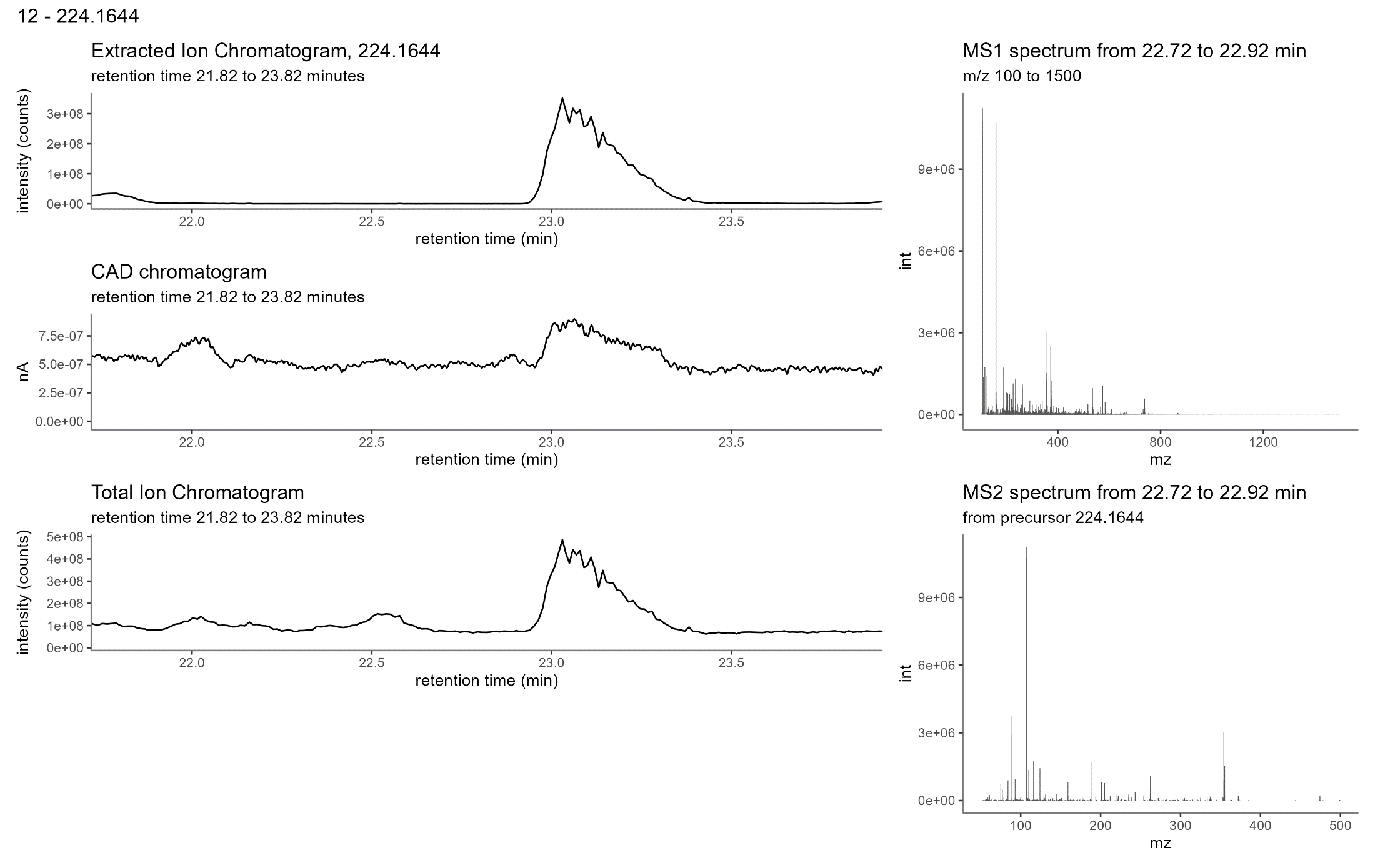 |
| 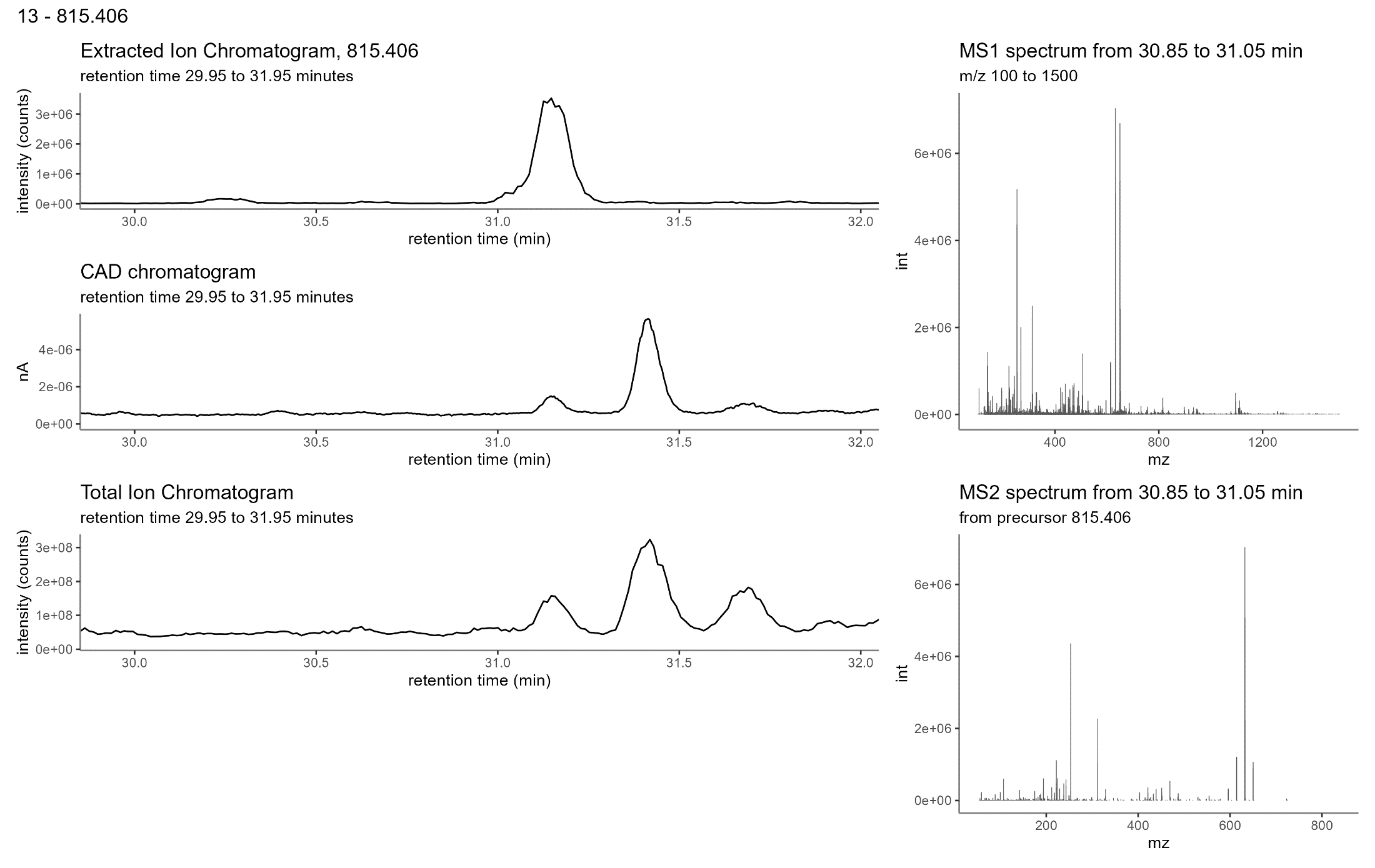 |
| 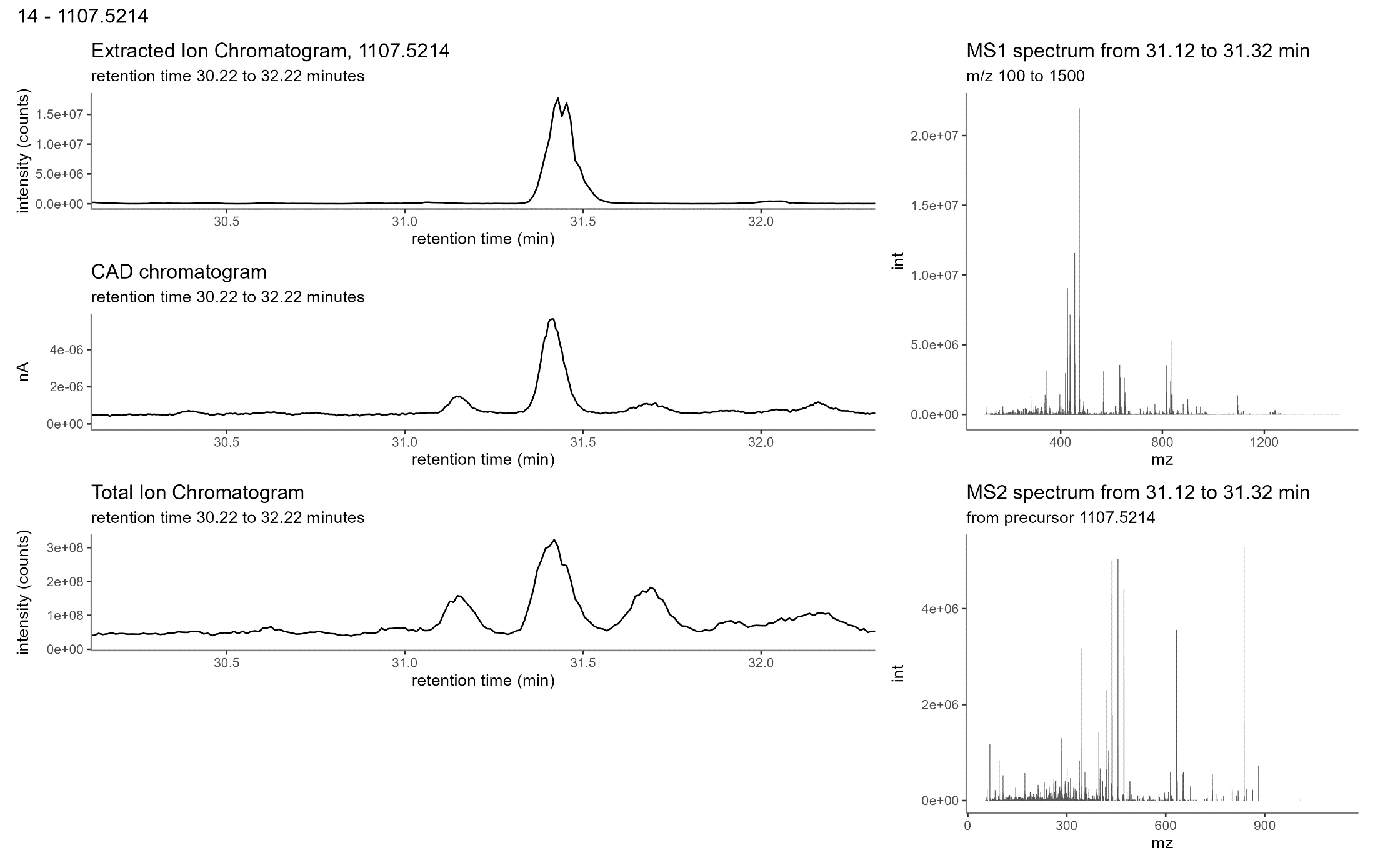 |
| 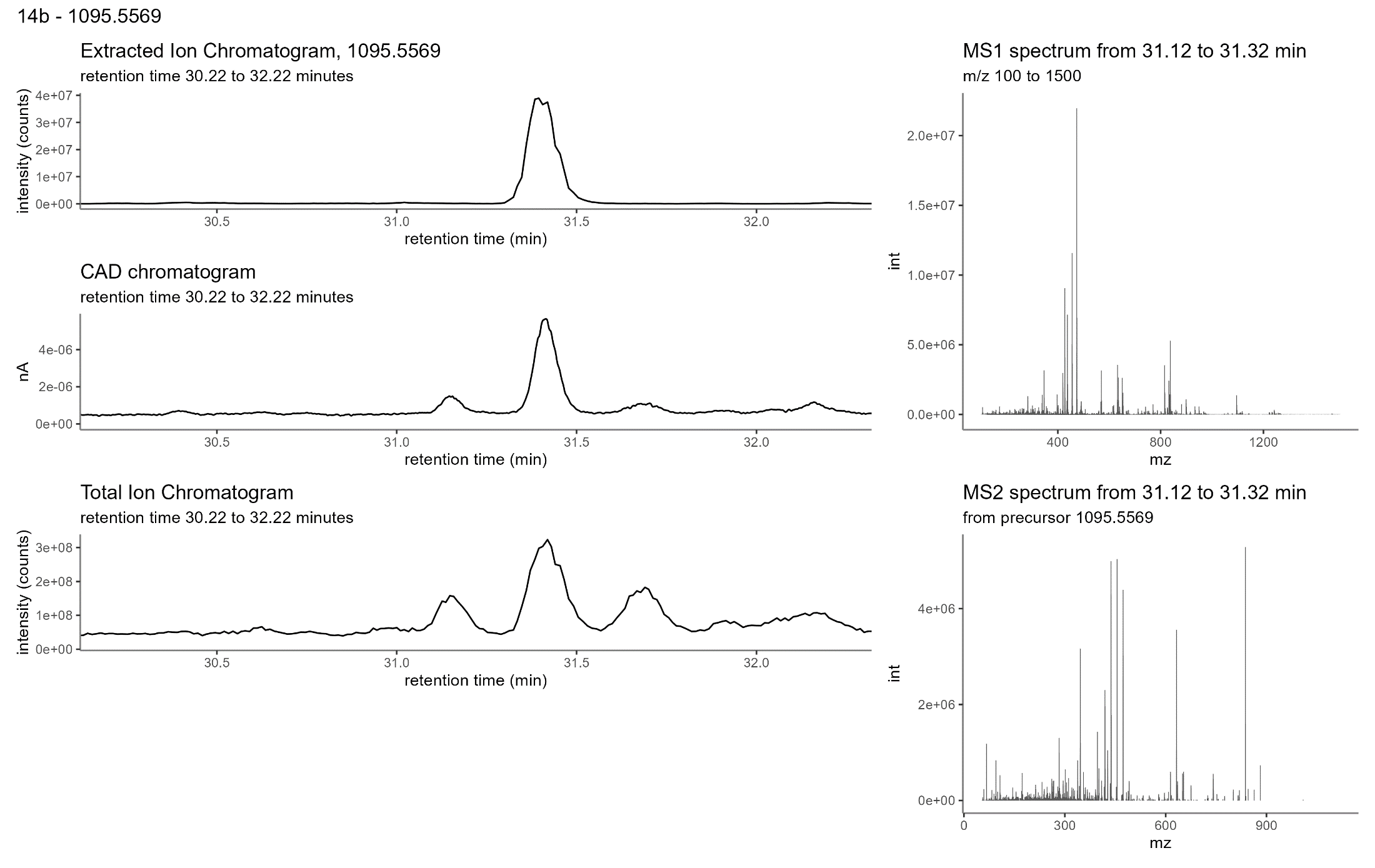 |
| 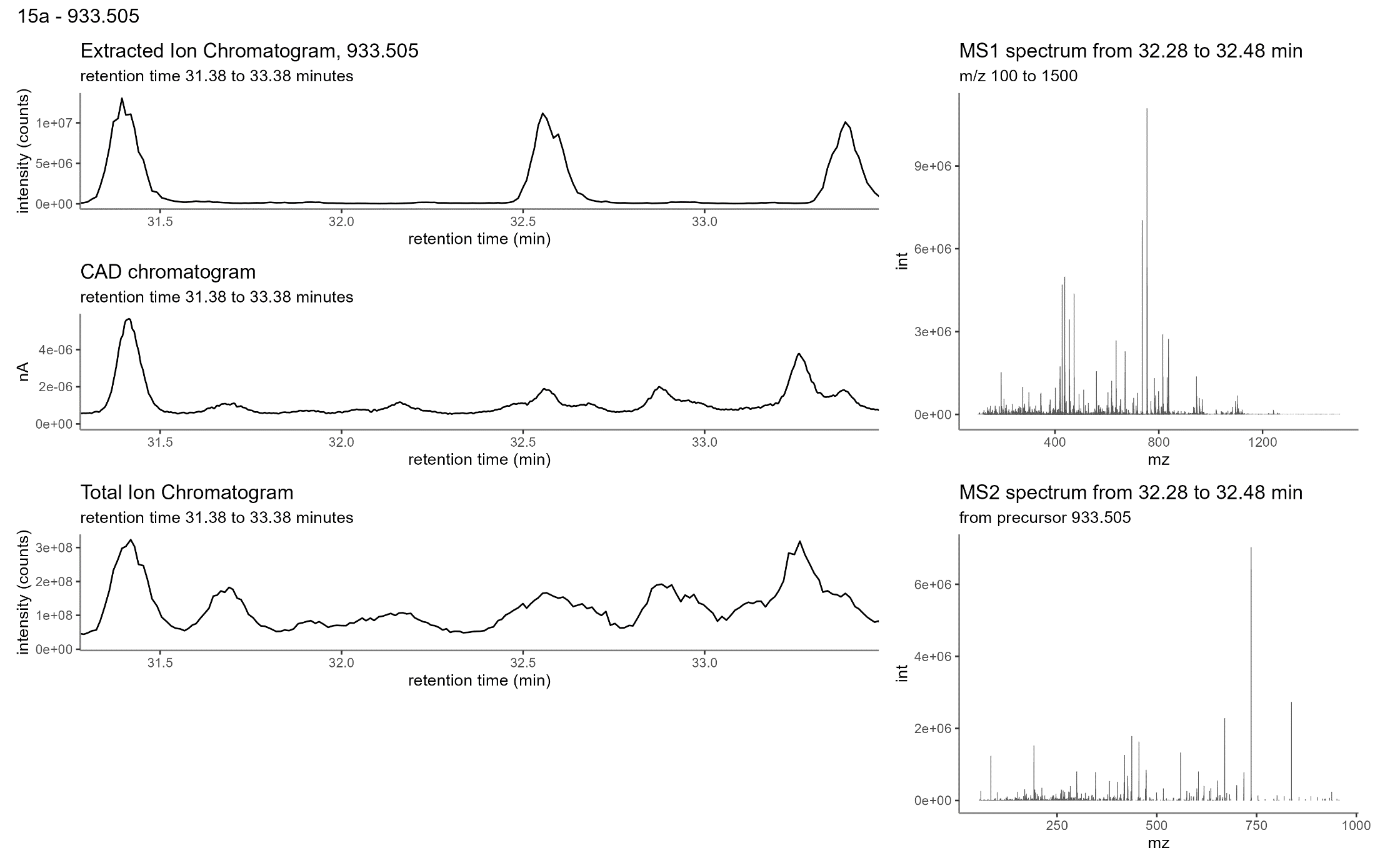 |
| 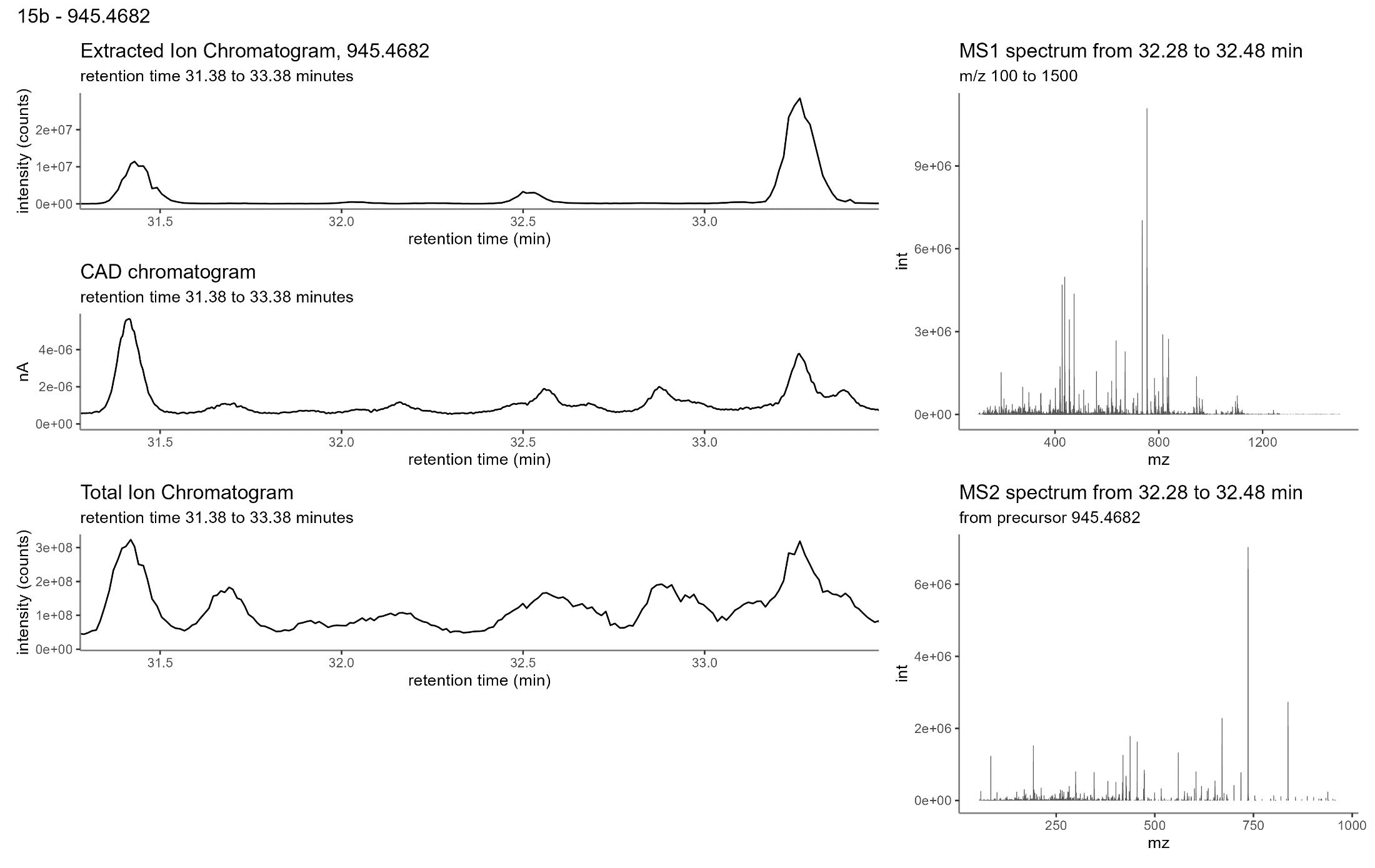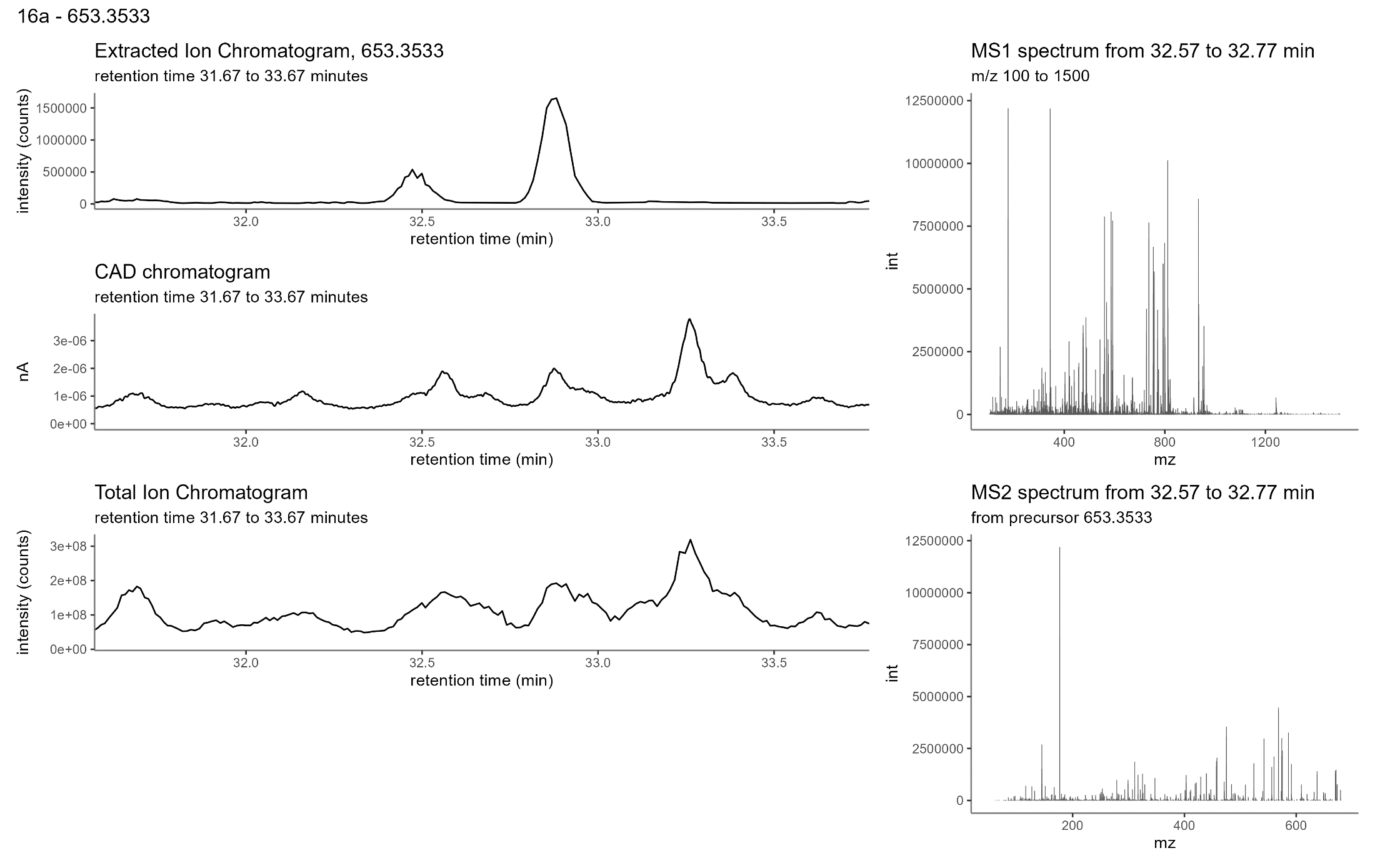 |
| 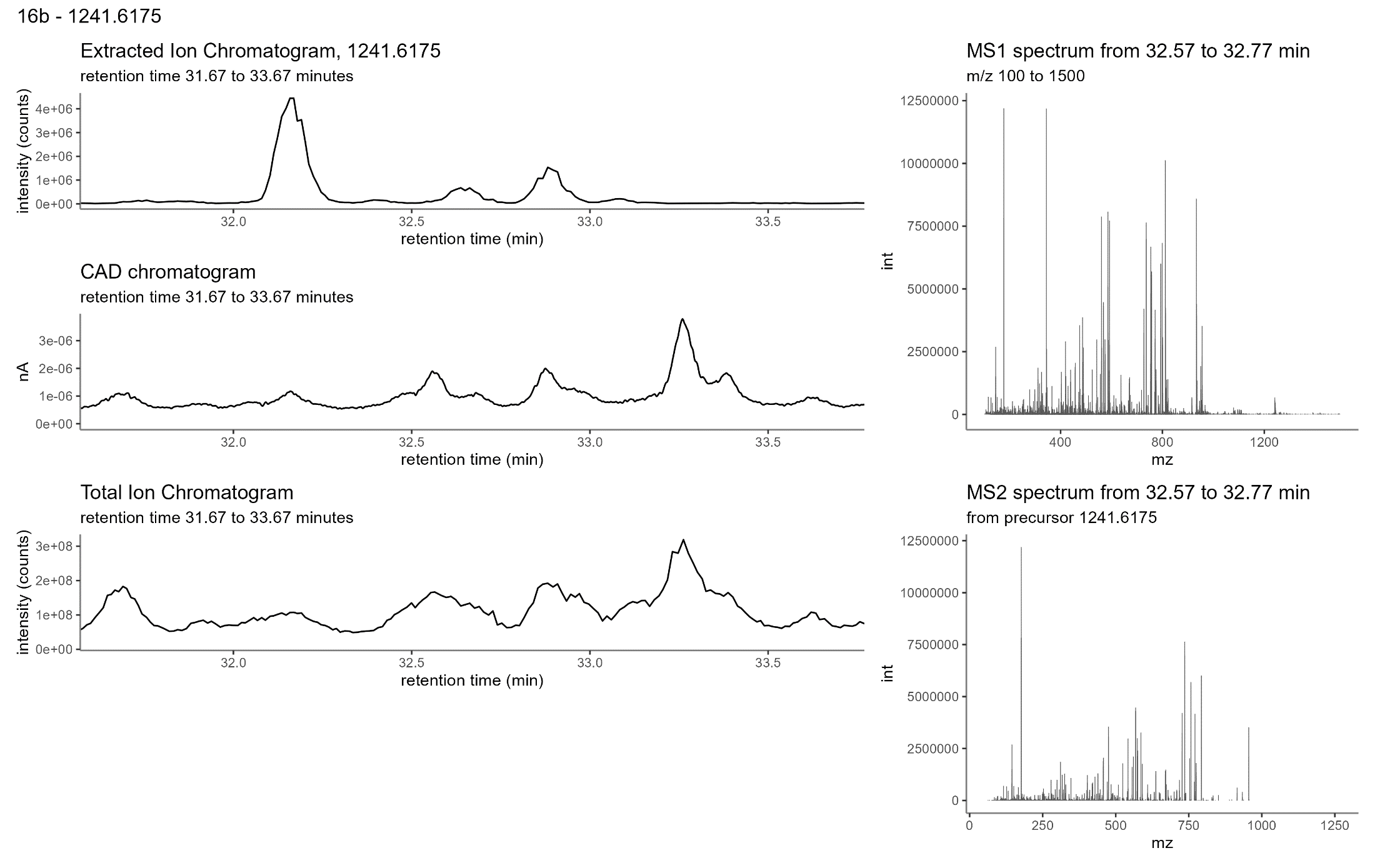 |
| 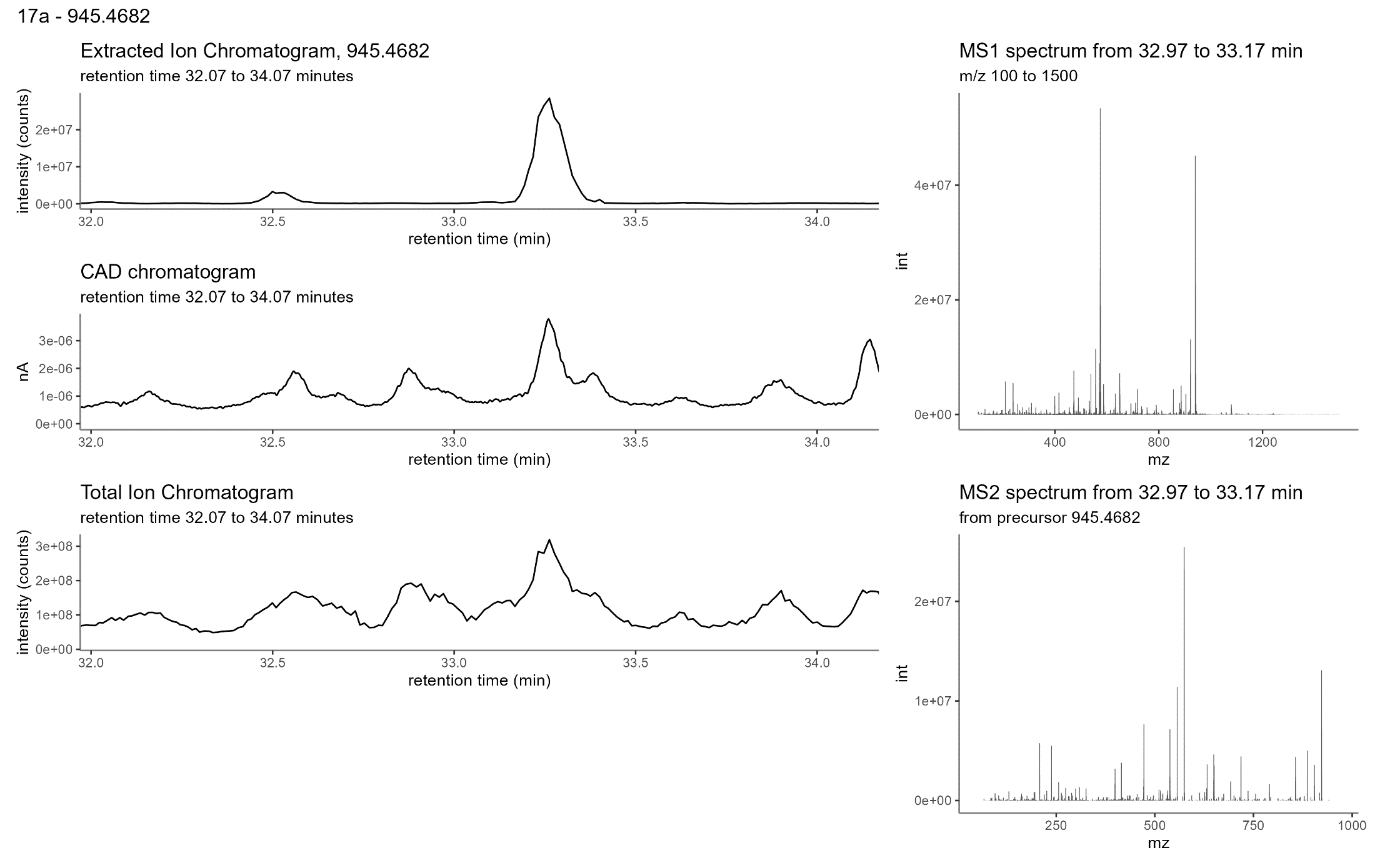 |
| 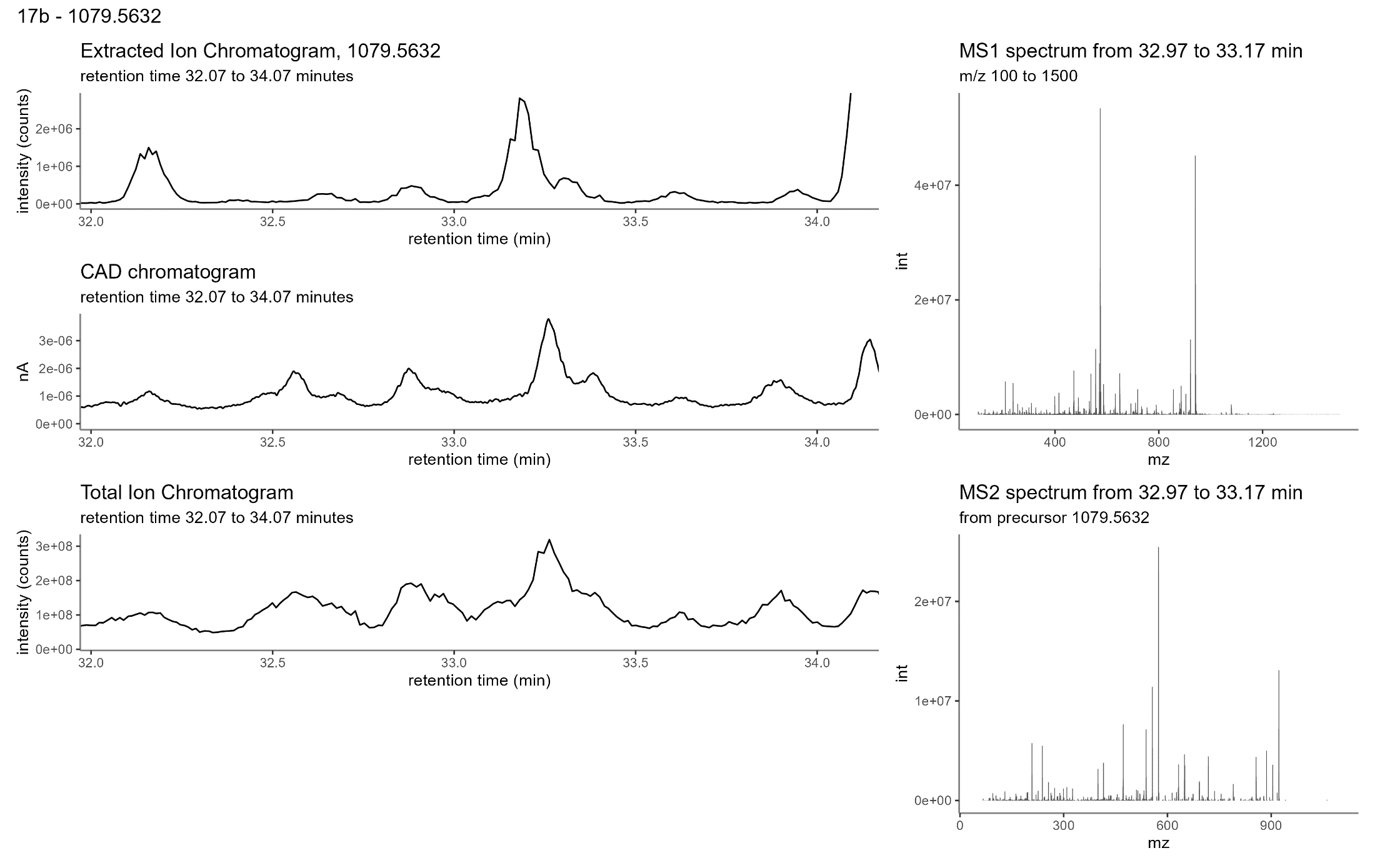 |
| 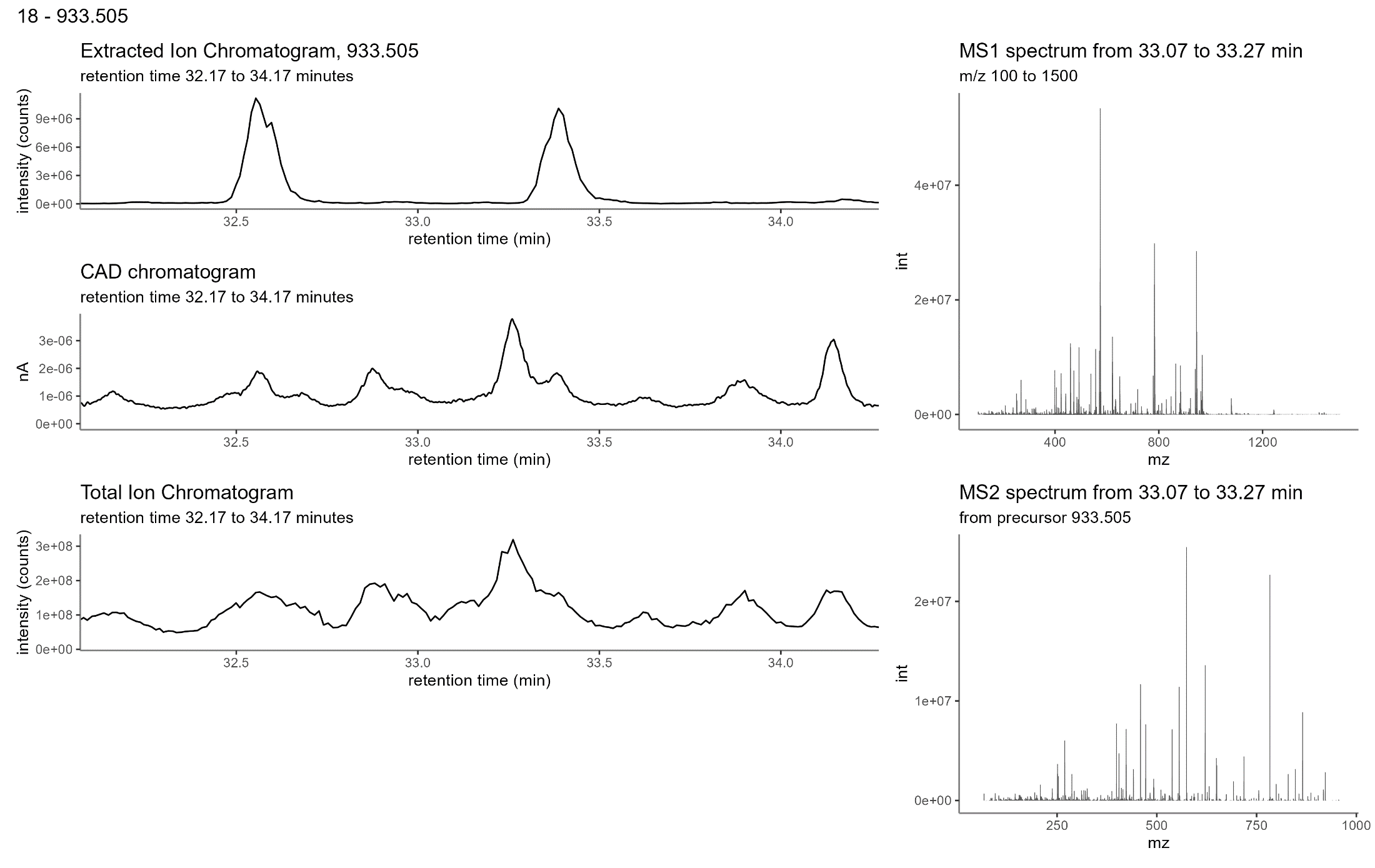 |
| 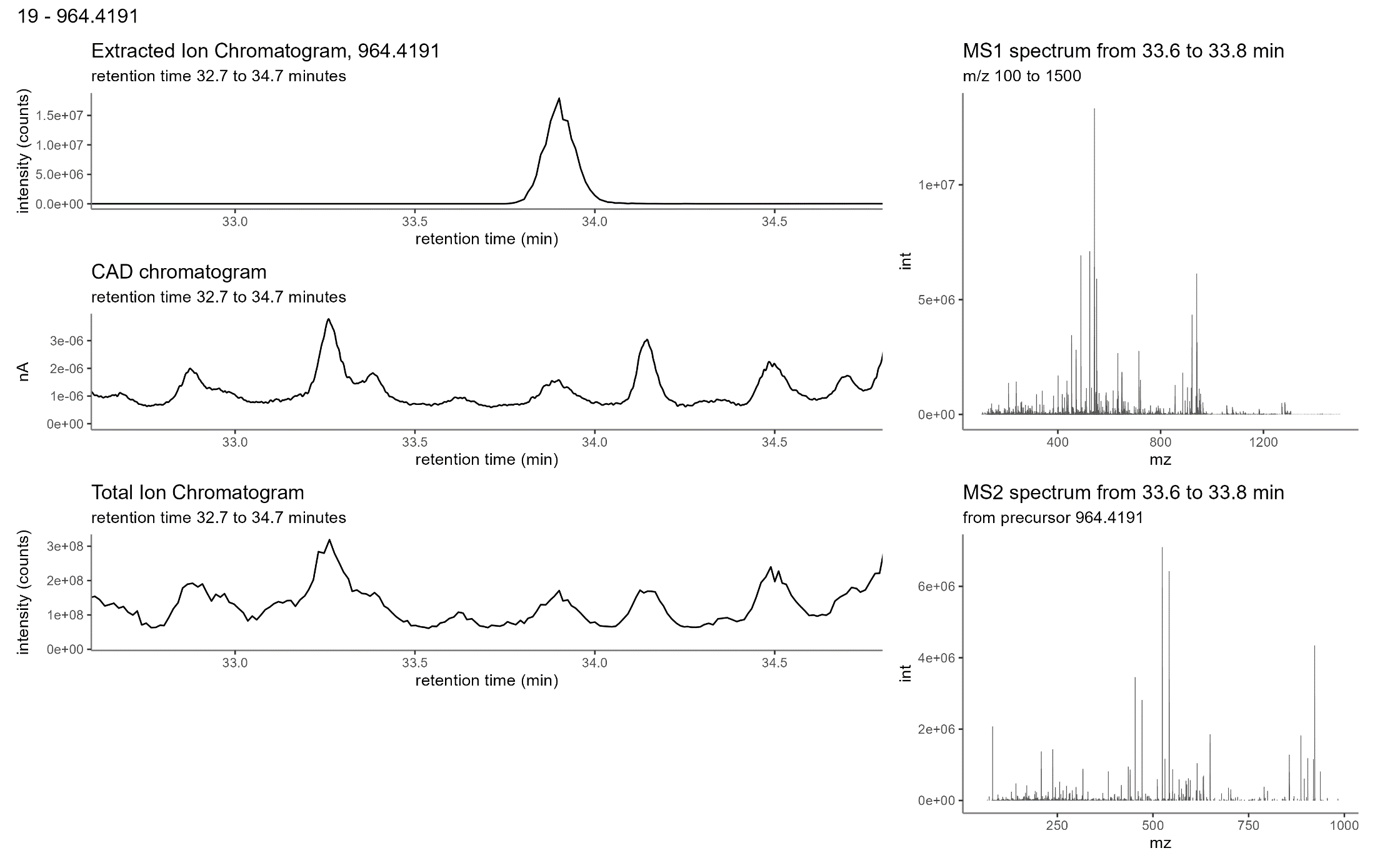 |
| 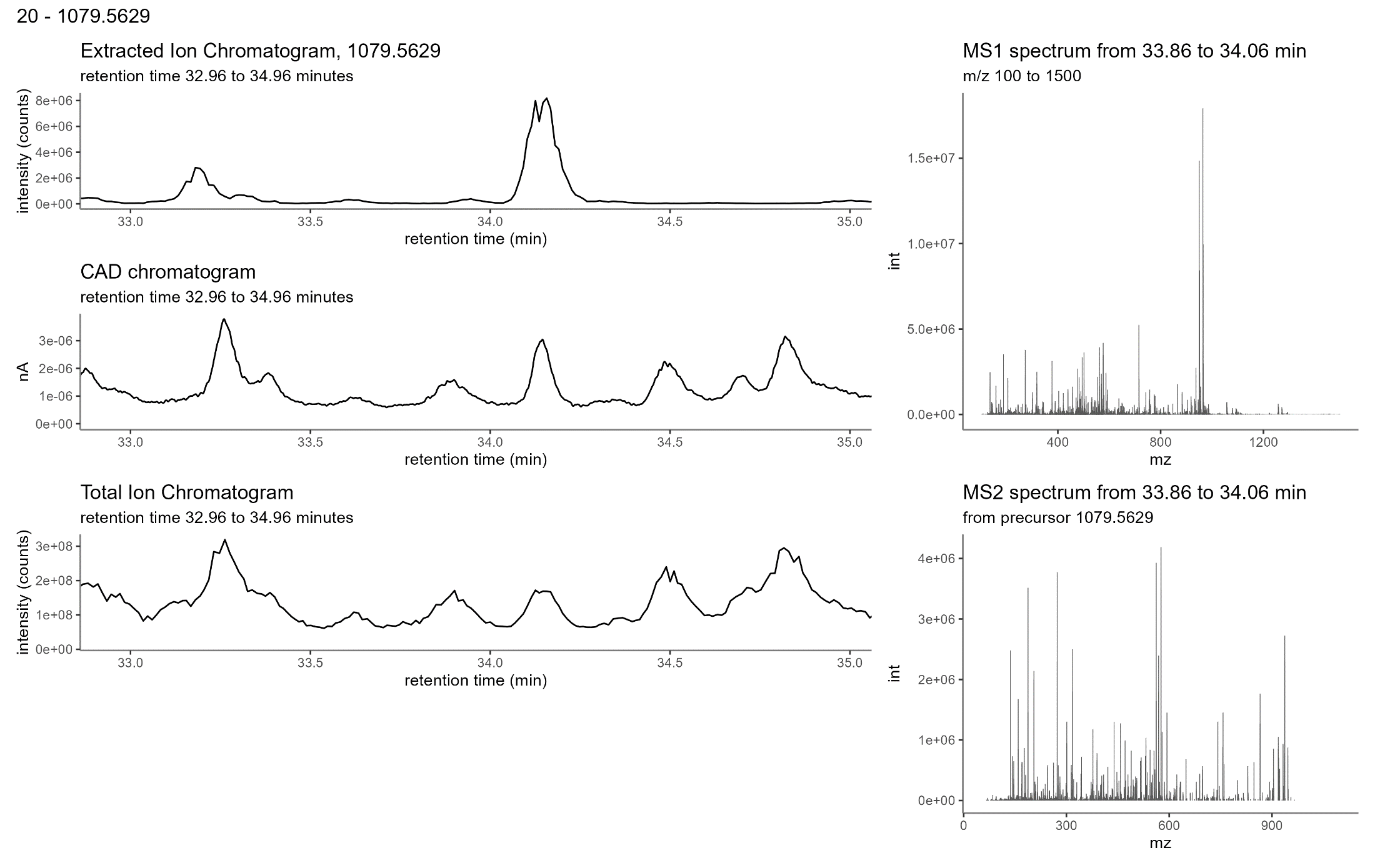 |
| 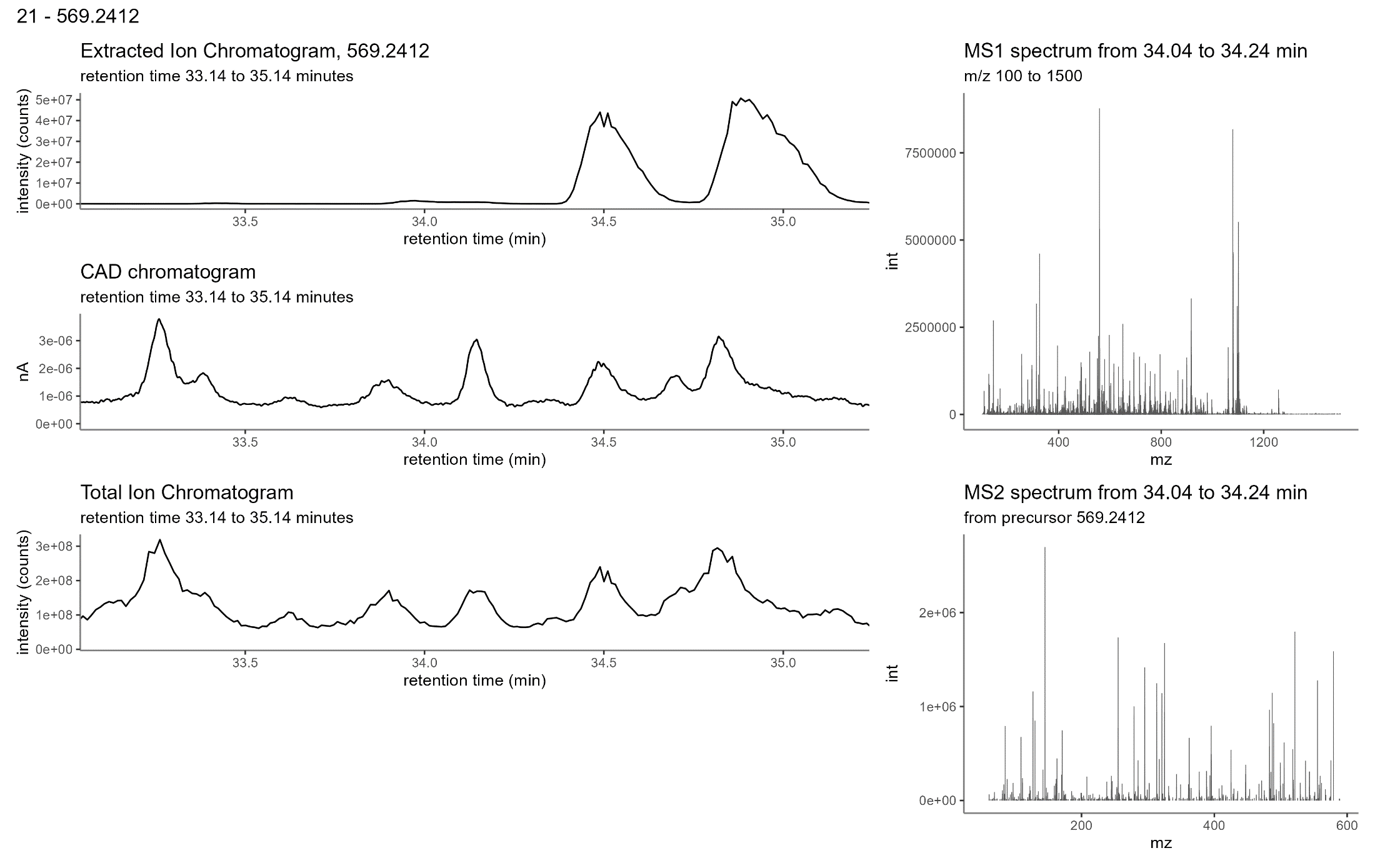 |
| 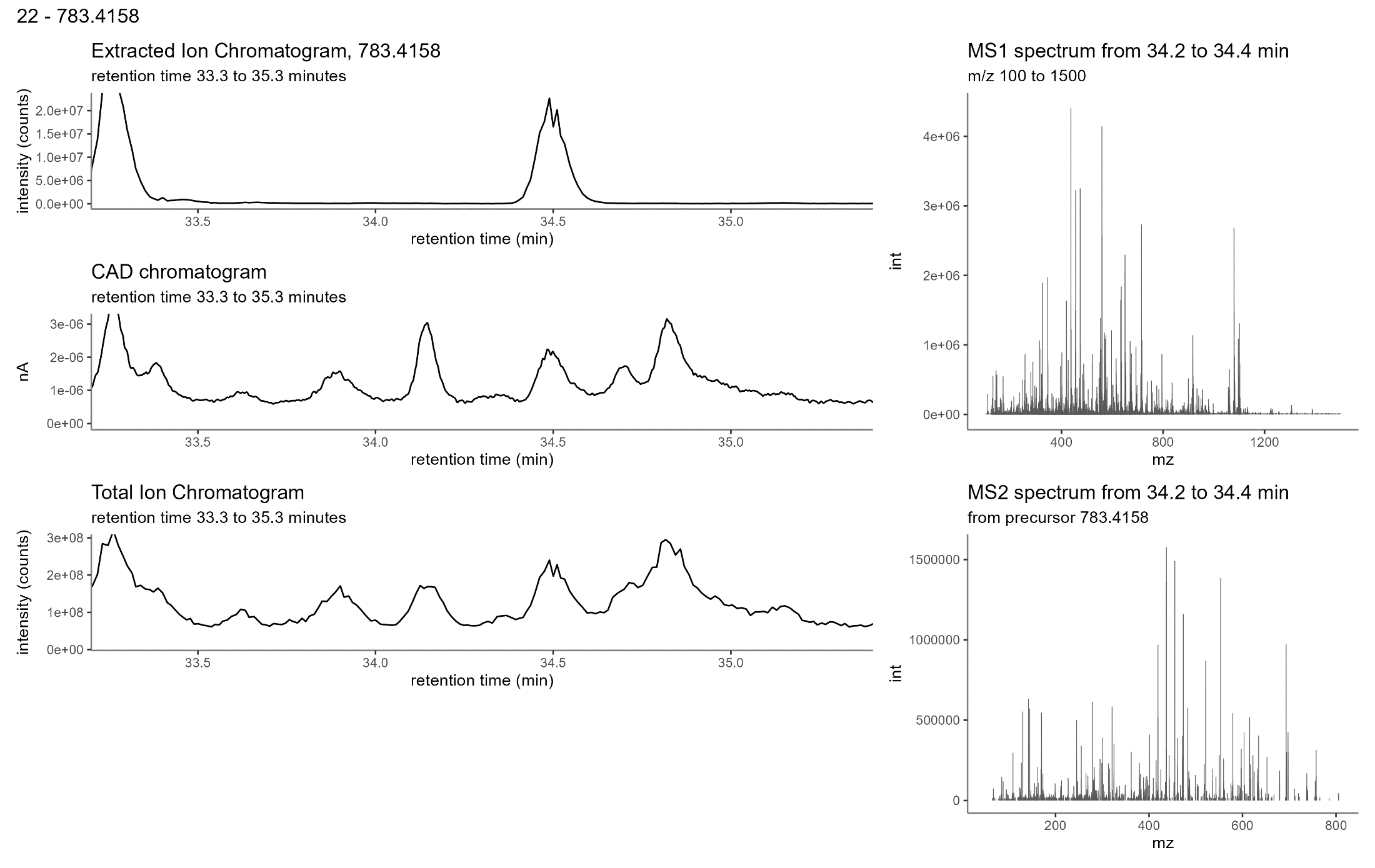 |
| 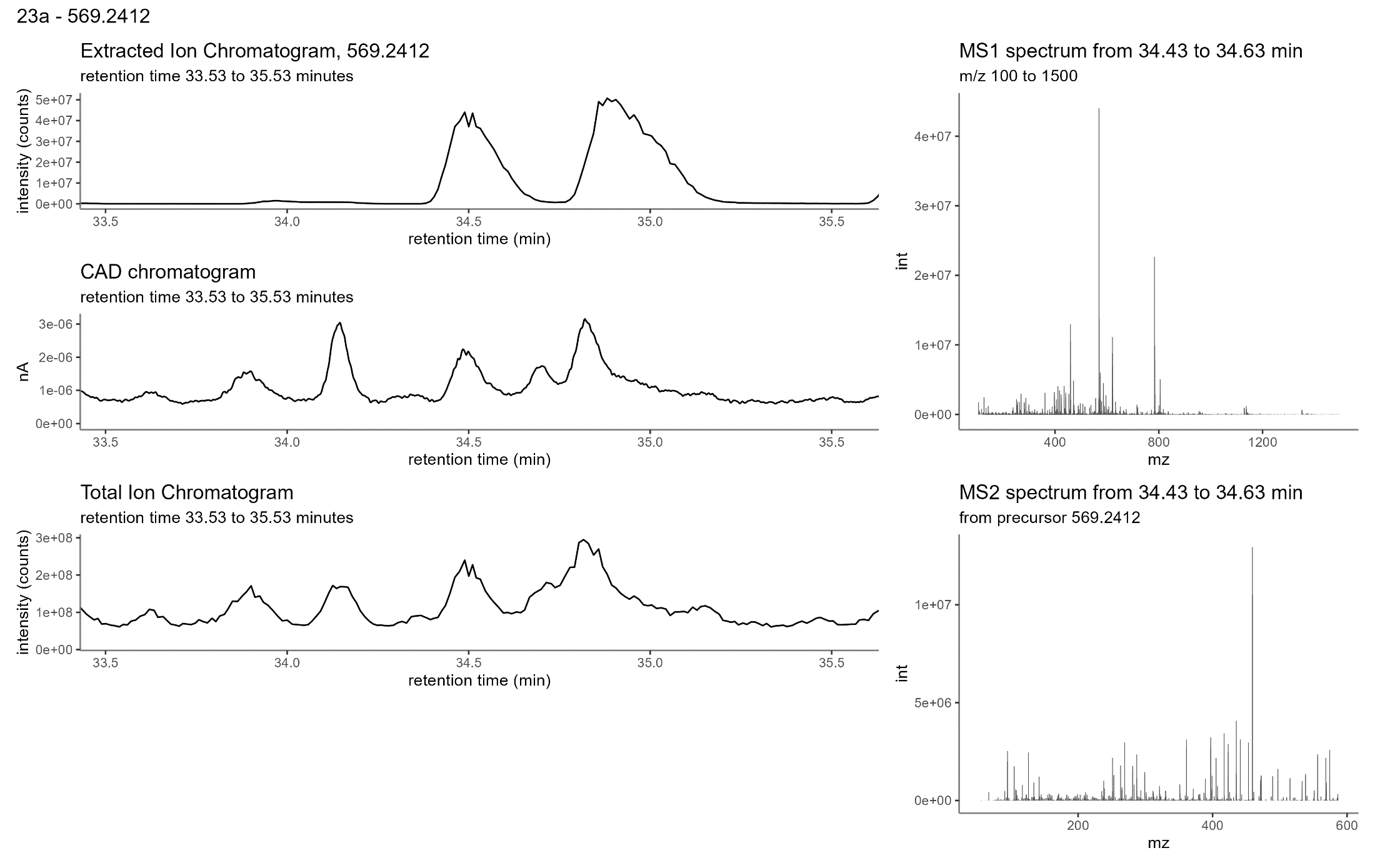 |
| 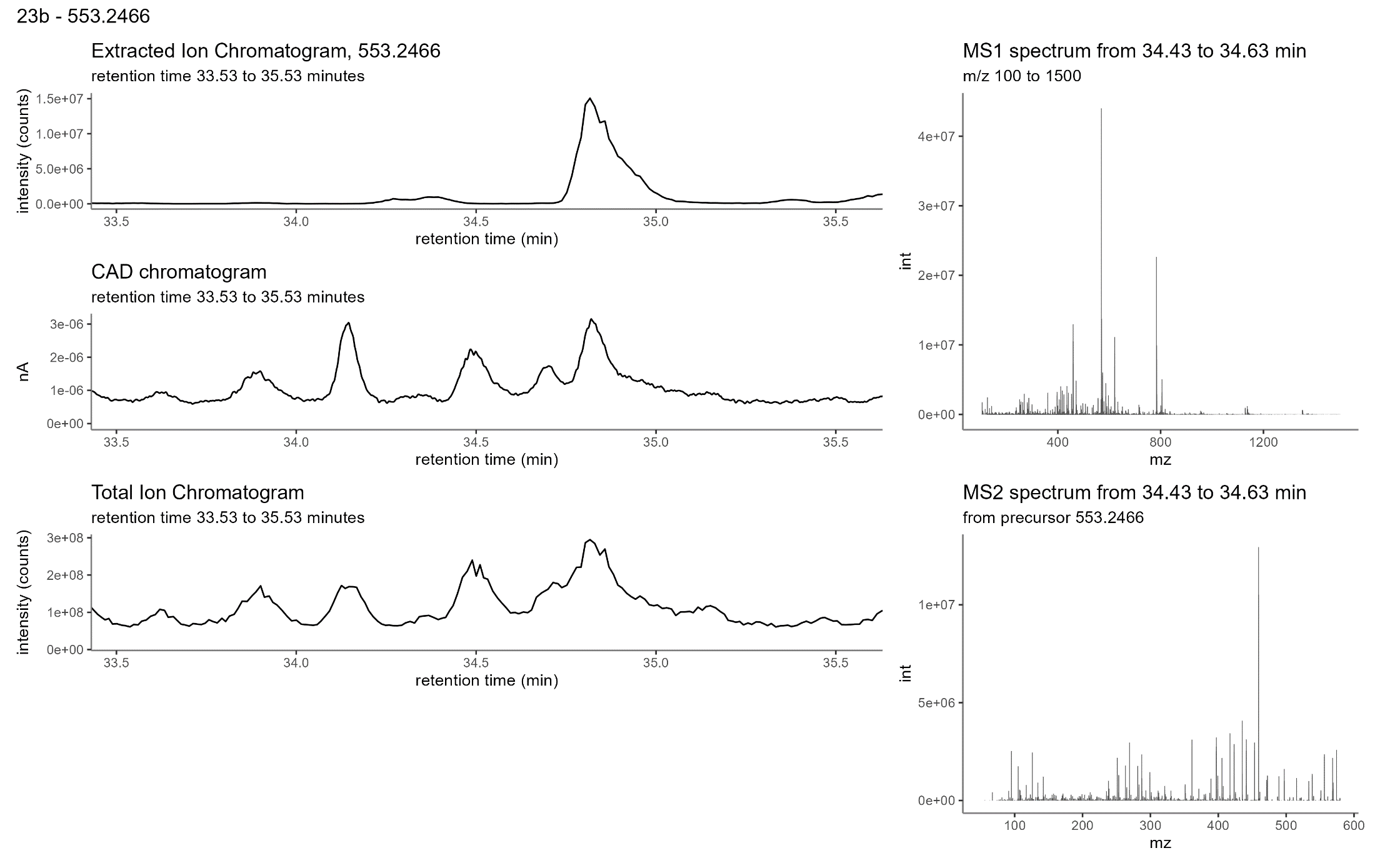 |
| 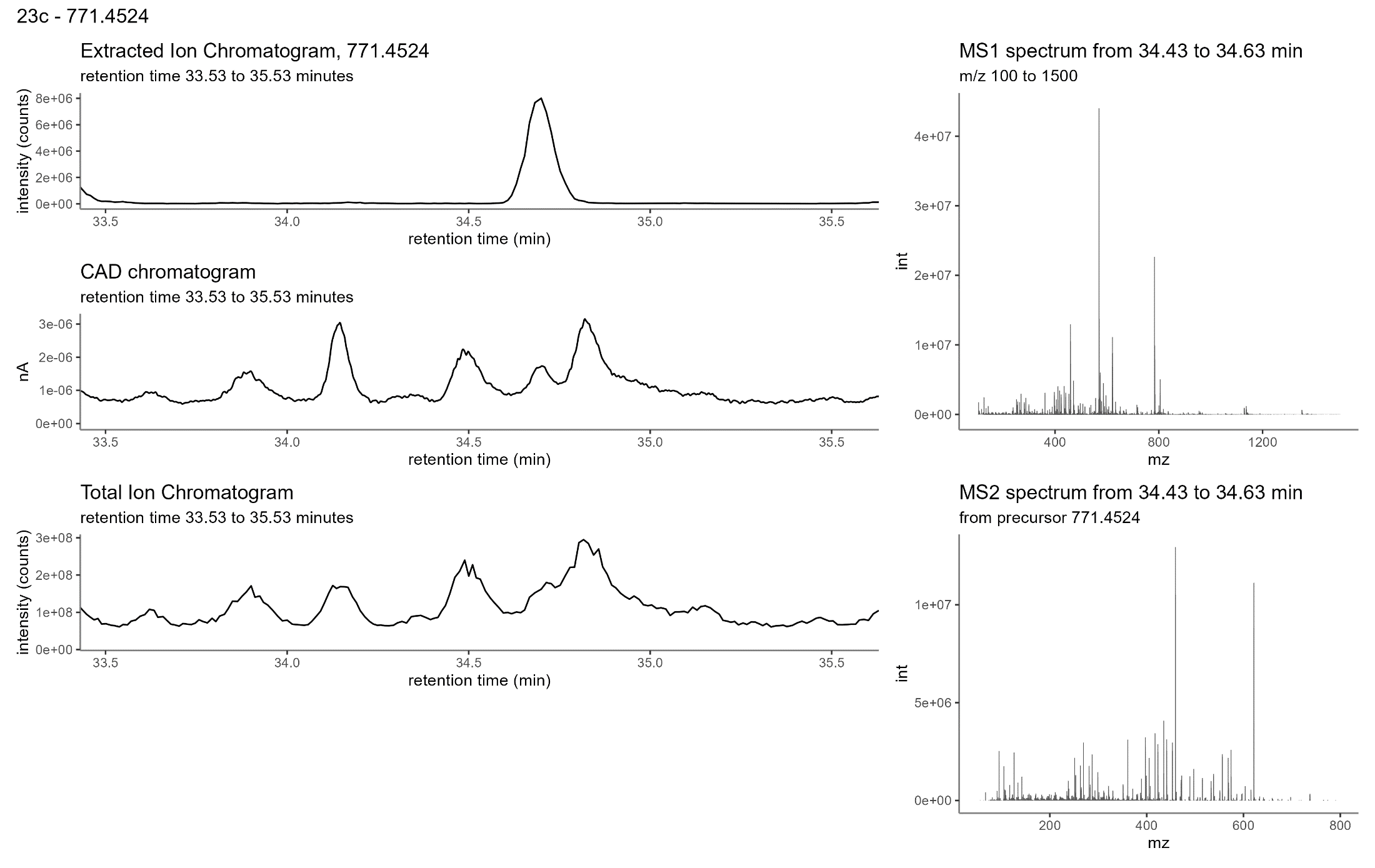 |
| 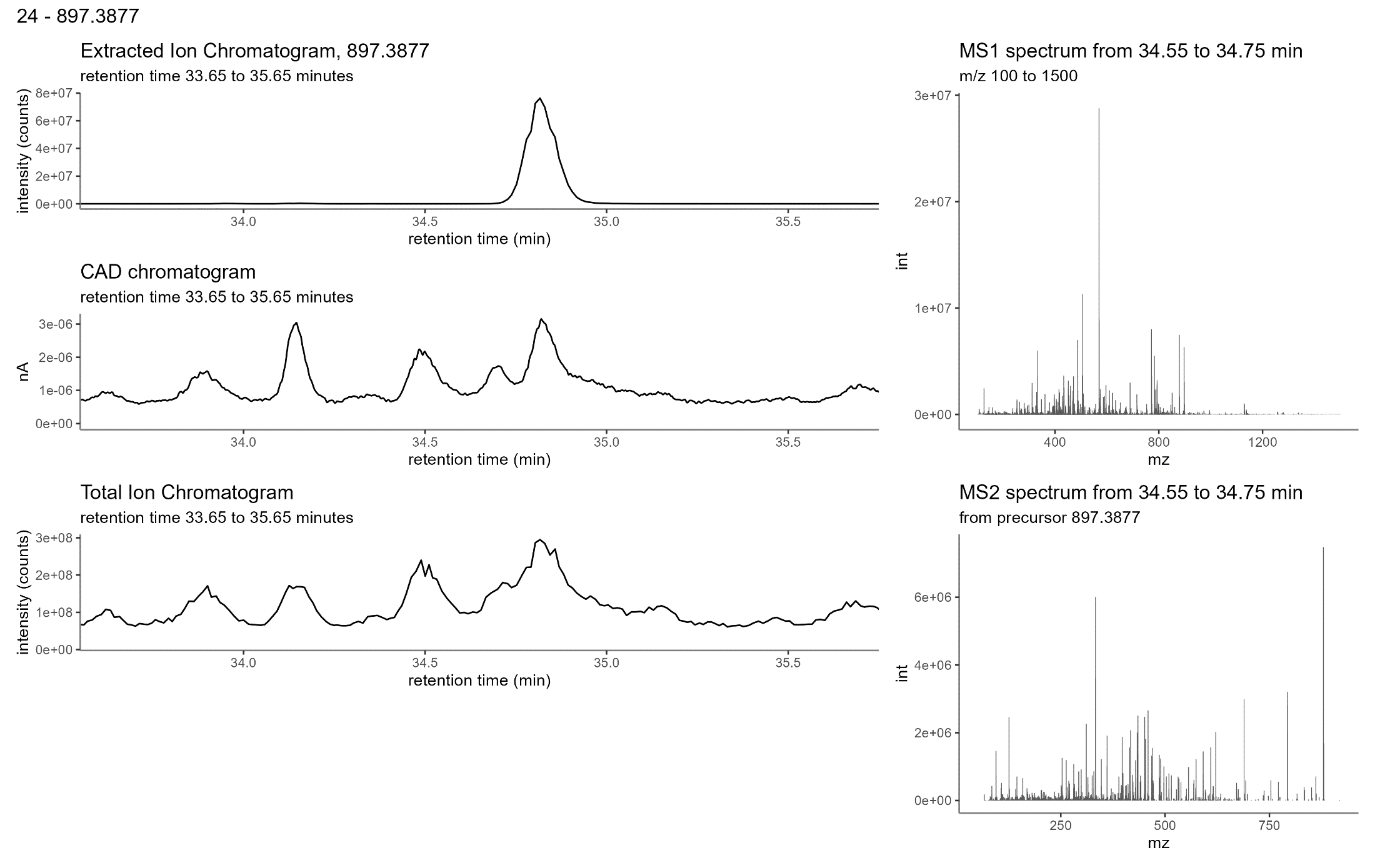 |
| 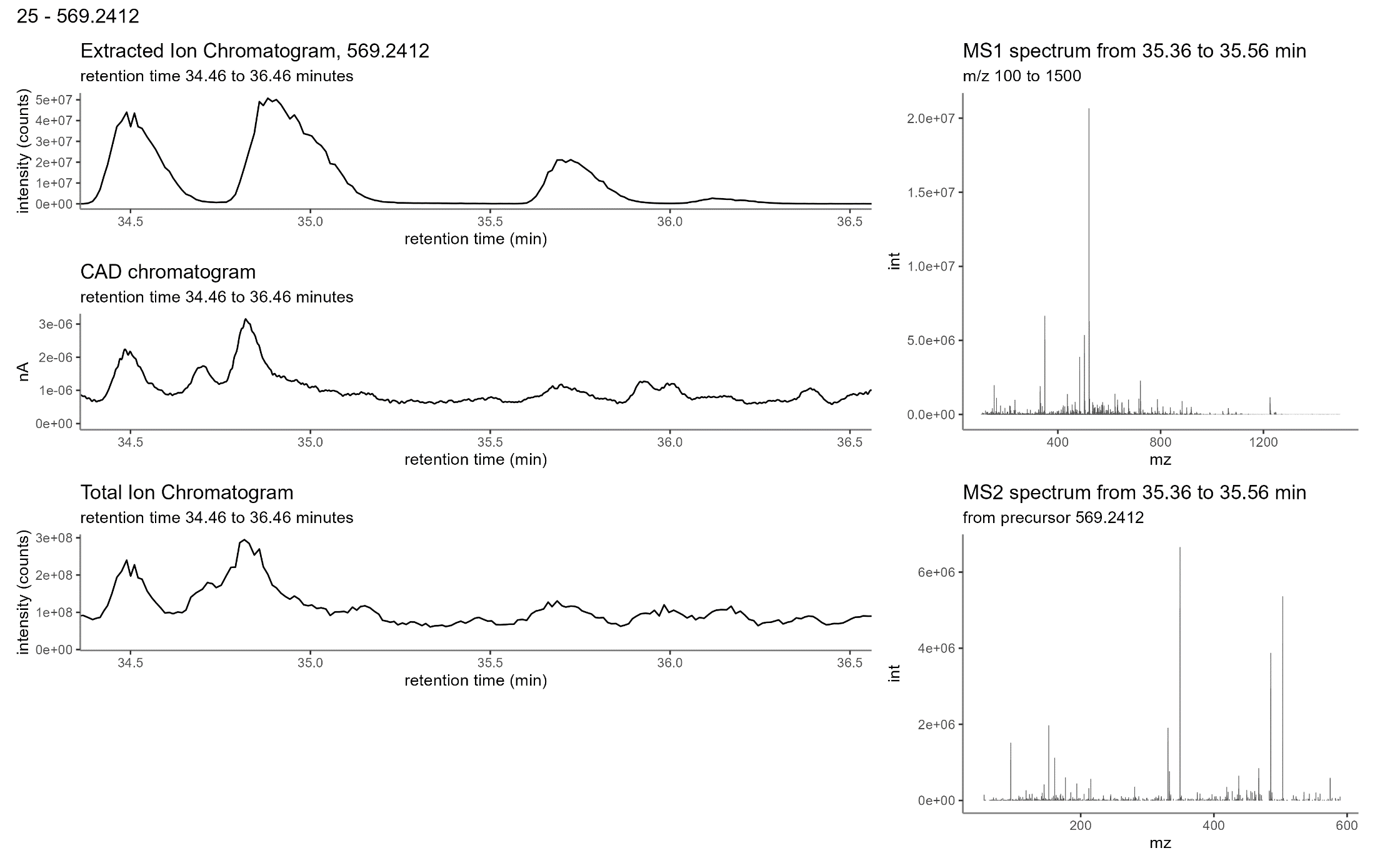 |
| 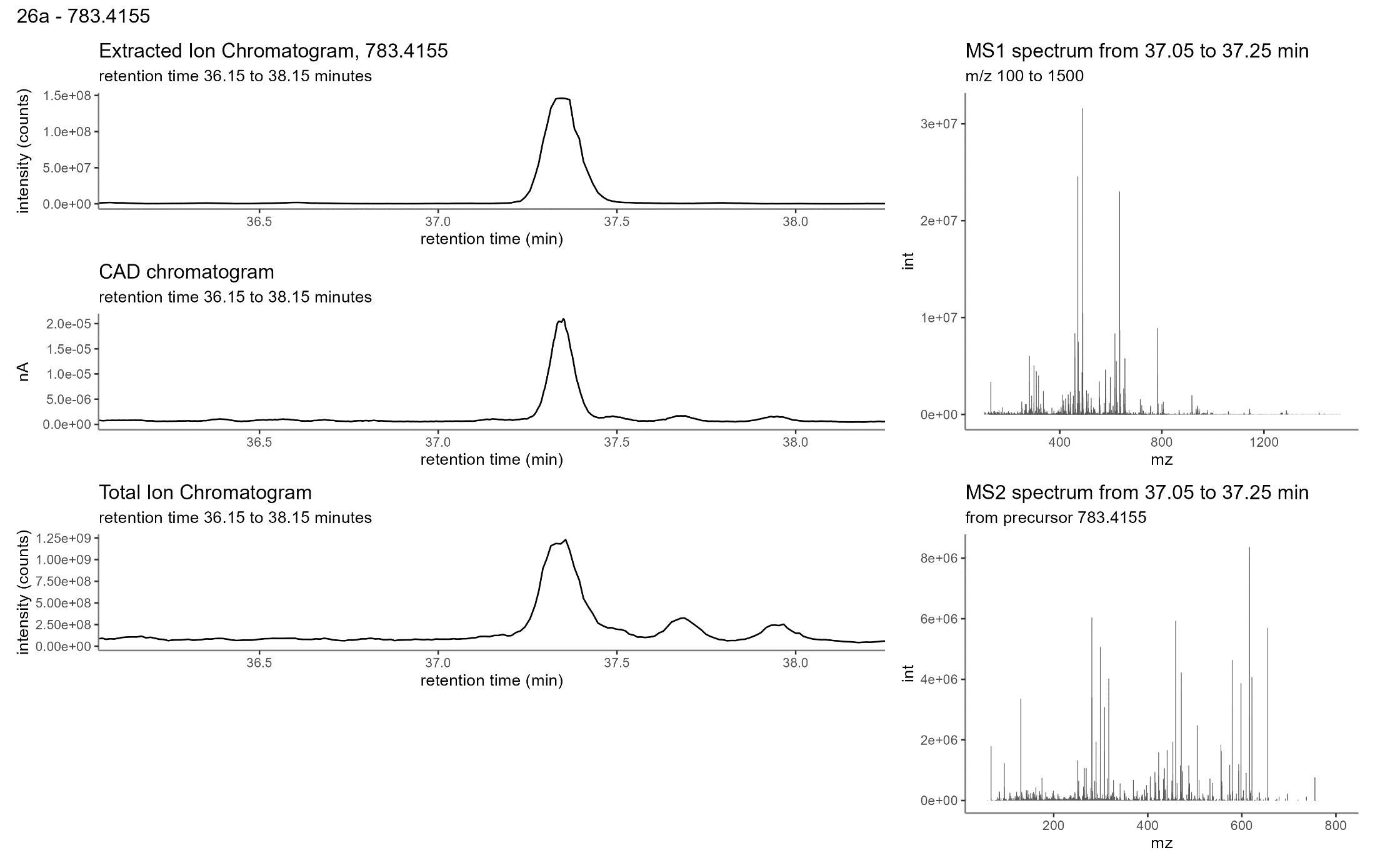 |
| 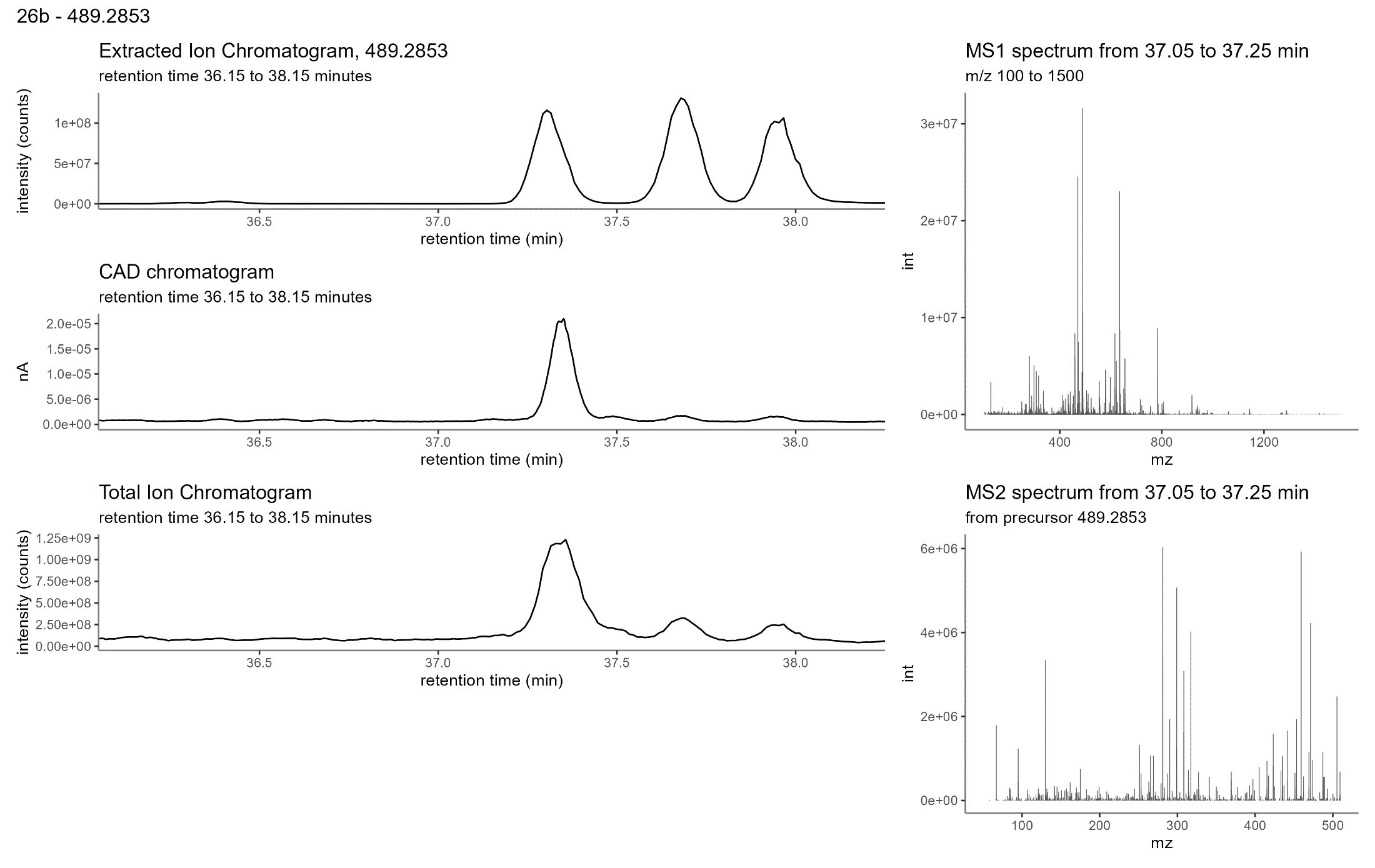 |
| 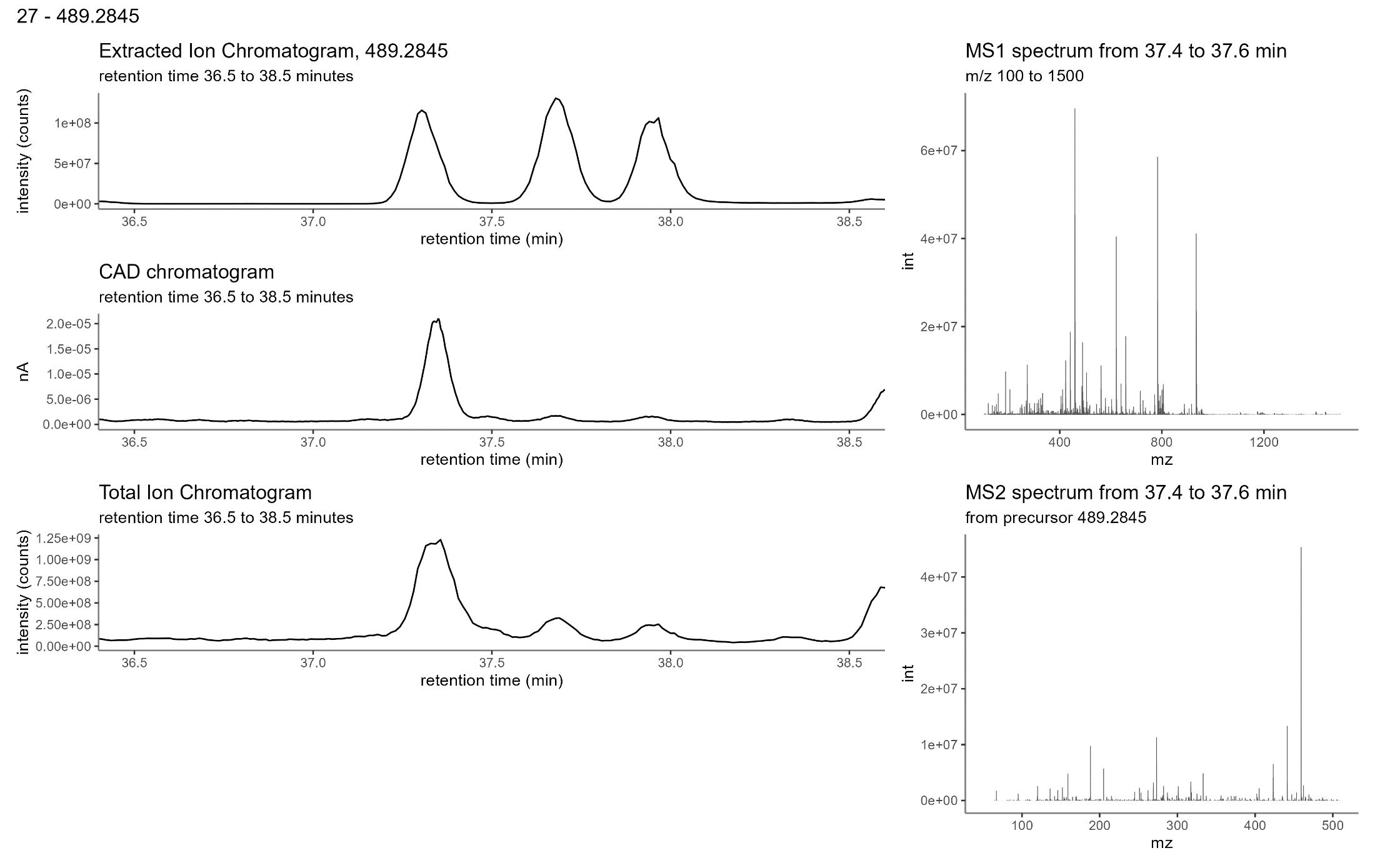 |
| 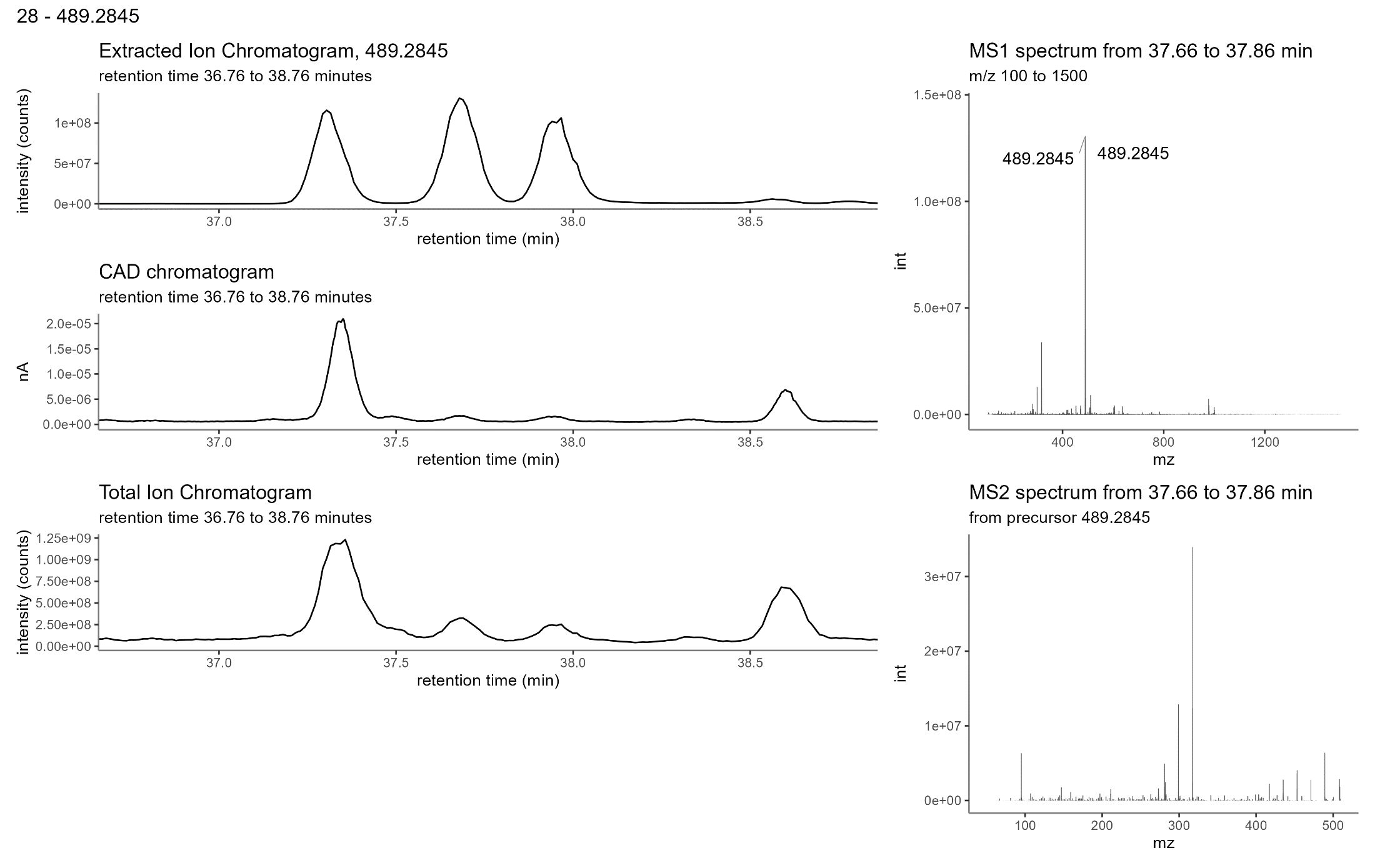 |
| 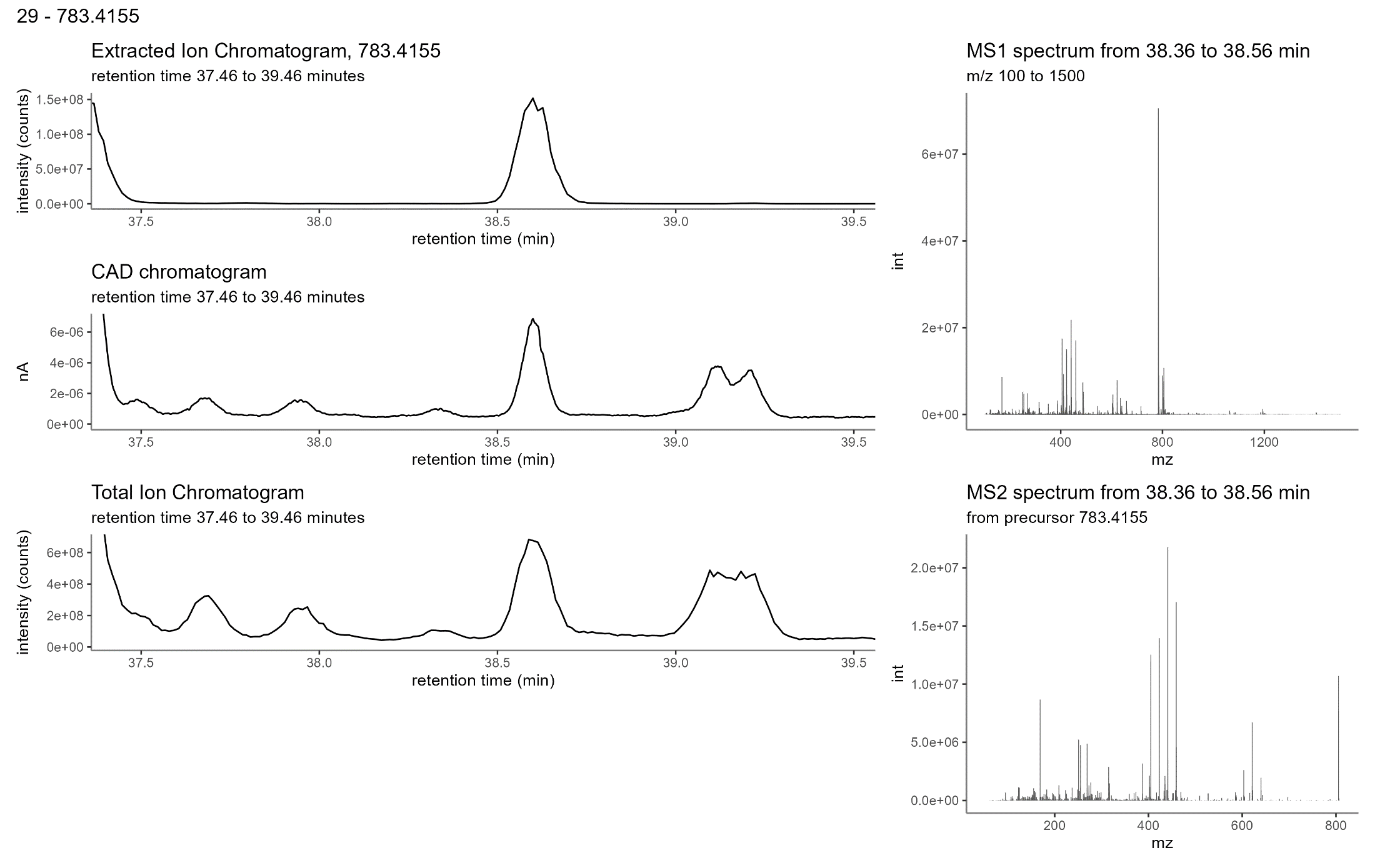 |
| 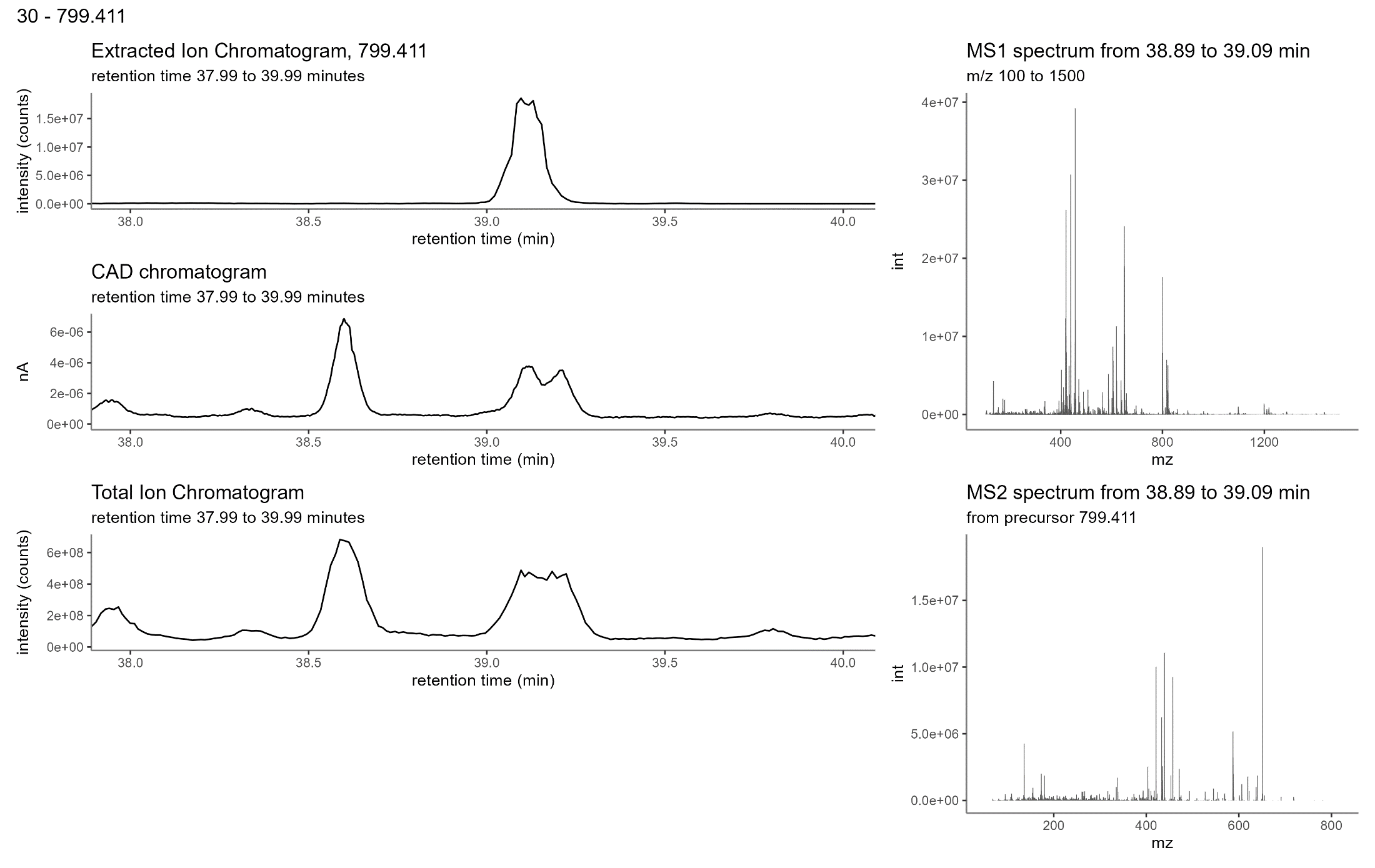 |
| 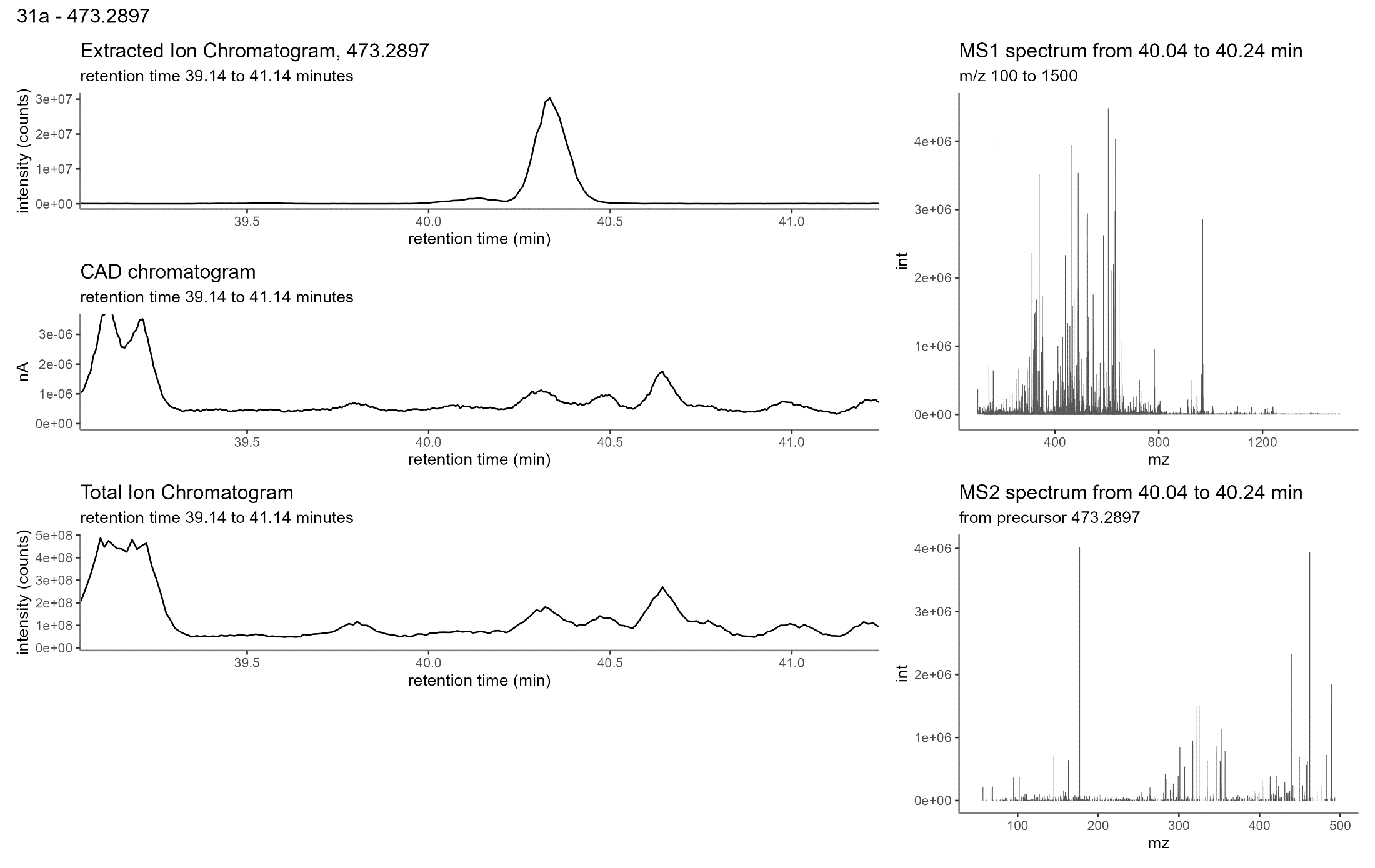 |
| 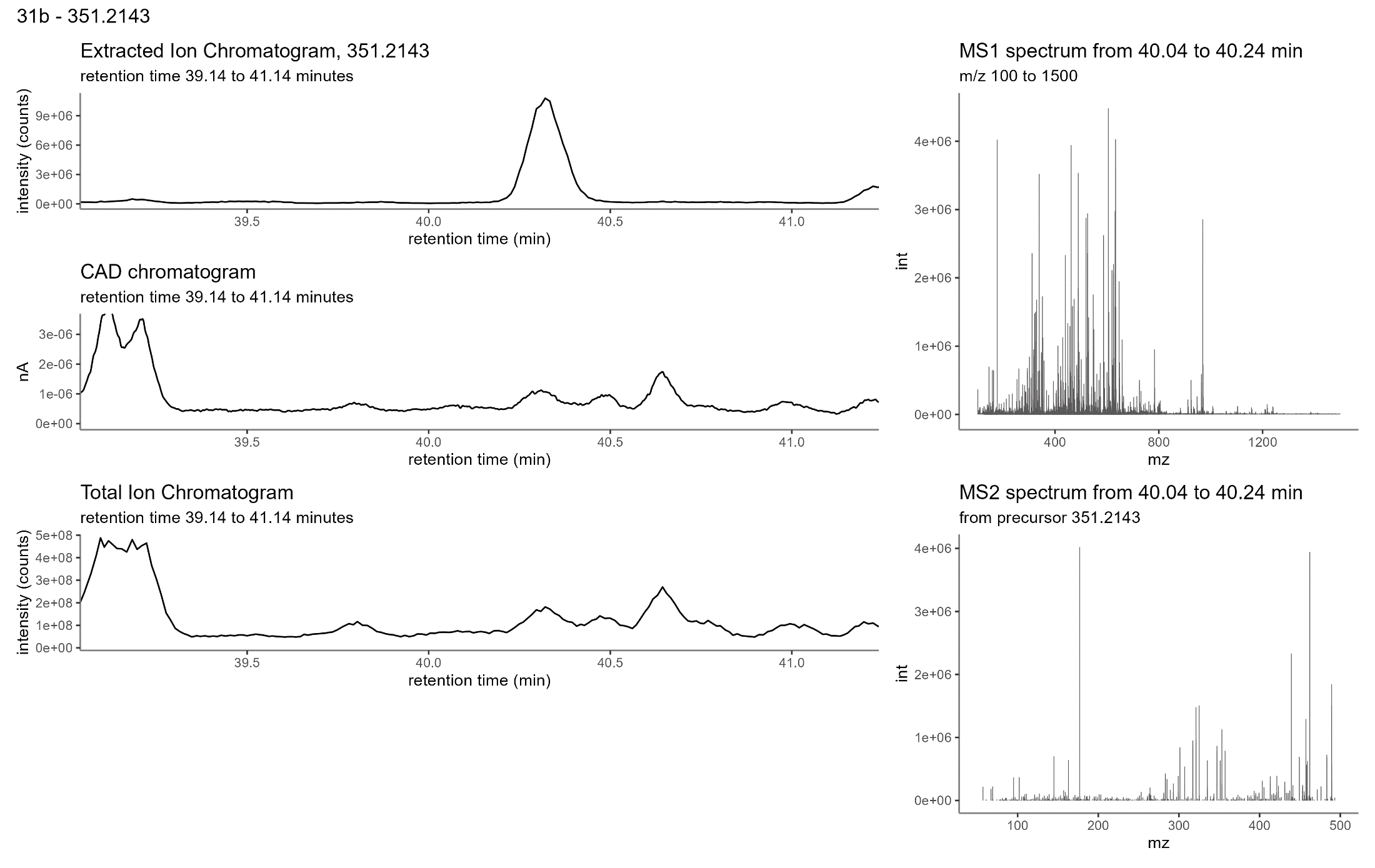 |
| 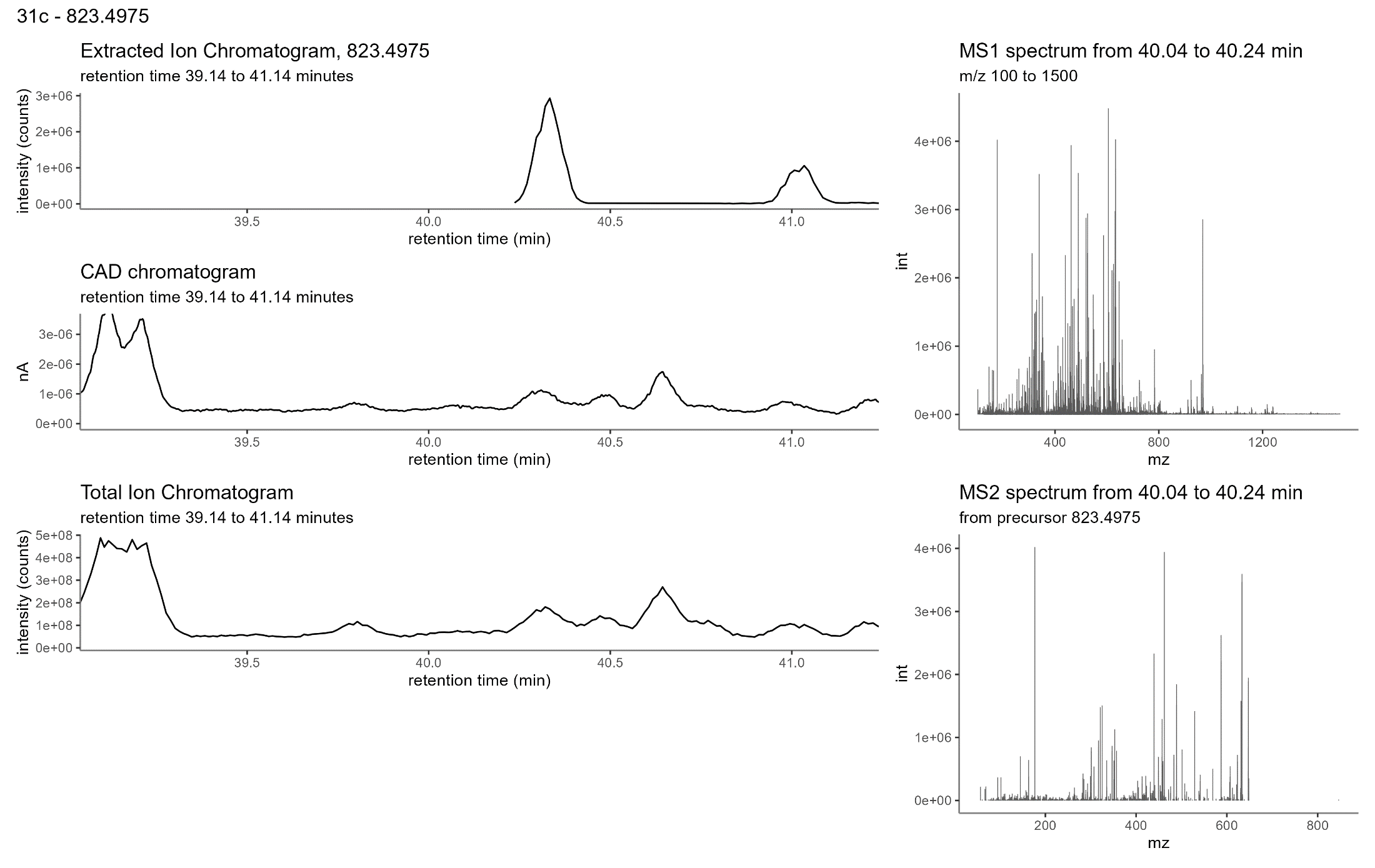 |
| 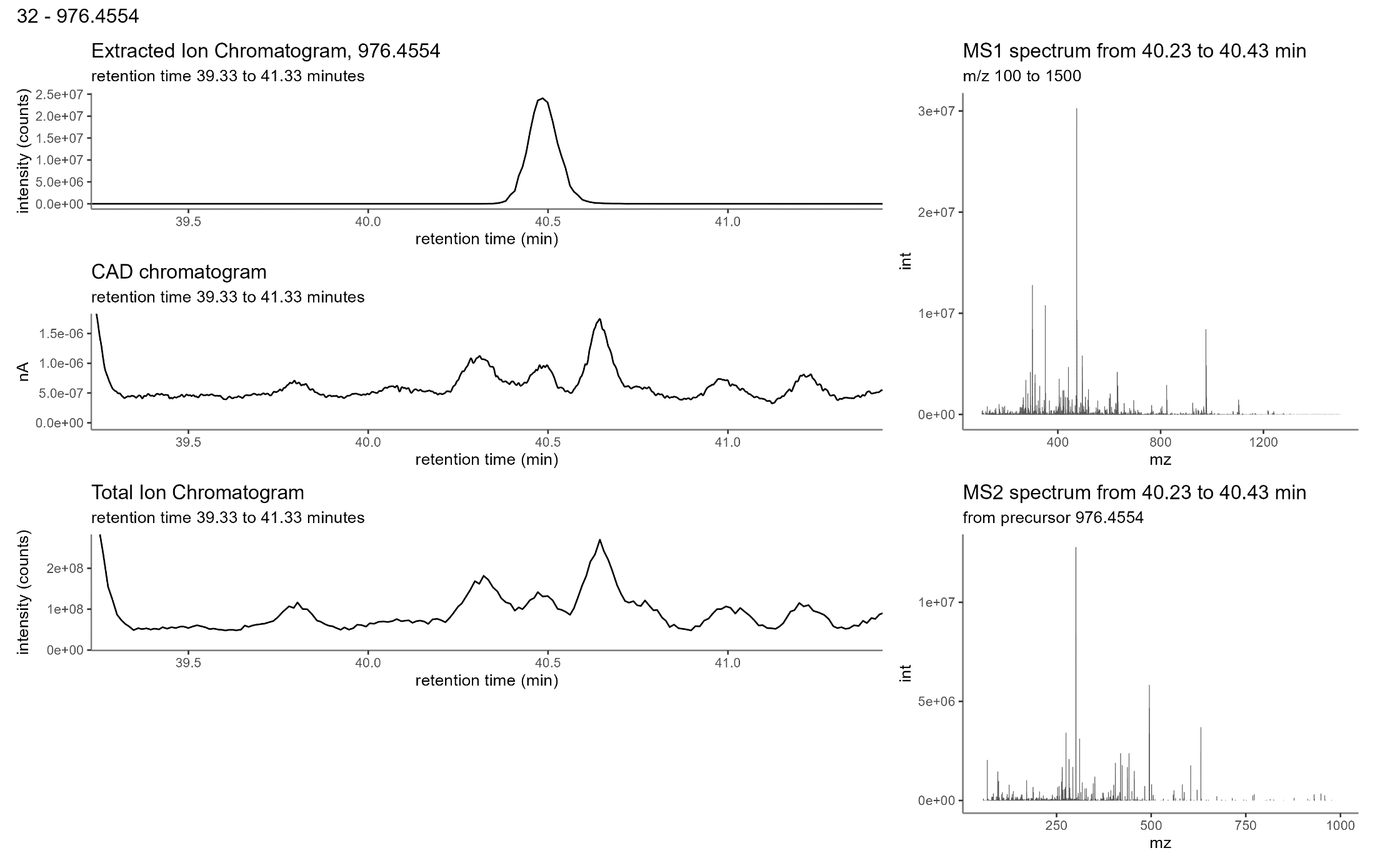 |
| 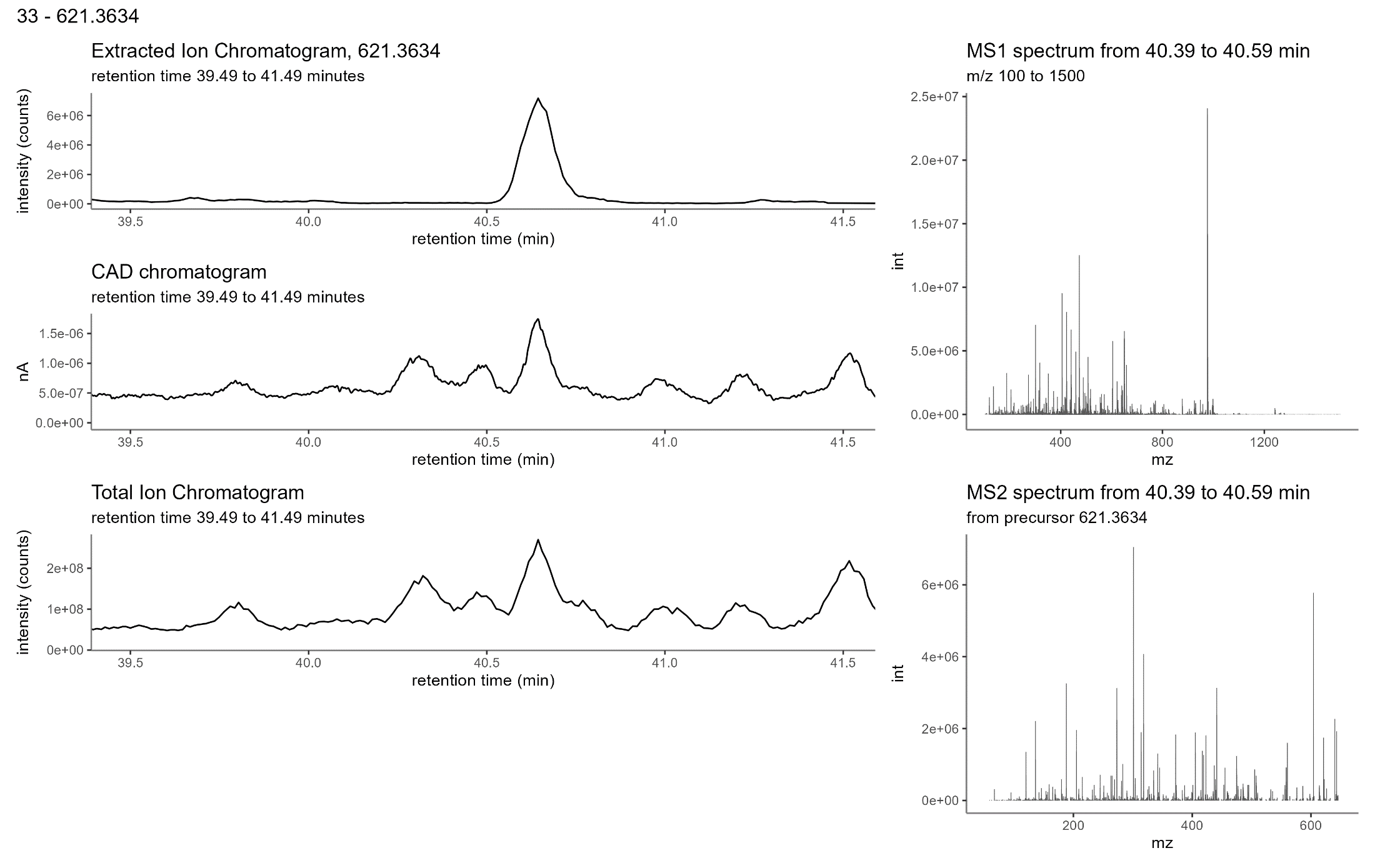 |
| 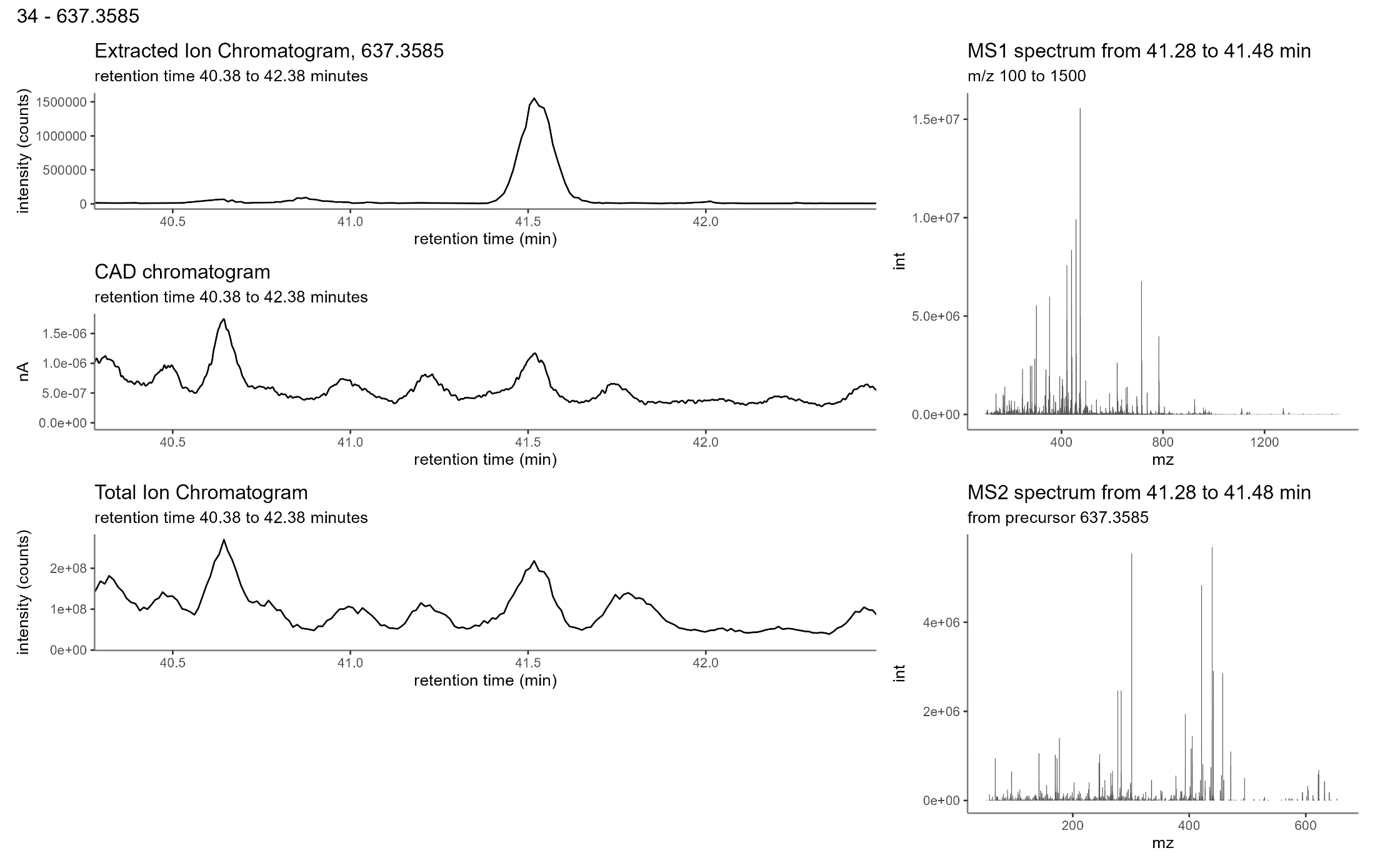 |
| 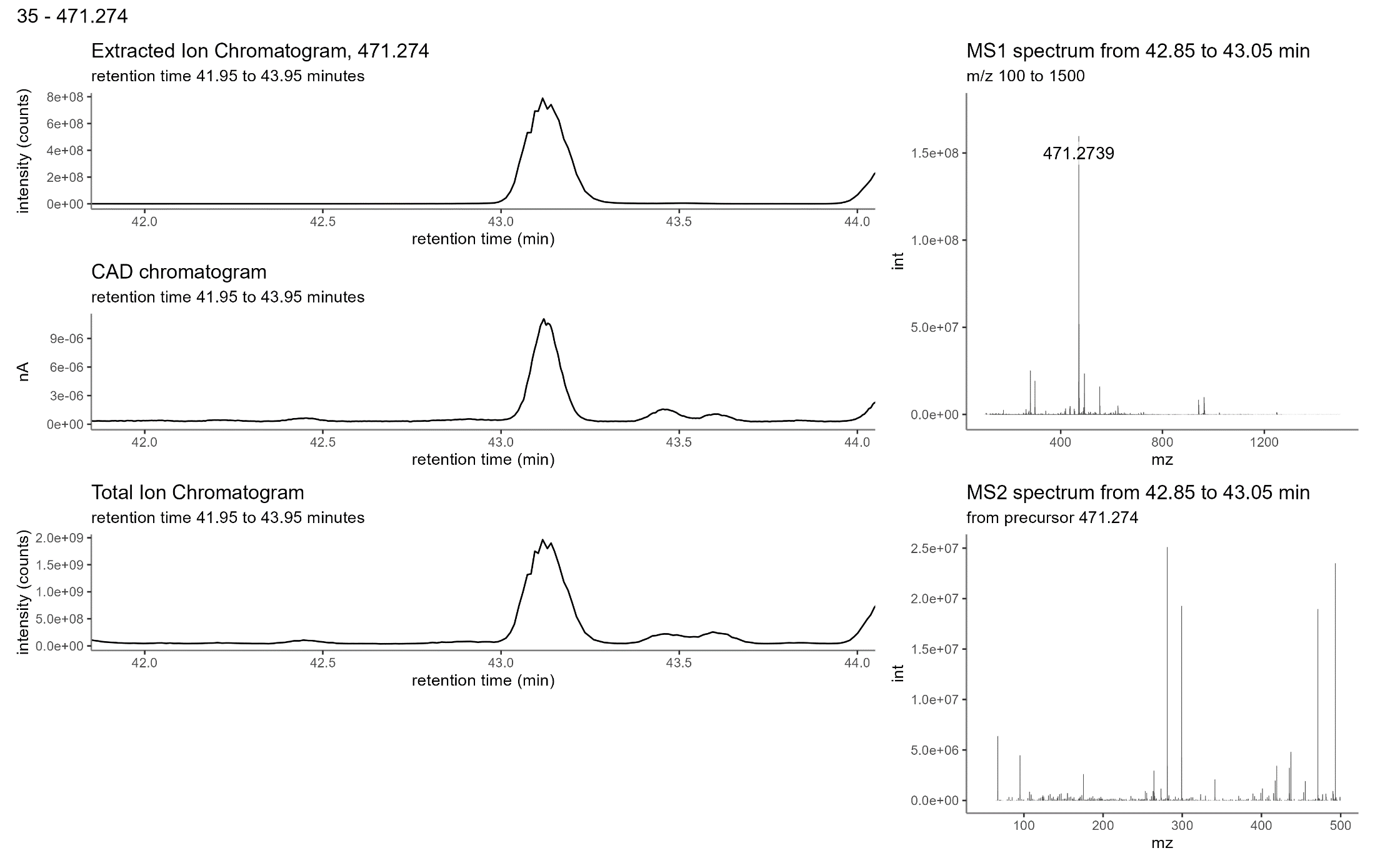 |
| 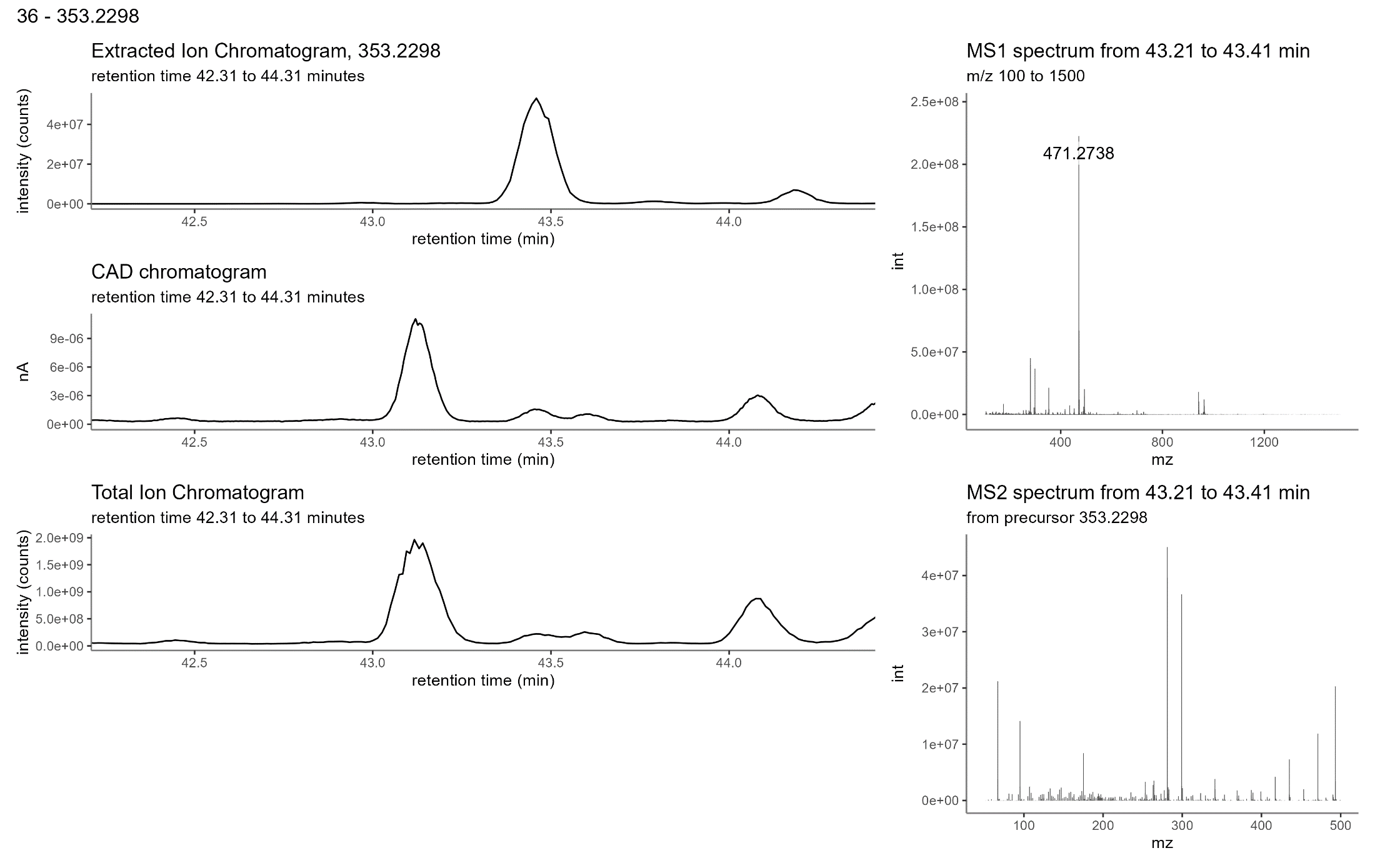 |
| 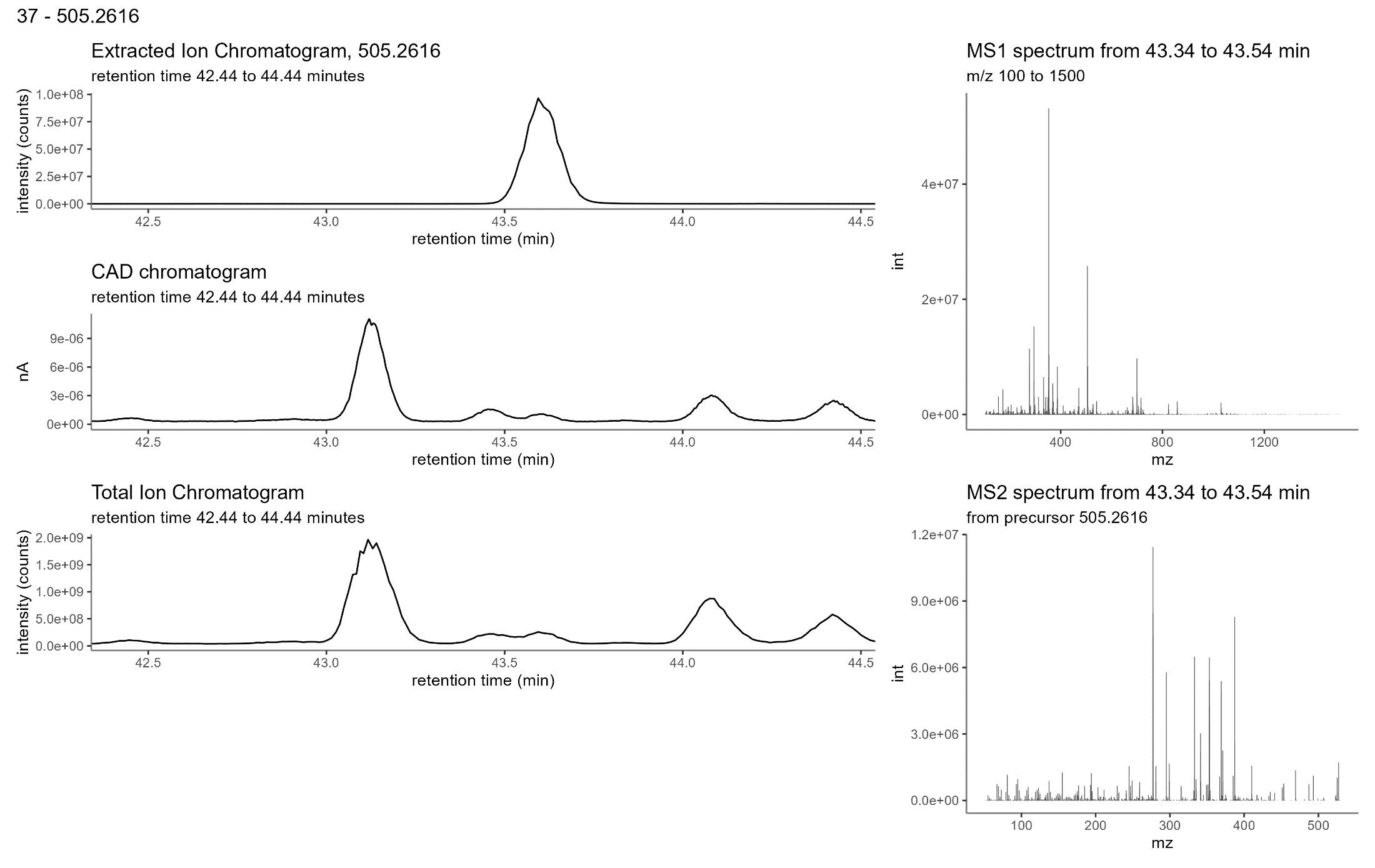 |
| 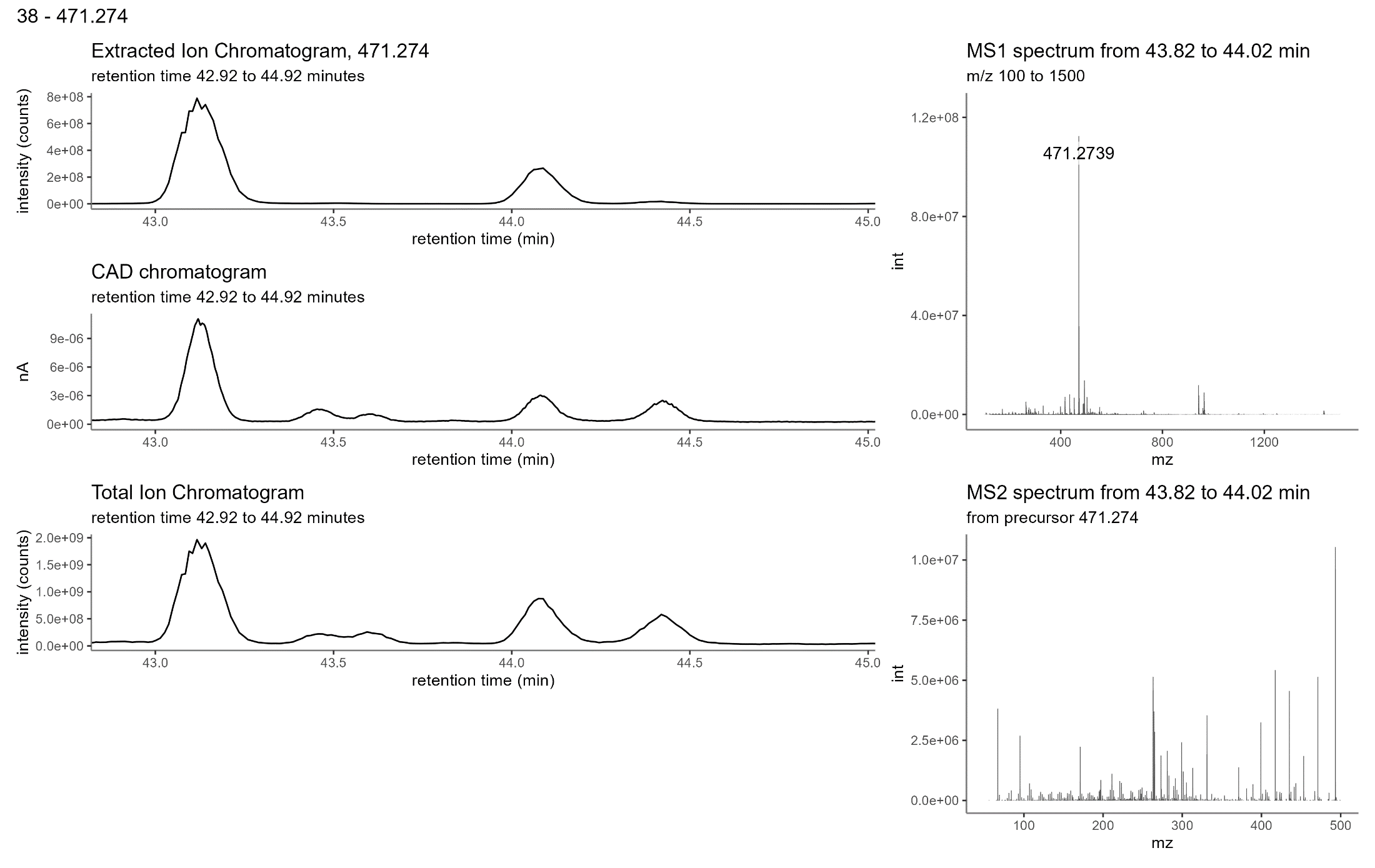 |
| 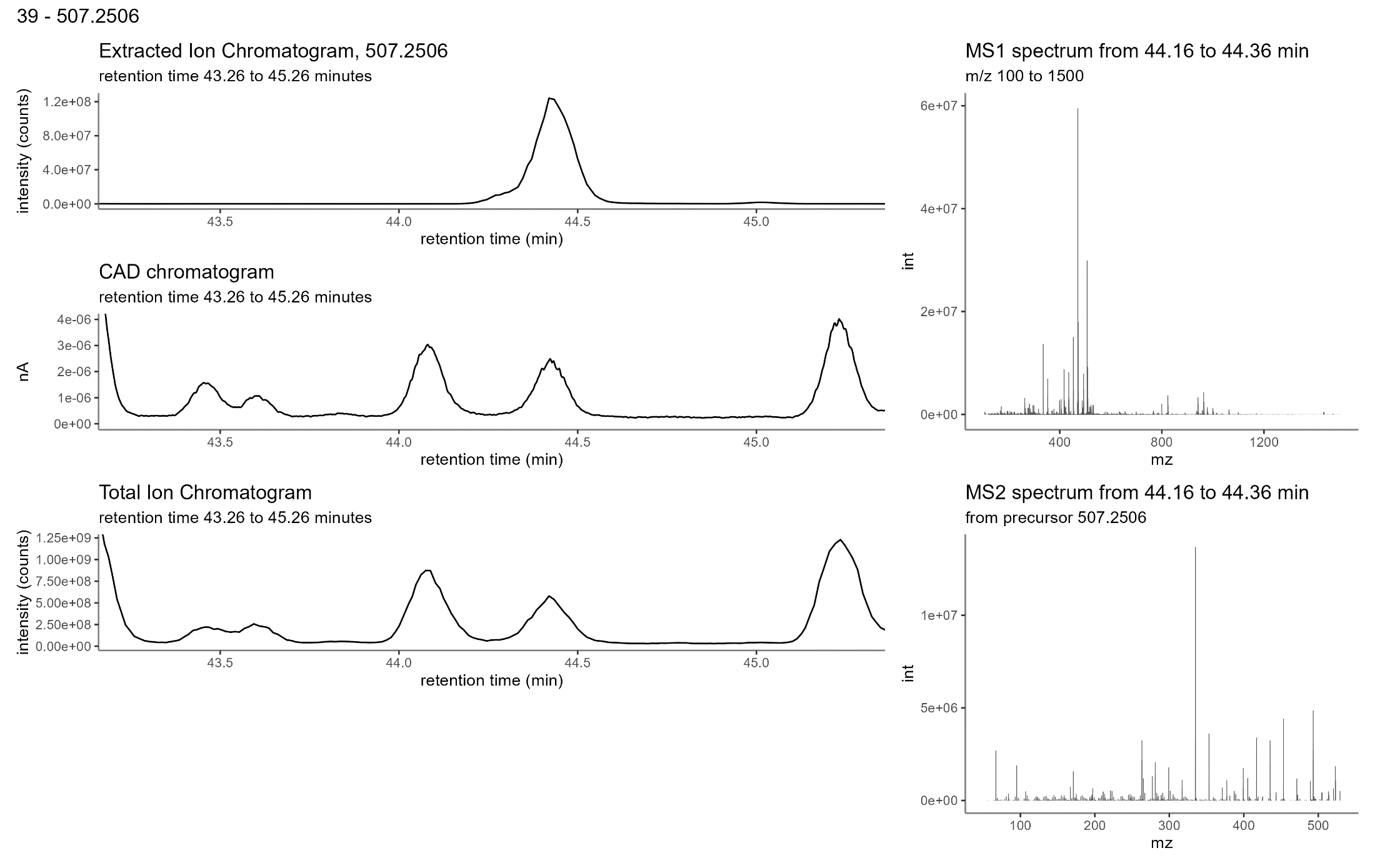 |
| 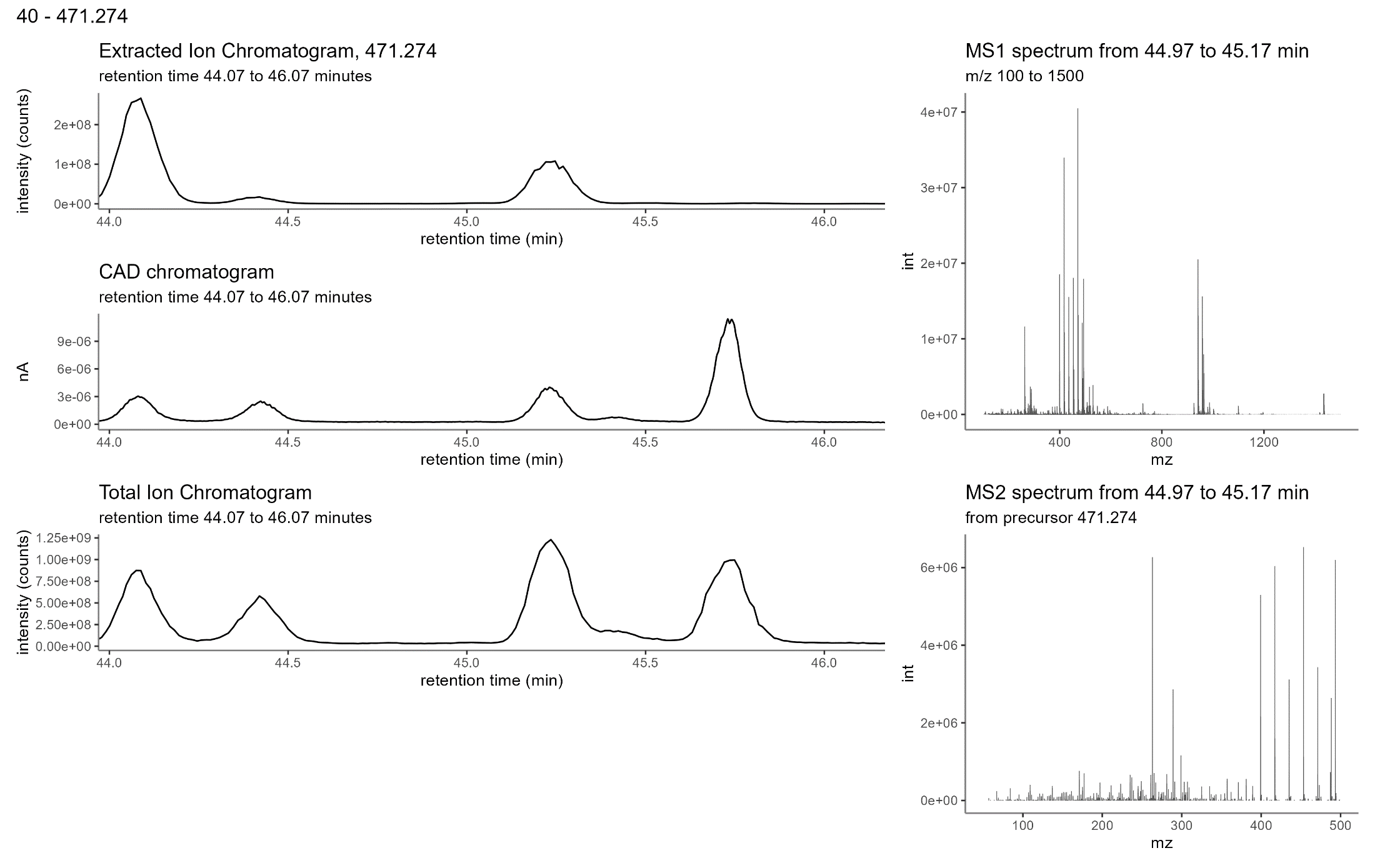 |
| 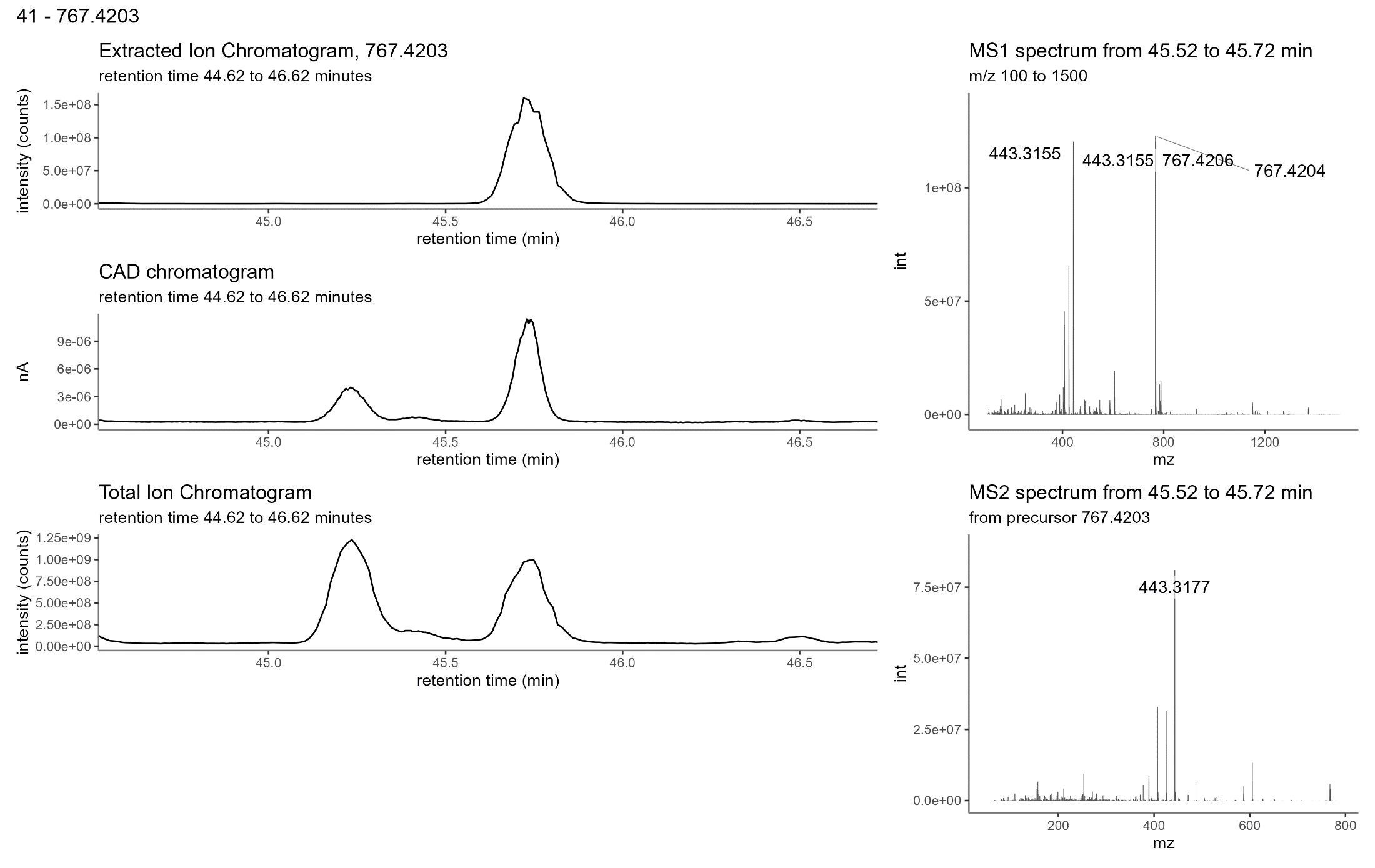 |
| 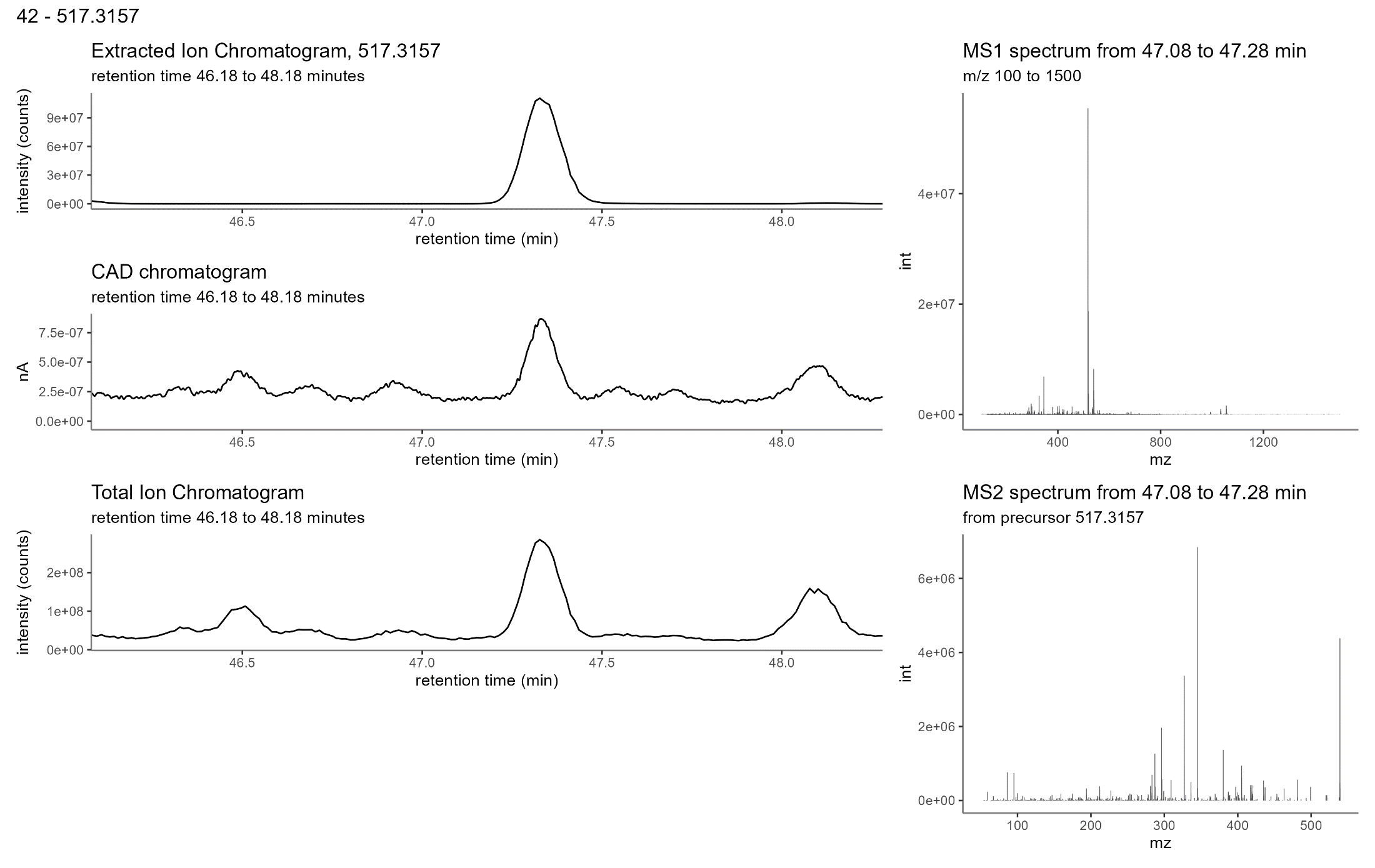 |
| 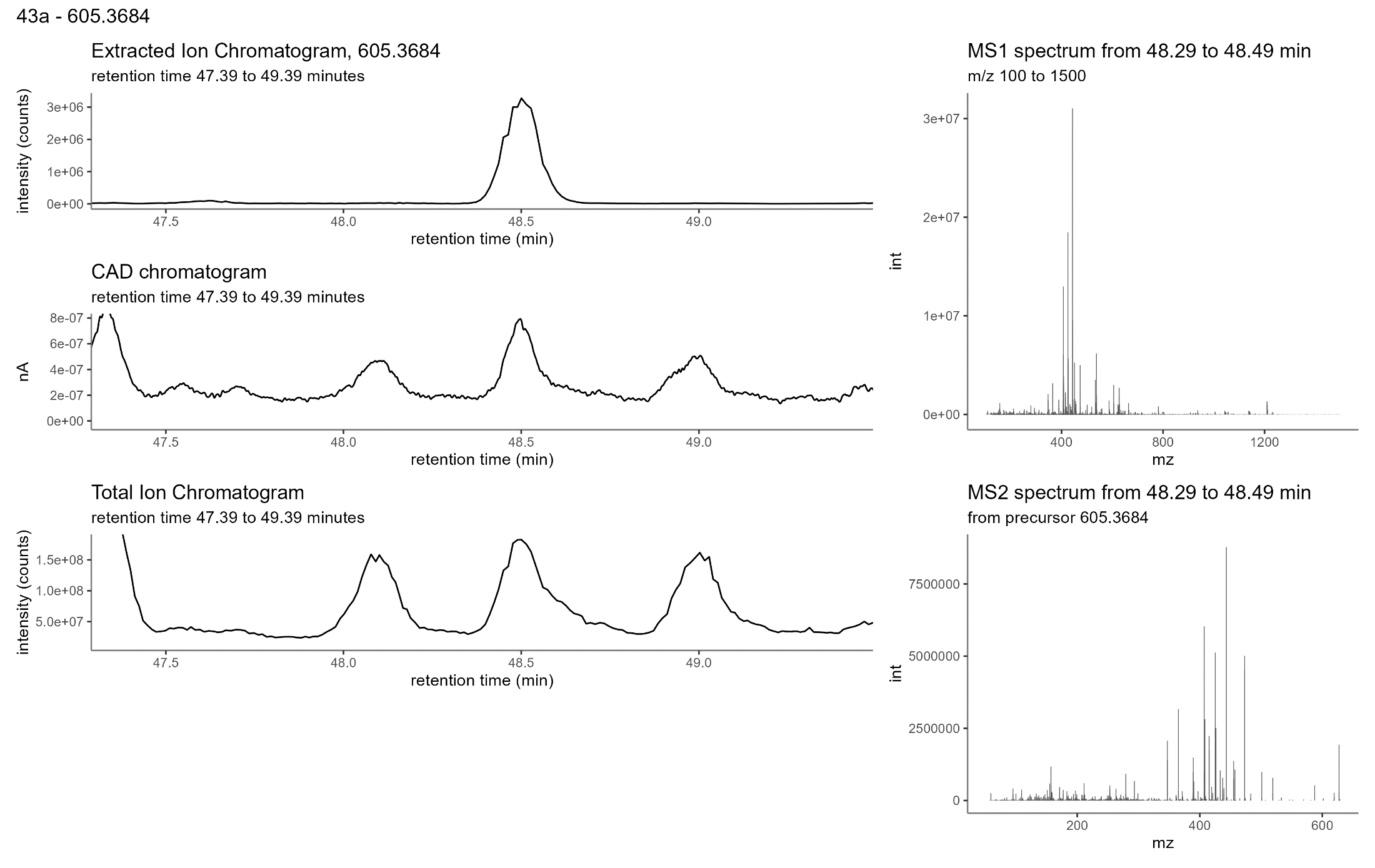 |
| 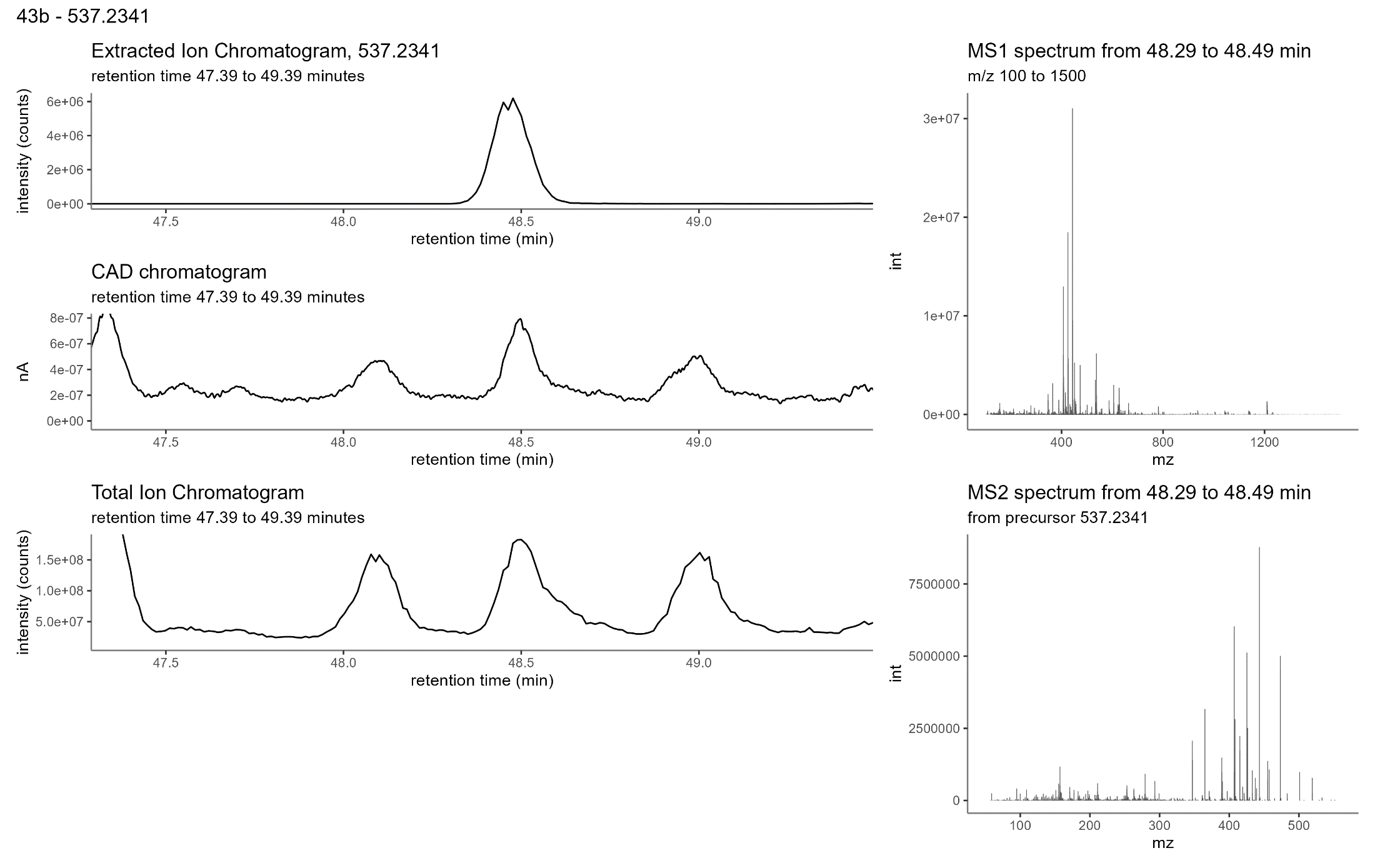 |
| 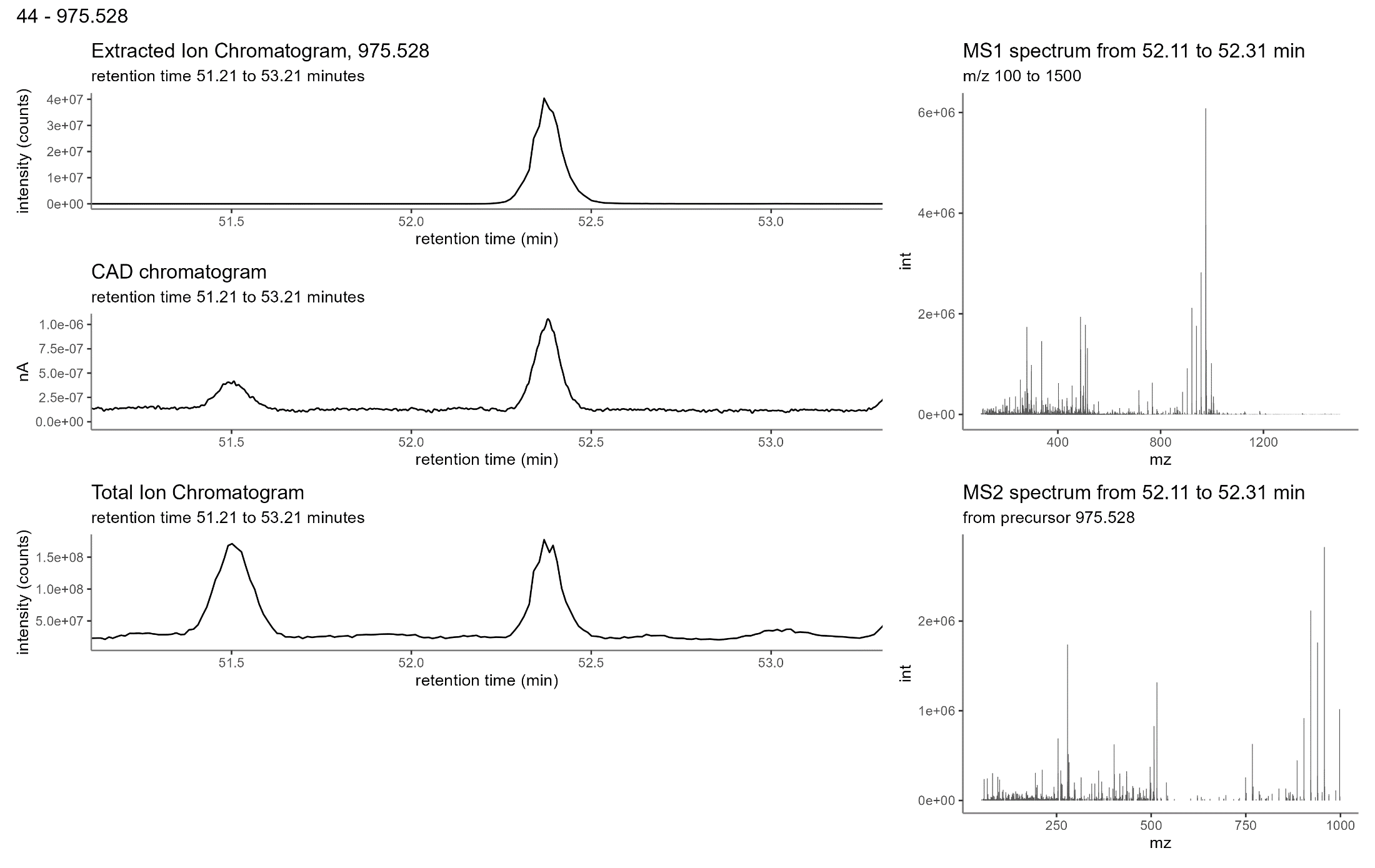 |
| 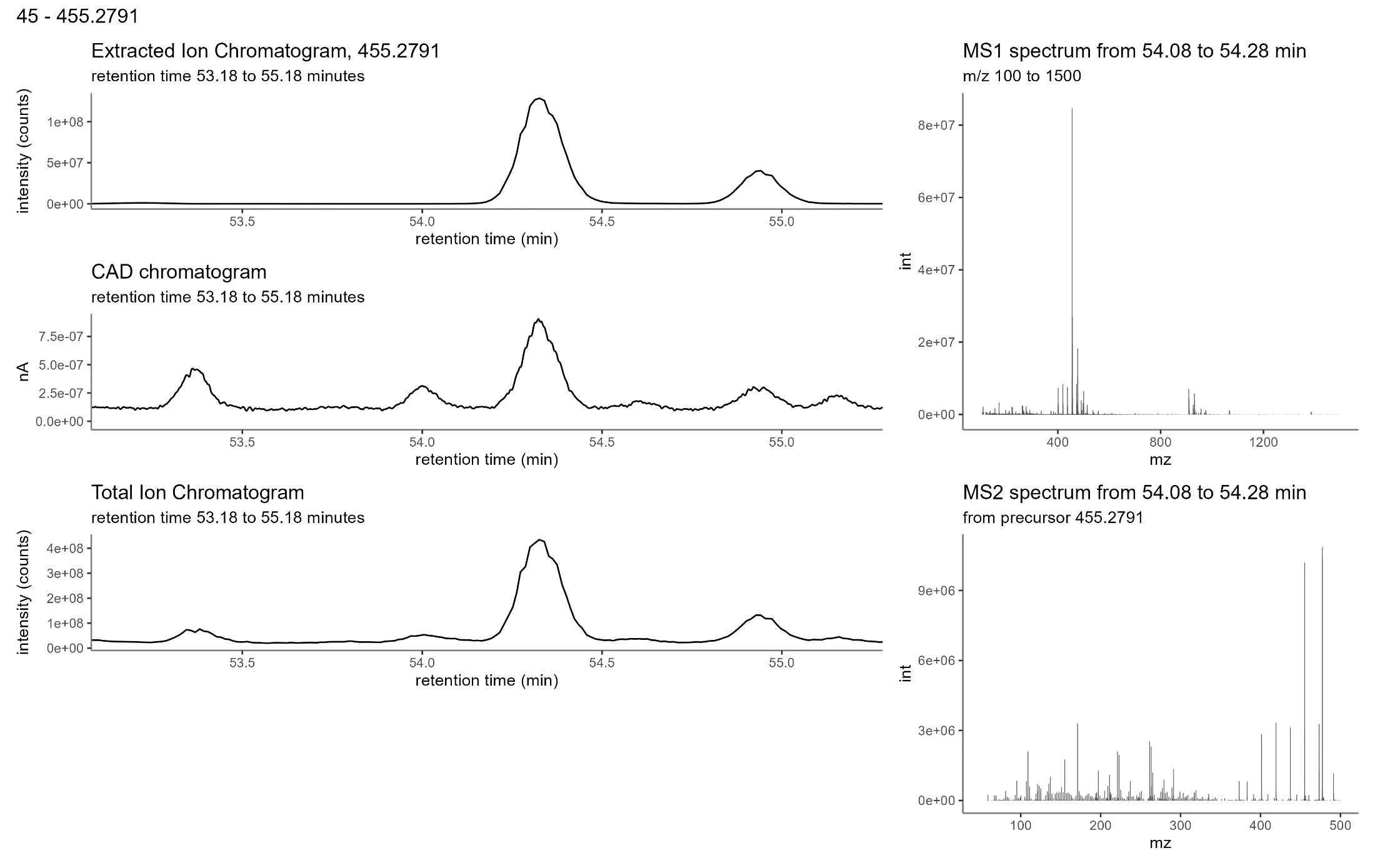 |
| 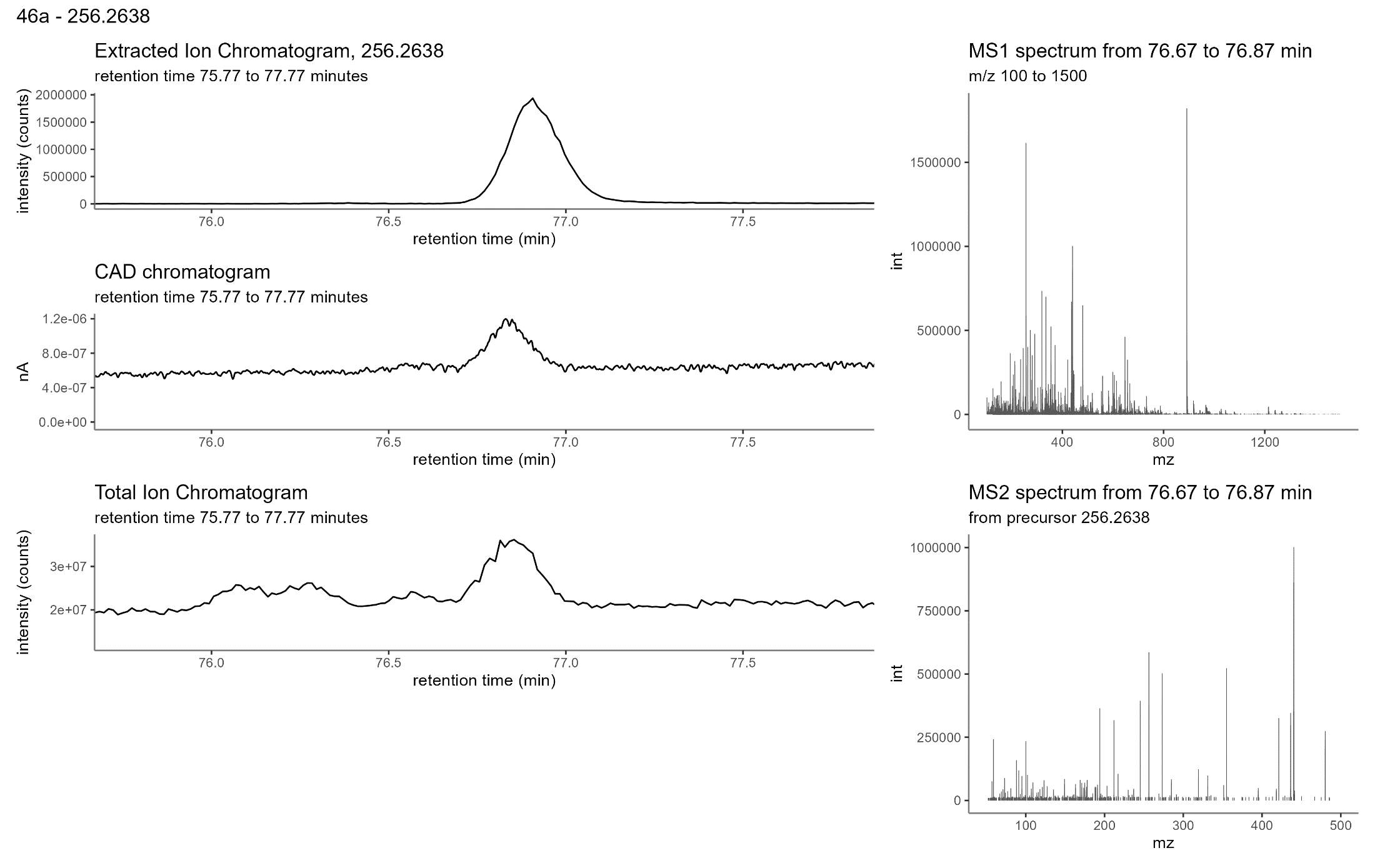 |
| 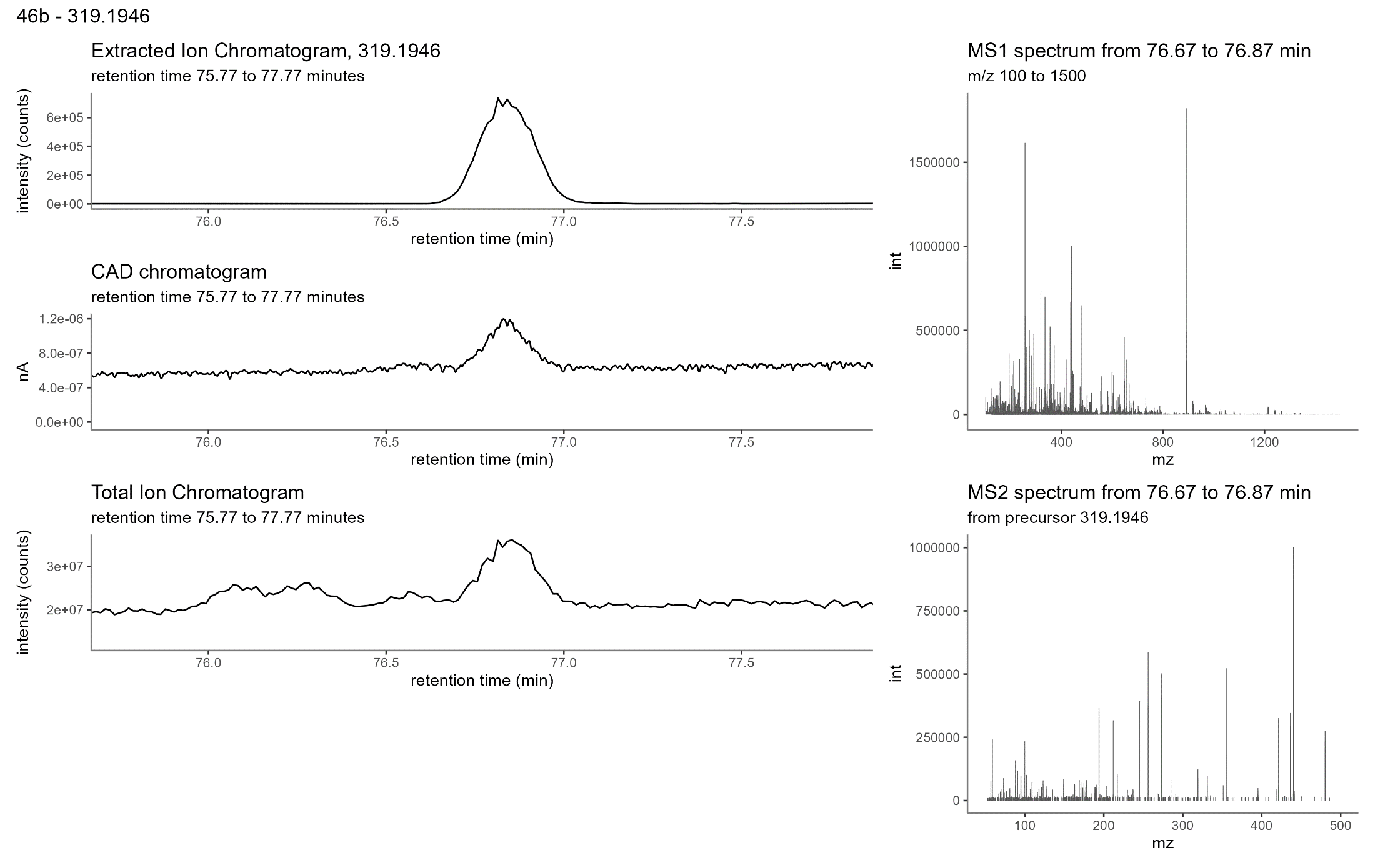 |
